# Attaching protein-adsorbing silica particles to the surface of cotton substrates for bioaerosol capture including SARS-CoV-2

**DOI:** 10.1038/s41467-023-40696-x

**Published:** 2023-08-18

**Authors:** Kieran Collings, Cedric Boisdon, Tung-Ting Sham, Kevin Skinley, Hyun-Kyung Oh, Tessa Prince, Adham Ahmed, Shaun H. Pennington, Philip J. Brownridge, Thomas Edwards, Giancarlo A. Biagini, Claire E. Eyers, Amanda Lamb, Peter Myers, Simon Maher

**Affiliations:** 1 Department of Electrical Engineering and Electronics, University of Liverpool, Liverpool, UK; 2 Department of Chemistry, University of Liverpool, Liverpool, UK; 3 Institute of Infection, Veterinary and Ecological Sciences, University of Liverpool, Liverpool, UK; 4 Centre for Drugs and Diagnostics, Department of Tropical Disease Biology, Liverpool School of Tropical Medicine, Liverpool, UK; 5 Centre for Proteome Research, Department of Biochemistry & Systems Biology, Institute of Systems, Molecular & Integrative Biology, University of Liverpool, Liverpool, UK; 6 Faculty of Health and Life Sciences, University of Liverpool, Liverpool, UK; 7 Applied Health Insights Ltd, Cheshire, UK

**Keywords:** Biomedical materials, Viral infection, Biomedical materials

## Abstract

The novel coronavirus pandemic (COVID-19) has necessitated a global increase in the use of face masks to limit the airborne spread of the virus. The global demand for personal protective equipment has at times led to shortages of face masks for the public, therefore makeshift masks have become commonplace. The severe acute respiratory syndrome caused by coronavirus-2 (SARS-CoV-2) has a spherical particle size of ~97 nm. However, the airborne transmission of this virus requires the expulsion of droplets, typically ~0.6–500 µm in diameter (by coughing, sneezing, breathing, and talking). In this paper, we propose a face covering that has been designed to effectively capture SARS-CoV-2 whilst providing uncompromised comfort and breathability for the wearer. Herein, we describe a material approach that uses amorphous silica microspheres attached to cotton fibres to capture bioaerosols, including SARS CoV-2. This has been demonstrated for the capture of aerosolised proteins (cytochrome c, myoglobin, ubiquitin, bovine serum albumin) and aerosolised inactivated SARS CoV-2, showing average filtration efficiencies of ~93% with minimal impact on breathability.

## Abstract (web summary)

There has been a significant increase in the demand for face masks, but achieving a balance of breathability and effective filtration is challenging. Here, the authors report a face mask of modified cotton for the capture of bioaerosols.

## Introduction

The global spread of severe acute respiratory syndrome coronavirus 2 (SARS-CoV-2) led to the introduction of lockdowns as well as other restrictions, including social distancing in an attempt to interrupt and slow transmission^1^. The restrictions had unprecedented and widespread socioeconomic impacts, however, these were consequences considered necessary to minimise transmission and prevent overcapacity of intensive care and other healthcare infrastructure^1,2^. The major route of SARS-CoV-2 transmission is via inhalation of airborne viral particles^3,4^. Contact transmission is believed to have a minor role in the transmission of the disease^5,6^. Airborne transmission is much harder to prevent, as respiratory droplets that contain the virus are produced from breathing, speaking, sneezing, and coughing^3,6,7^. For example, it has been demonstrated that respiratory droplets produced by talking are released at a rate as high as ~10,000 droplets per second^8^. It is estimated that up to ~40% of cases are asymptomatic^9–11^, yet studies have found this group to contribute to transmission via aerosols produced during breathing and speaking, and that they are similarly infectious as symptomatic cases^12^.

The particles produced by coughing have a typical size range of ~0.6–500 µm^13^. Larger droplets will typically only travel ~1–2 m as they are predominantly subject to gravitational forces^14,15^. Droplets of all sizes can become aerosolised and travel long distances, but smaller particles, including those smaller than 5 μm, are more likely to remain suspended in the air and travel farther^5,6,16^. One of the best defences against spreading SARS-CoV-2 infected particles is mask wearing^5^. Thus, the global COVID-19 pandemic caused a dramatic increase in the demand for face masks^17^, which has been impacted by stockpiling^2^.

The low availability of the N95 and surgical grade masks, as well as other factors relating to cost, re-usability and comfort, has popularised the use of homemade face coverings, often made from common fabrics such as cotton (amongst others). Some N95 respirators include a one-way valve to increase the breathability, however, it was demonstrated by Hazard et al. that the inclusion of a valve reduces filter efficiency relative to a valve-less N95^18^. Of course, this type of mask only filters air breathed in, and does not reduce virus transmission from an infected wearer to others. Testing in laboratory settings conducted at the University of Cambridge showed that a 100% cotton t-shirt showed a mean filtration efficiency of 70.7% for *Bacillus atrophaeus* compared to 96.4% for a surgical mask^19^. It was concluded that homemade face coverings should not be recommended to reduce transmission of infectious aerosols unless it was a last resort. It can only provide effective protection if paired with other methods of virus suppression such as isolation of the infected, social distancing, immunisation and hand hygiene^19^. Whilst materials such as cotton have been shown to offer limited protection^20^, a key reason for their popularity is the comfort, and apparent breathability afforded to the wearer^21^.

Since the outbreak of COVID-19, many researchers across the world have strived to make improvements to the performance, comfort, protection, and cost of face masks^22–25^. Electrostatics as a method of attracting and potentially inactivating pathogens has been demonstrated to improve the filter efficiency of household cotton materials, utilising triboelectric induction between woven fabrics^26,27^. Other research endeavours have sought to develop face masks with multi-functionality^28,29^, high reusability^28,30,31^, and anti-bacterial^32–35^ properties. For instance, a recent report used a thin glass layer formed by silica-resin coating technology for the purpose of imbuing surgical masks with antimicrobial agents^34^. Also, a number of reports have used different topologies including pathogen-inactivating metal‐based particles^36,37^, photocatalytic^36,38^, photothermal^32,36,39^ superhydrophobic^39,40^ and nanofibrous materials^24,40,41^.

Silica has long been used for protein purification^42–46^. Testing underivatised ‘bare’ silica against a range of proteins with different isoelectric points and hydrophobicities by Ghose et al. demonstrated the binding interactions between silica and proteins^42^. Depending on the protein’s molecular characteristics, either ionic or hydrophilic/hydrophobic interactions were dominant^42^. The addition of salt (disrupt ionic interactions) or ethanol (disrupt hydrophobic interactions) would change the binding interaction and hence the retention time of the proteins accordingly^42,43^. Silica is also widely used as a stationary phase for liquid chromatography as it can be packed into columns resulting in low back pressure and it has a particle size, pore structure and surface chemistry that can be readily controlled^47,48^. Yet, it is well known that silica has a high affinity for proteins^43,49,50^. It has long been a challenge for chromatographers to obtain fast and highly resolved protein separations due to the tendency of proteins to ‘stick’ to silica (in liquid chromatography columns).

Interestingly, coronaviruses, such as SARS-CoV-2, have an outer lipid membrane covered in protruding spike proteins that give the virus its distinctive ‘corona’ like appearance^51^. In general, proteins are strongly adsorbed to hydrophobic and hydrophilic interfaces by electrostatic interactions due to the patch-wise hydrophobic/hydrophilic characteristics of their three-dimensional surface. Thus, we hypothesised that amorphous mesoporous silica could be used to efficiently capture aerosolised proteins, and more generally bioaerosols, including SARS-CoV-2 by virtue of its protruding spike proteins.

The surface properties of amorphous silica depend on the presence of surface hydroxyl groups (isolated free, geminal free and vicinal (or bridge-bonded) silanols)^52^. The hydroxyl group forms a hydrogen bond with the adsorbates as a donor-receptor interaction. The structure of a silica microsphere is a network of siloxane bonds (Si-O-Si), which would be considered slightly hydrophobic, however the siloxanes are sterically hidden behind the surface ligands and silanols^42^. A quaternary amine (QA) ligand on the surface of the silica microspheres can be bonded to the cellulose of a cotton substrate^53,54^. At neutral and pH > 4, the silanols exist as the anionic SiO^-^, while the QA will retain the N^+^, giving a wide range of positive and negative species that can be attracted and bound.

In this article, we propose an original approach for the capture of bioaerosols, including airborne viruses such as SARS-CoV-2, based on the principle of adsorption. The premise of this research is borne out of a long running problem in the field of liquid chromatography whereby proteins tend to ‘stick’ to silica (in columns), and the fact that the SARS-CoV-2 virus has an outer lipid membrane covered in spike proteins. Furthermore, as part of our design, we idealise the possibility of a face covering that exhibits a high filtration efficiency for particular airborne particulates (i.e., SARS CoV-2), whilst retaining sufficient breathability (i.e., comfortable for the wearer). We surmise that a modified cotton substrate can provide an ideal framework for a general covering; cotton is an environmentally friendly fibre, it is sustainable, renewable, biodegradable and widely available. This, combined with its breathability and limited filtration performance, makes cotton an exemplary substrate for further modification.

In this work, we investigate an air filter, which we have configured as a face covering, using mesoporous silica microspheres bonded to a cotton substrate, thereby offering a viable, environmental-friendly and simple solution to minimise the spread of potentially infective bioaerosols. Our analysis of the silica coated material focuses on: i) filtration efficiency for aerosolised proteins, in general, and also aerosolised SARS CoV-2, and, ii) breathability, as characterised by the pressure differential across the face covering. An idealised face covering should be sufficiently effective at filtering unwanted substances with little/no breathing restriction. To aid comparison and wider discussion, we proceeded to test silica-bonded cotton material against a blank cotton substrate (blank control) without any silica coating, as well as cotton face covering that is freely distributed to students by the University of Liverpool (representative of publicly available cloth face coverings).

## Results and discussion

### Selection of particle size and silica form

General design criteria are based on inhalation safety, filtration effectiveness and breathability. It is well-known that repeated exposure to crystalline silica dust can lead to silicosis, which can be fatal^55^. However, in this project, we use amorphous silica. Based on a report from the US Agency for Toxic Substances and Disease Registry^56^, studies of amorphous silica in workers and laboratory animals have not found evidence of cancer. There are no known health effects from exposure to amorphous silica at the levels found in the environment or in commercial products.

**Fig. 1:**
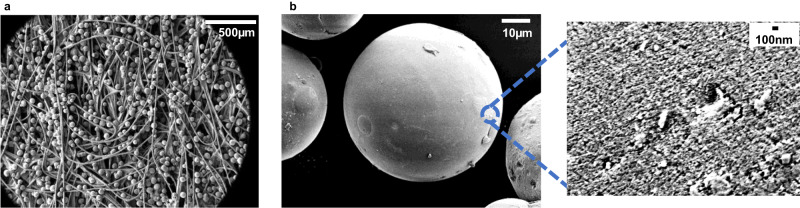
SEM images of QA functionalised silica. **a** QA functionalised silica attached to the substrate, and **b** silica particle (x1300) with a magnified image showing pores visible on the surface (x30,000). All data shown are representative of at least three independent experiments.

In this study, an amorphous mesoporous spherical silica was produced with a specific particle size and porosity: a diameter of ~50 µm (Supplementary Fig. S1, Supplementary Table S1) and pore sizes of ~11 nm (Fig. S2a). Although smaller silica particle sizes ( < 50 µm in diameter) can increase the overall surface area available and can likely improve capture efficiency of aerosolised proteins and virus, they may increase the risk of inhalation of silica dust. Thus, silica with a diameter of ~50 µm was chosen for this study as a precautionary measure since it can be readily retained in a non-woven gauze that has a suitable density, physically forming a barrier to prevent silica loss. Primarily though, the silica utilised in this study was attached to the substrate (as seen in Fig. 1).

### Porosity of amorphous silica

**Fig. 2:**
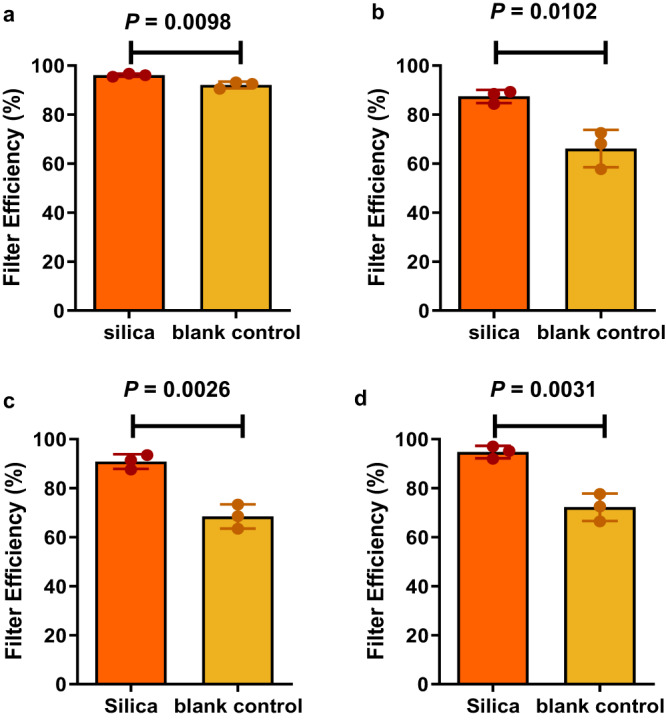
Filter efficiency of 4 different aerosolised proteins for the silica-coated cotton material and blank control cotton material. **a** Myoglobin, **b** BSA, **c** Ubiquitin, **d** Cytochrome c. All data are derived from three independent experiments (mean ± SD, *n* = 3). Comparisons were performed with two-tailed Student’s *t*-test.

The porosity of silica can be altered to significantly increase the effective surface area and its interaction with different sized particles. For conventional masks, small particles are more likely to be filtered due to diffusion mechanisms and electrostatic interactions while larger particle sizes are filtered by interception and inertial impaction^57^. In our design, by ensuring a silica pore size that is smaller than the expected diameter range for the virus, smaller analytes can be drawn into the pores by capillary action, leaving the virus adsorbed on the outer spherical surface. This is an important consideration since viral particles are carried in exhaled droplets and the resulting dry particles. Viruses such as SARS-CoV-2 usually have diameters ranging from 50 to 200 nm, with the COVID-19 spherical virion being approximately 97 nm in diameter^58–61^. Moreover, if one considers design choices in liquid chromatography, larger pore sizes ( ~30–100 nm) are often required for better separation of large proteins (molecular weight up to ~200 kDa)^62,63^, so that the analyte can be diffused into the pores. Therefore, in our study, to promote smaller molecules being drawn into the pore structure of the spherical silica, leaving viral particles adsorbed on to the outer surface, a pore size of ~10 nm was chosen for this investigation. The non-functionalised silica used in this study has a surface area of 320.24 m^2^ g^−1^, a mean pore volume of 0.96 cm^3^ g^−1^ and a mean pore diameter of 11.48 nm (Supplementary Fig. S2a). The BET isotherm, which is correlated to surface area, displays a Type IV H2 hysteresis (Fig. 2b), indicating capillary condensation in mesopores^64,65^. Further details about the measurement methods and graphical results can be found in the Supplementary Methods and Supplementary Fig. S2a.

### Surface charge of silica with QA functionalisation

The zeta potential gives an estimate of the surface electrical charge of the shear surrounding colloidal particles in a suspension^66,67^. Supplementary Table S3 shows the zeta potential was affected by heat treatment (i.e. ~800 °C) to silica and when the quaternary amine ligand was attached to the silica surface. The QA-functionalised silica used in this paper generated an average positive zeta potential of + 15.6 mV when exposed to water, which can be attributed to the positive charge contribution from the QA ligand (N^+^).

When these silica particles underwent a vigorous heat treatment, some degree of dehydroxylation of the surface (i.e., the removal of silanol groups) occurs^52^. There is also a rearrangement of silanols with more pronounced isolated silanol character when silica is heated to high enough temperatures (e.g. ≥ 800 °C)^68^. This results in an increased positive zeta potential ( + 31.8 mV), leaving more readily available isolated silanols for QA functionalisation (Supplementary Table S3).

### Morphology and stability of silica-coated substrate

In order to test our hypothesis that silica coated cotton could act to capture aerosolised coronavirus particles, the mesoporous silica was functionalised with a QA providing a pathway to fulfil our primary design goals (i.e., effective capture of SARS CoV-2 whilst ensuring a breathable material). SEM images show an even distribution and attachment of ~40–60 µm QA-functionalised silica to the substrate fibres (Fig. 1a). On a cotton substrate, the interaction between cotton (cellulose) with QA-SiO2 that attaches the silica particles is shown in Supplementary Fig. S7b, where a condensation reaction occurs between silanol groups (-Si-OH) on the surface of the QA-functionalised silica and C-OH groups of cellulose under thermal treatment to form Si-O-C bonds^69,70^. In the literature there are a plethora of reports detailing a variety of other ways in which silica can be bound to a wide range of substrates. Results from a preliminary stability test indicate that the silica swabs stored under ambient conditions for one month exhibited no significant difference in filter efficiency of aerosolised proteins, compared to freshly prepared swabs (Supplementary Fig. S3, *p* > 0.05). This suggests that the silica swabs remain stable and useable for at least one month.

### Size distribution of bioaerosols

A portable ultrasonic nebuliser (Ortorex) was used to generate aerosols from the test working solutions and used for all filter efficiency tests unless stated otherwise. The particle size distribution of the aerosol was measured for eight solutions at 0.1 mg/mL concentration. They were pure water, cytochrome c, BSA, ubiquitin, myoglobin, inactivated SARS-CoV-2 (1.3 × 10^5^ PFU/mL), creatinine and caffeine. The results showed that all solutions had very similar distributions, as shown in Supplementary Fig. S4. The most frequent particle diameter band for all test solutions is ~0.3 µm. Characterisation of the nebuliser showed that most particles were smaller than 1 µm (Supplementary Fig. S4), and thus can be considered to be aerosolised, which is broadly consistent with the data reported by the manufacturer^71–73^.The refractive indexes of all testing materials were very similar to that of pure water (Supplementary Table S4), indicating that the laser light scattering for particle size distribution measurements was not affected by the internal properties of the aerosols.

### Filter efficiency of silica-coated cotton material for common aerosolised proteins

The underpinning hypothesis upon which this work rests, is the capability of silica to adsorb protein. If this could be demonstrated for aerosols, then it would open the possibility of capturing the SARS-CoV-2 virus by virtue of the surface proteins on the particle. To test the capability of silica to adsorb protein, we set out by testing with a variety of generic aerosolised proteins. These were compared directly against an identical cotton substrate without any modification. Figure 2 shows the significant increase in filter efficiency for the silica coated cotton material relative to the uncoated standard cotton control for the four proteins tested. The filter efficiencies of the silica coated cotton for aerosolised myoglobin, bovine serum albumin (BSA), ubiquitin and cytochrome c were 97%, 87%, 91%, and 95%, respectively. This demonstrates an average increase in filter efficiency of ~17% relative to the blank cotton material due to the addition of the silica.

To demonstrate that the silica is more likely to adsorb proteins over other compounds and that it is not just accumulating moisture indiscriminately, these materials were also tested for filter efficiency with other small polar molecules dissolved in water (relatively hydrophilic creatinine^74,75^ and slightly hydrophobic caffeine^75^) whereby a relatively lower increase in filter efficiency was observed (Supplementary Fig. S5).

**Fig. 3:**
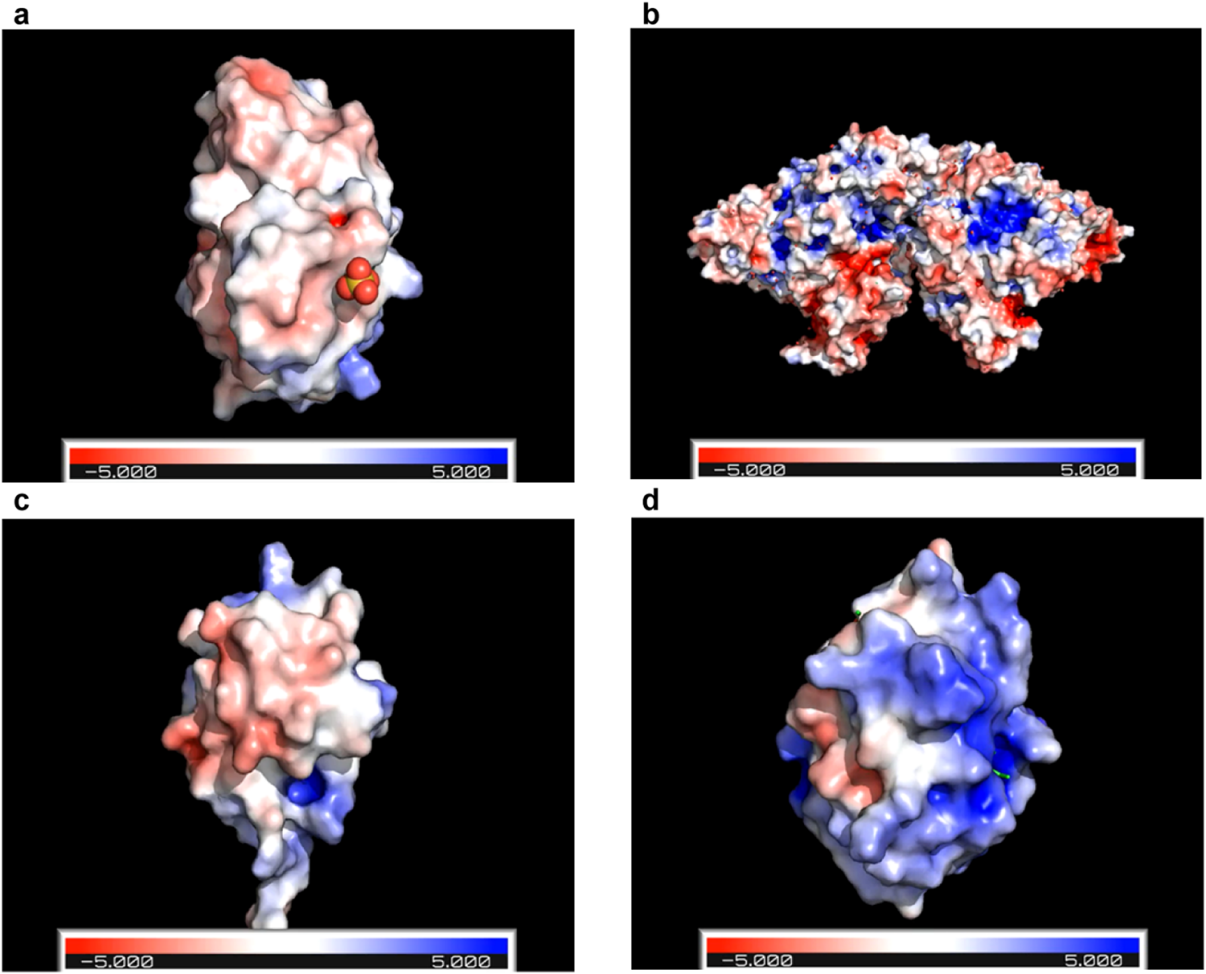
PyMOL computational images of the surface charge on the four proteins tested. **a** Myoglobin, **b** BSA, **c** ubiquitin and **d** cytochrome c. Positive surface charge is shown in blue, negative surface charge is shown in red. Protein computational images were produced using PyMOL v2.5 software and the Adaptive Poisson-Boltzmann Solver (APBS) electrostatic plugin.

The blank cotton face mask material did not result in a similar filter efficiency (in a range of ~26% difference) for the four proteins tested (Fig. 2). The same nebuliser (Supplementary Fig. S4) and air flow rates were used throughout the experiments; therefore, the particle speed is not likely to be a significant determining factor. Silica particles may exhibit more selectivity towards aerosolised proteins due to their unique physical properties, which differ from small molecules (Supplementary Table S2 and Fig. 3). Proteins have both hydrophobic and hydrophilic side chains, with capacity for electrostatic and hygroscopic interactions^57^. The polymeric proteins tested (e.g., cytochrome c, MW 12.4 k Da) also exhibit a larger conformation than smaller molecules, such as caffeine (MW 194 Da) and creatinine (MW 113 Da). These factors should increase the probability of silica-protein interactions^76^. The proteins aerosolised in this study also had different isoelectric points (pI, 4.8–9.6) and water contact angles. The electrostatic maps of surface charges for each of the four proteins in Fig. 3 show varying degrees of positive and negative surface charge distributions. These surface charges are attributed to basic amino acids carrying positive charges and acidic amino acids carrying negative charges at normal physiological pH^77^. Upon contact with the aerosolised proteins, the amide group of the QA ligands on the silica become positively charged, while unreacted areas of the silica have a net negative charge through silanol dissociation (Si-O-), further facilitating electrostatic interaction with charged regions of the proteins. Overall, all of these factors contribute to an increased likelihood of silica-protein interactions, facilitating protein-adsorption onto the silica surface.

### Filter efficiency of silica-coated cotton material for aerosolised inactivated SARS-CoV-2

Following on from the successful filtration of aerosolised proteins, we next assessed the filter efficiency of the silica-coated cotton for the primary target, aerosolised UV-inactivated SARS-CoV-2. This was carried out with the aid of a semi-quantitative lateral flow assay (LFA). The LFA test line intensity plateaus above approximately 7 × 10^4^ PFU/mL (Supplementary Fig. S6); to accommodate assay saturation and evaluate filtration efficiency in a biologically relevant range, all the before-filter sampling points were diluted (10% of initial concentration using the LFA extraction fluid prior to analysis, see materials and methods section for further details). To standardise the experiment, 1 mL of inactivated SARS-CoV-2 suspension was nebulised for each experiment.

**Fig. 4:**
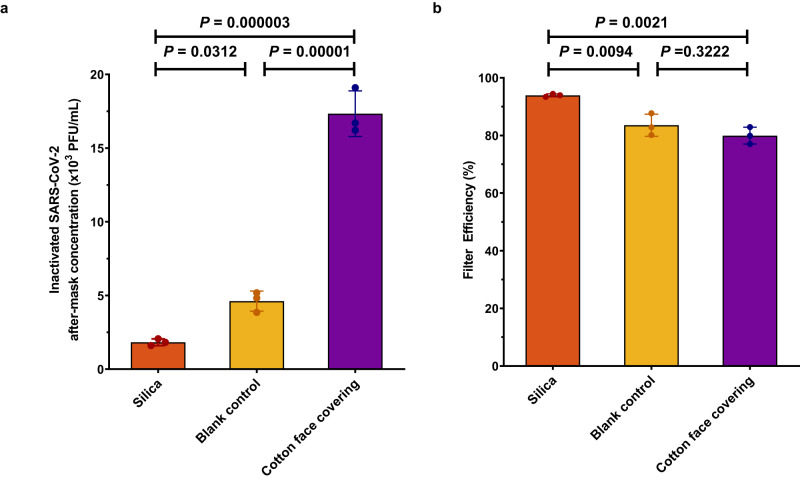
Filtration efficiency of aerosolised Inactivated SARS-CoV-2. **a** Inactivated SARS-CoV-2 after-mask concentrations, and, **b** corresponding filtration efficiencies of silica, blank control and the cotton face covering materials. All data are derived from three independent experiments (mean ± SD, *n* = 3). Comparisons were performed with one-way ANOVA (two-sided) with *Tukey HSD* post-hoc test. Each *p*-value was adjusted to account for Tukey’s multiple comparisons.

The addition of silica to the blank material provides an increased filter efficiency, decreasing the amount of SARS-CoV-2 that passed through the mask by 65% (Fig. 4a). Accounting for the concentration of inactivated SARS-CoV-2 which was recovered from the before-filter sampling point, the filter efficiency of the silica-coated face covering for aerosolised SARS-CoV-2 was the highest (average  ~94%), compared to ~84% for the blank material, and ~80% for the commercially available cotton face covering (Fig. 4b). This result is in accordance with our hypothesis and earlier filter efficiency results (Fig. 2) that the silica should have a propensity to adsorb SARS-CoV-2, which we surmise is due to the protruding spike proteins on its surface.

### Filter efficiency of silica-coated cotton material *vs* commercially available cotton face covering

To provide a basis for comparison, we also measured the filter efficiency of a common cotton face covering. The face covering is representative of commonly available cotton face coverings widely used by the general public. As summarised in Table 1, the filter efficiency of the silica-coated material significantly outperforms the common cotton face covering for the aerosolised proteins and aerosolised inactivated SARS-CoV-2 tested.

**Table 1:** Filtration Efficiency of face covering materials

Face covering material	Cytochrome c Filter Efficiency (%)	SARS-CoV-2 Filter Efficiency (%)	SARS-CoV-2 after-mask concentrations (x 10^3^ PFU/mL)
Silica-coated	95 ± 2	94 ± 0.4	1.6 ± 0.4
Blank Control	78 ± 5	84 ± 3	4.6 ± 0.6
Cotton Face Covering	48 ± 3	80 ± 2	14.9 ± 2.2

All data are derived from three independent experiments (mean ± SD, *n* = 3).

### Mechanistic considerations for silica-based capture/adsorption of aerosolised protein

Air filtration capture mechanisms can broadly be thought of as physical or electrical. Physical capture mechanisms take place without the influence of attractive forces (e.g., direct interception, inertial impaction, diffusional deposition, gravitational settling, etc.). Whereas electrical capture mechanisms involve electrical forces between the airborne particles and the filtration material. In reality, for any filter, an exact description of the filtration mechanism is difficult, because it is likely that several mechanisms will be acting together and to varying degrees.

In the case of the silica filter developed herein, it is clear that relative to a blank substrate (i.e., identical but without silica), the surface area available for physical capture has increased, which can increase the likelihood of physical interaction. In order to shed some light on the mechanism of capture by the silica and indeed our original hypothesis, we devised an experiment that uses only silica, without having to attach it to a substrate (Supplementary Fig. S8a). A key aim for this experiment is to be able to decipher the predominant mechanism that is responsible for the capture of aerosolised proteins by silica. Therefore, we tested two variants of the silica: i) the QA-functionalised silica that has been used for all other tests in this study which exhibits a markedly increased filter efficiency for aerosolised proteins, and ii) a heat-treated (800 °C) version of the same QA-functionalised silica. Performing a heat treatment effectively removes silanols from the surface (Supplementary Table S3), yielding silica particles that are essentially identical in terms of their physical size and shape, yet with distinctly different surface chemistries. As part of the experiment, aqueous protein solution is nebulised and passes through the silica particles held within a stainless-steel mesh support. Afterwards, protein is eluted from the silica prior to direct MS analysis. Further details of the test setup and analysis procedure are included in the methods section and Supplementary Fig. S8.

Our experiment revealed that the aerosolised protein capture efficiency of silica was about ten times higher than that of the heat-treated version (Supplementary Fig. S8b), clearly demonstrating that the predominant mechanism of interaction is not physical. Or at least one can say that the surface activity of the silica is vital, as in reality we expect that the increased surface area and surface chemistry work together in concert. This result agrees with our original hypothesis, that the surface chemistry of the mesoporous functionalised silica is crucial in capturing protein. Zeta potential measurements also corroborate this result showing a reduced surface charge of the heat-treated silica as expected (Supplementary Table S3).

Furthermore, this experiment sheds some light on the retention efficiency. We anticipate that the QA-functionalised silica can interact with polar groups present in proteins, such as amine and carboxylic acid groups, through hydrogen bonding and various types of electrostatic interactions. For this experiment, an organic solvent was used to elute (i.e., release) the protein from the silica for subsequent measurement. This indicates that bioaerosols captured by the silica are also likely to be retained by it. Finally, for comparison, we performed a similar experiment, except we exposed both silica variants by mixing them with protein in the liquid phase, which yielded a similar result (Supplementary Fig. S8c).

### ‘Breathability’ testing

**Fig. 5:**
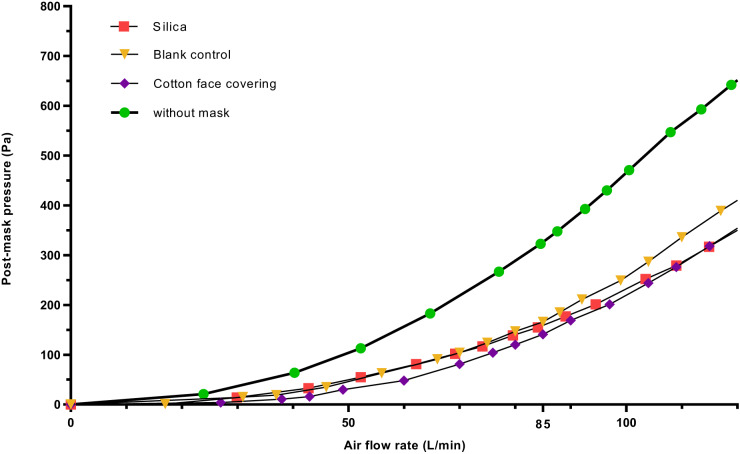
Curves of post-mask measured air pressure against air flow rate using different materials. All data shown are representative of at least three independent experiments.

The pressure drop measured across each mask material gives an estimate of the relative breathability of each face covering. A lower pressure drop means increased breathability of the material and thus comfort. The breathability test results for the three mask materials are summarised in Fig. 5 and Table 2. The pressure change curve at increasing air flow rates using silica-coated cotton overlapped well with those of the blank control and cotton face covering (Fig. 5). The average pressure drop at a constant air flow rate of 85 L/min for the silica-coated cotton, blank control and cotton face covering were broadly similar (152, 143 and 166 Pa, respectively, Table 2). This indicates that the addition of mesoporous silica particles to the cotton substrate made only a slight increase (by 6.2%) to the subsequent pressure drop but was still lower than the commercially available cotton face covering by 8.4%. Interestingly, this suggests that a silica coating has the potential to be used in combination with other materials (including established face masks) to improve filter efficiency yet with nearly no impact on breathability/pressure drop.

**Table 2:** Breathability test result (pressure drops) and filter quality factor (QF)

Face Covering Material	Pressure drop compared to the unobstructed response at an air flow rate of 85 L/min (Pa)	QF (kPa ^-1^)
Silica	152 (22 Pa/cm^2^)	8.6
Blank Control	143 (20 Pa/cm^2^)	4.6
Cotton Face Covering	166 (24 Pa/cm^2^)	1.7

All data shown are representative of at least three independent experiments. The QF was calculated using Eq. 2 with the mean filter efficiency of cytochrome c.

### Filter quality factor

The filter quality factor (QF) is a relative indicator to assess the overall performance of an air filter (see Eq. 2, Materials and Methods). For testing at similar flow rates, a higher QF value indicates better overall efficiency, which is a result of both higher filtration efficiency and lower pressure drop. According to expert consensus reported in WHO guidance^78^, a QF ≥ 3 is the minimum value recommended for cloth masks. In comparison, the silica-coated substrate had an almost two-fold increase in QF compared to the blank control (8.6 *vs* 4.6 kPa^−1^, Table 2). The QF for the commercial cotton face covering was below the WHO recommended level (1.7 kPa^1^), suggesting that it may not provide sufficient filtration efficiency.

In conclusion, an innovative concept in face coverings has been demonstrated that utilises functionalised mesoporous silica for the adsorption of bioaerosols. In this feasibility study, the well-known affinity of silica for some common proteins has been demonstrated in an aerosolised form showing filter efficiencies of 97%, 87%, 91 % and 95% for aerosolised myoglobin, BSA, ubiquitin and cytochrome c, respectively – with an average increase in filter efficiency of ~17% compared to an untreated blank. The addition of amorphous silica to a swab was also shown to decrease the amount of aerosolised SARS CoV-2 that passed through the face covering by ~65%. The silica-coated substrate had nearly no impact on the pressure drop across the face covering. This approach enables increased filter efficiency performance whilst retaining the advantages of a ‘breathable’ substrate.

There are several exciting possibilities for future work to explore and extend the proof-of-concept research demonstrated herein. Preliminary tests indicated that the silica-coated swabs remain useable for at least one month. Subsequent studies are warranted to consider longer-term durability more widely, including simulation of routine usage scenarios. Further experimentation and theoretical analysis should explore silica-bioaerosol interactions, within the context of air filtration, in greater detail. Cotton was mainly used as the substrate material in this work, and this can be extended further by considering multi-layer coverings as well as alternative substrate materials. Silica particles were functionalised with QA ligands and future work will consider the anti-viral properties, if any, provided by the QA-functionalised silica; previous research on silica nanoparticles with cationic QAs found they demonstrate antifungal, antibacterial and antimicrobial behaviour^79^. Moreover, the tunability of silica offers a plethora of further opportunities to bind to other analytes with improved selectivity, by changing the particle size, pore size and functional groups. While this paper has focused on common face coverings, the utilisation of silica as a virion adsorbent shows excellent potential for use in face coverings, air filtration systems and even as a bioaerosol sampler.

## Methods

### Chemicals and reagents

Cytochrome c from bovine heart ( ≥ 98%), myoglobin from equine skeletal muscle ( ≥ 95%), ubiquitin from bovine erythrocytes ( ≥ 98%), BSA ( ≥ 98%), caffeine ( ≥ 99%), creatinine (purity ≥ 98%), formic acid (reagent grade, ≥ 95%), tetraethyl orthosilicate (TEOS) ( ≥ 99.99%), ammonium hydroxide solution (puriss p.a., ≥ 25% NH3 basis) and dodecylamine (reagent grade, 98%) were purchased from Sigma–Aldrich (St. Louis, Mo, USA). N-Trimethoxysilylpropyl-N,N,N-trimethylammonium chloride (TMAPS) (50% in methanol) was obtained from Gelest (Morrisville, PA, USA). Methanol (HPLC grade purity ≥ 99%) was purchased from VWR Chemicals (Radnor, PA, USA). Toluene (reagent grade) and isopropanol (HPLC grade) were bought from Fisher Scientific. Water was purified using a Milli-Q Advantage A10 water purification system (Millipore, MA, USA).

### Inactivated SARS CoV-2

Vero E6 cells (isolated from African green monkey kidney cells) (VERO C1008, American Type Culture Collection, Manassas, VA, USA) were obtained from Public Health England (The UK Health Security Agency) and maintained in Dulbecco’s minimal essential medium (DMEM) without antibiotics or serum at 37 °C with 5% CO_2_. SARS-CoV-2 isolate SARS-CoV-2/human/Liverpool/REMRQ0001/2020 was cultured from a patient’s nasopharyngeal swab. It was passaged a further four times in the Vero E6 cells. The 4th passage of the virus was cultured in the Vero E6 cells with DMEM at 37 °C with 5% CO_2_. The virus was harvested 48 h post-inoculation and stored at −80 °C before use.

The virus was inactivated through the application of UV energy > 0.04 J/cm^2^ ^80^. Viral titers were determined by plaque assay prior to inactivation (1.3 × 10^7^ PFU/mL). The inactivated virus was stored at −80 °C until required.

### Preparation of aerosol stock and working solution

1 mg of each standard material (cytochrome c, myoglobin, BSA, ubiquitin, caffeine and creatinine) was weighed and dissolved in 10 mL water separately to make a standard working solution at 1 mg/mL. It was directly used for nebulisation without further dilution.

For inactivated SARS CoV-2, a stock buffer solution of DMEM containing 1.3 × 10^7^ PFU/mL of inactivated SARS CoV-2 was diluted 10 times with water (1: 9, v/v) before use. The working solution was approximately at 1.3 × 10^6^ PFU/mL concentration.

Each working solution was freshly prepared each day before analysis.

### Silica-coated cotton face covering

#### Synthesis of amorphous silica and QA functionalisation

QA-functionalised amorphous mesoporous spherical silica was synthesised using a process based on the method of Stober et al.^81^. The spherical silica particles were prepared by hydrolysing and condensing tetraethyl orthosilicate (TEOS) with long-chain n-alkylamines in aqueous ammonium hydroxide solution at pH 10, with isopropanol as the co-solvent. Supplementary Fig. S7 provides a schematic diagram illustrating the key steps involved in the synthesis of QA-functionalised silica.

In a typical synthesis procedure, a solution of dodecylamine in a mixed solution of isopropanol (0.16 L) and water (0.1 L) was prepared. Tetraethyl orthosilicate (TEOS) was then slowly added dropwise to the solution at different temperatures with magnetic stirring. As TEOS was added, the clear solution gradually became opaque due to the formation of a white precipitate. The mixture was continuously stirred for 4 h, after which the white precipitate was filtered and repeatedly washed with water and ethanol four times. The resulting product was then dried in a vacuum oven at 80 °C for 4 h.

The reaction is initiated with a homogeneous solution at room temperature or higher, and the average particle diameter of the precipitated particles can be controlled by adjusting the water-TEOS molar ratio in the starting solution, allowing for the production of nearly monodisperse particles with a narrow size distribution.

Surface derivatisation of the silica gel with a QA ligand was prepared with N-trimethoxysilylpropyl-N,N,N-trimethylammonium chloride (TMAPS, a kind of QA silane). Typically, a 10 g aliquot of previously synthesised silica gel was dried in a vacuum oven at 150 °C for 16 h. The dried silica gel was then suspended in 100 mL of dry toluene in a 3-neck round-bottom flask connected to a reflux condenser. This mixture was heated to 40 °C with slow stirring to form a slurry, and then 15 mL of TMAPS was added. The reaction mixture was stirred for 6 h at 111 °C to activate the QA ligand on the silica surface. Afterwards, the activated silica particles were washed successively with methanol and dried at 80 °C for 16 h.

#### Substrate materials

Cotton is commonly used as a material for face masks and coverings^17,82^ due to its availability, comfort, breathability, and moderate filtration performance^19,82^. Two non-woven 4-layer swab variants of size 5 × 5 cm, made of cotton, were purchased online from JFA medical Ltd in the UK. Cotton swabs were used as a substrate for filter efficiency tests, with silica and without (blank control).

#### Bonding silica spheres to a cotton substrate

The cotton swabs were placed into a shallow glass dish with 1 L of 10% methanol in warm water. 1 g silica, with QA ligands, per cotton swab (5 × 5 cm), was added and allowed to sit for 3 h, with periodic agitation. The swabs were dried in the oven for at least 30 min at 150 °C to remove all the water completely (Supplementary Fig. S7b).

#### Silica filters for face coverings

Quality control was performed by randomly selecting a dried swab and viewing it under a microscope, further analysis was done using Scanning Electron Microscopy (SEM). The silica-cotton swabs were put inside two pieces of fusible interfacing material, cut to size, that were pressed with an iron on a low heat setting to seal shut the swab inside. This was a precaution taken in case there was excess silica or weakly bound silica that might be extracted from the mask under high flow rate; upon examination, even after extensive testing at high air flow rates, the silica remained bound to the cotton substrate. Conceivably, without the gauze, any weakly bound silica could be drawn into the fan, which in a real case scenario is representative of the human respiratory system. No functionalised silica was ever recovered from outside the swab. This research did not involve any human participants, and all experiments were performed in a fumehood. The silica used was amorphous.

### Commercially available cotton face covering

A commercially available cotton face covering was chosen because it is readily available and freely distributed within the University of Liverpool. The cotton face covering is made of cloth (100% cotton) with 2-layers and supplied by Earth Squared, UK, which is commercially available to the public.

### Filter efficiency test

**Fig. 6:**
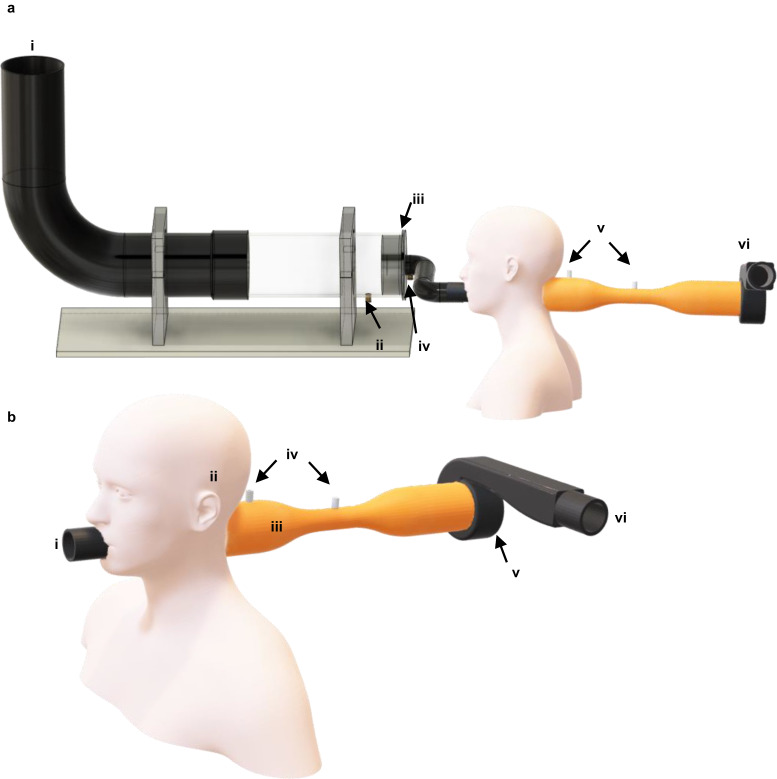
CAD images of the experiment apparatus used for filter efficiency and breathability testing. **a** CAD image of the test rig design for the filter material penetration test. **i** Nebuliser input and entry for clean air to be drawn in. **ii** Valve to connect to a pre-mask syringe filter to yield a before-mask concentration (BMC). **iii** Mask material clamped in position. **iv** Valve to connect to a post-mask syringe filter to yield an after-mask concentration (AMC). **v** Pressure sensors. **vi** Fan and exhaust. **b** CAD image of the test rig design for filter material differential pressure (‘breathability’) tests. **i** Entry for clean air in (‘mouth’). **ii** ‘Ears’ used to attach masks. **iii** Venturi tube. **iv** Pressure Sensors. **v** Fan. **vi** Exhaust (air out).

**Fig. 7:**
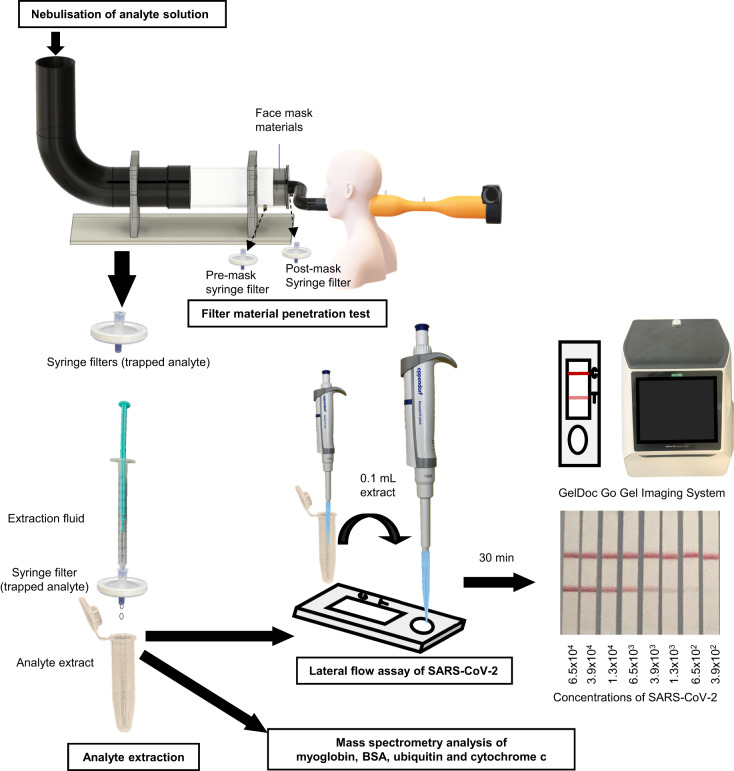
Overview of the main steps involved in the filter efficiency tests for aerosolised proteins, small molecules and inactivated SARS-CoV-2.

The apparatus and workflow are shown in Figs. 6a, 7, respectively. Briefly, the test setup draws in air along with the nebuliser output at a given flow rate which passes through the material under test (i.e., the filter material), which is clamped in position (Fig. 6a). A portion of the air is sampled both before and after the material under test, and subsequently measured to determine the filter efficiency. Two measurement methods were carried out: mass spectrometry for the four common proteins and lateral flow assay for inactivated SARS-CoV-2 (Fig. 7). The filtration efficiency for common proteins was tested using the silica coated face covering and a non-coated blank cotton substrate (acting as a blank control). In addition to the two types of materials, the test for inactivated SARS-CoV-2 also included the commercially available cotton face covering mask; this is a typical and widely deployed cotton-based face covering providing a relative comparison. It is important to note that the key aim at this stage is to evaluate the silica face covering concept with regards to the hypothesised improved filter efficiency, since it is conceivable in the future that other established masks and air filters could incorporate silica into future designs.

#### Setup for filter material penetration

Bioaerosol concentrations can usually be determined by 6 main methods; sedimentation, filtration, centrifugation, impaction, impingement and microfluidics^83^. Filtration is the method used herein, due to the ease of operation. The airflow experiment was conducted using a test rig design (Fig. 6a) that was adapted from the specification published by Delft University of Technology (TU Delft, Netherlands)^84,85^. More information about the test rig can be found in the supplementary information. All filter efficiency and breathability tests were carried out with the test apparatus mounted inside a fumehood.

#### Bioaerosol generation method

Various analytes were aerosolised as part of the filtration efficiency testing. This included generic proteins: cytochrome c, myoglobin, ubiquitin and bovine serum albumin (BSA). Further analytes nebulised included inactivated SARS CoV-2, caffeine and creatinine.

The test solutions were nebulised by an Ortorex portable ultrasonic nebuliser. The emitted aerosol particle size distributions were characterised using a handheld 6-channel dust particle counter using a laser diffraction system (TC-8200, Dongguan Huazhong Instrument Co., Guangdong, China) (Supplementary Fig. S4). During experiments, the test solutions were nebulised at a flow rate of 0.7 mL/min. Before each experiment, the nebuliser was flushed with water to remove any carry over. Approximately 2 mL of the protein working solution (cytochrome c, myoglobin, ubiquitin and BSA, 1 mg/mL) or 1 mL of inactivated SARS-CoV-2 working solution (1.3 × 10^6^ PFU/mL) was added into the nebuliser separately and nebulised for 3 min as part of the filtration efficiency testing. A test to compare bioaerosol capture versus small molecules is discussed in the supplementary information (Supplementary Fig. S5), in which the analytes caffeine (MW 194 Da) and creatinine (MW 113 Da) were similarly dissolved in 10 mL of water to 1 mg/mL.

#### Filter material penetration test

Cytochrome c (MW ~12.4 kDa) was dissolved in 10 mL of water to 1 mg/mL. Myoglobin (MW ~17.6 kDa), BSA (MW ~66.5 kDa) and ubiquitin (MW ~8.6 kDa) were each prepared in a similar manner. Approximately 2 mL of the 1 mg/mL solution was nebulised for 3 min.

A subset sample of the airflow was collected before (Fig. 6a, ii) and after (Fig. 6a, iv) the mask filter (Fig. 6a, iii), which had a diameter of 4 cm, to allow for determination of the percentage drop in particulates caused by the mask. A sample of the air immediately before the mask was drawn across a PTFE syringe filter connected to a backing pump at a flow rate of 10 mL/min. Similarly, an identical syringe filter was connected to a line sampling the air flow immediately after the face covering material, connected to the same backing pump (flow rate, 10 ml/min). The syringe filters (PTFE, 0.22 µm pores) were purchased from Restek (PA, USA). Further information regarding the extraction and detection techniques for the analytes tested is given below in detection method of bioaerosol. In between individual tests, the filter efficiency apparatus was cleaned with a microfibre cloth and subjected to a short period (approximately 10 min) of a constant air flow to remove any residual carryover. The nebuliser was also cleaned and a blank consisting of 5 ml of water was nebulised to ensure that no carryover was observed.

#### Protein extraction and MS analysis for common proteins

For a typical filtration efficiency test, the nebulised protein (cytochrome C, myoglobin, ubiquitin and BSA) was first captured on a syringe filter (PTFE 0.22 µm, 30 mm) at the upstream (Fig. 6a, ii) and downstream (Fig. 6a, iv) sampling points close to the locations that aerosol engages with the filter material under test. Afterwards, the analyte trapped by each syringe filter was recovered with 2 mL 50% methanol in water with 1% formic acid (an additive to aid protonation). A large syringe (50 mL) was used to push air through to recover the solvent and analyte as much as possible. The extract was analysed by directly injecting into the mass spectrometer for MS analysis. A preliminary blank analysis was conducted before each sample to ensure that no carryover in the mass spectrometer was observed between experimental runs.

The MS analysis was carried out on a Waters QDa mass spectrometer using electrospray ionisation (ESI) in positive ion mode. The ion source temperature was maintained at 65 °C. The flow rate was set to 250 µL/min. The cone voltage was set to 35 V and capillary voltage 1.5 kV. Quantitation was performed by monitoring the average peak intensities of the most abundant ion of the analyte within a 1 min scan (cytochrome c: *m*/*z* 875; myoglobin: *m*/*z* 893; ubiquitin: *m*/*z* 857; BSA: *m*/*z* 1234). The MS instrument was controlled using Waters MassLynx software (version 4.1, MA, USA).

#### Silica capture mechanism experiments

In order to assess the mechanism by which silica captures aerosolised protein and also to explore its retention efficiency, we developed a fibreless nebulising system (Supplementary Fig. S8a). The main part of the chamber was fabricated using photoresin with a 3D printer (Formlabs, UK). This section incorporates a stainless-steel mesh support and an exhaust directly below it. A polycarbonate enclosure (RS components, UK) was modified to connect to the 3D printed part at one end and the nebuliser at the other, to remove any larger droplets emitted from the nebuliser. Aerosolised protein that passes in to the 3D printed chamber is able to escape via an exhaust mounted in the base, and is actively sampled by a vacuum pump (flow restricted to 10 L/min) connected at the top of the chamber. A removable piece contains two fixed woven stainless steel meshes and another woven stainless-steel mesh that is removable (MeshDirect, UK). The central mesh (mesh 28) has an aperture of 0.55 mm. This is used to provide an even spacing for the silica when it held in place for testing. Below this is a fixed 0.055 mm aperture (mesh 300), and above is another identical mesh which is detachable. The silica is applied evenly with the detachable mesh removed. After application it can be placed on top and is held in place by four screws. This entire piece can be removed allowing it to be readily washed, weighed and also dried in an oven post-nebulisation (and prior to removing exposed silica, which is best removed when dry). The nebuliser used for this particular experiment was a compressor type Omron C28P (Omron Healthcare, UK).

Silica was tested in two variants: (i) in its regular form as utilised in all other tests in this study, and, (ii) heat-treated (800 °C) to remove silanols from the surface. Both were tested using identical conditions.

The capture mechanism experiment exposed 0.2 g of each silica variant, held within a stainless-steel mesh, to nebulised BSA, 1 mg/mL dissolved in water. During the experiment, the protein solution was nebulised at a flow rate of 0.4 mL/min for 5 min, afterwards the silica was sufficiently dried in an oven and then collected from the mesh support. Both silica samples were then washed three times with water using vigorous vortexing, prior to elution of the protein from the silica using 100% methanol. 200 µL resulting extract was then mixed with 192 µL water, 4 µL 0.1 mg/mL cytochrome c (final concentration = 1 µg/mL) and 4 µL formic acid (final concentration = 1%), prior to direct injection into the mass spectrometer for analysis.

In relation to retention efficiency, acting as a control of sorts, each silica was incubated with protein in solution phase. The protein used for this test was also BSA at a concentration of 0.01 mg/mL dissolved in water, which was incubated with 0.05 g of each silica for 1 h before MS analysis.

#### Extraction method, lateral flow assay and imaging for inactivated SARS-CoV-2

Inactivated SARS-CoV-2 (0.1 mL, 1.3 × 10^7^ PFU/mL) was diluted to 1 mL in water. The filter efficiency test was repeated (*n* = 3) following the procedure as described above. However, the syringe filters were 13 mm instead of 30 mm. In a similar fashion, these were used to collect aerosolised material (in this case inactivated SARS-CoV-2) and a pre-prepared commercial extraction fluid (0.4 mL) was used as provided with a FlowFlex SARS CoV-2 Antigen Rapid Test (lateral flow assay kit). The lateral flow assays used were from the same LOT and factory number, to minimise variability. 0.1 mL of the extract was recovered through the syringe filters and dripped onto the SARS-CoV-2 antigen rapid test strip. It was then allowed to rest for 30 min as per the manufacturer’s instructions (Fig. 7). The test strips were analysed using a GelDoc Go Gel Imaging System (BioRad, CA, USA) and the intensity of the control and test strip lines were recorded and analysed using Image Lab software (version 3.0.0.07, BioRad, CA, USA). A calibration curve of 13 different concentrations of SARS-CoV-2 between 1.3 × 10^2^ and 6.5 × 10^5^ PFU/mL was produced to allow for conversion of test line intensity to concentration (Supplementary Fig. S6).

#### Filtration efficiency calculation

Extraction fluids before and after syringe filter (see Fig. 6a, ii, iv) were used for MS (proteins) and lateral flow assay (SARS-CoV-2) analysis to determine the percentage that had passed through the mask. The filter efficiency was calculated using Eq. 1:

1 $${Filter\; Efficiency}\,(\%)=\frac{{BMC}-{AMC}}{{BMC}}\times 100$$

where BMC and AMC are upstream pre-mask concentration (before mask) and downstream post-mask concentration (after mask), respectively. A higher filter efficiency indicates the material is better at preventing transmission of the respective nebulised target analyte.

### Breathability (or pressure drop) test

The pressure drop across each material was measured using a pressure sensor (Sensirion, SDP816-500PA), placed behind the covering (at approximately 10 cm) relative to atmospheric pressure (Fig. 6b). The pressure was recorded at increasing fan speeds and plotted against the air flow rate. A breathability pressure drop of each face covering material is the differential pressure relative to the pressure when there is no obstruction (i.e., an open hole at Fig. 6b, i), measured at 85 L/min constant air flow, which is the standard flow rate used by the United States National Institute for Occupational Safety & Health (NIOSH)^86^. The breathability of the mask is also dependent on the seal the mask forms with the face; every effort was taken to provide the most natural fit of the mask to the mannequin head.

### Filter quality factor calculation

The filter quality factor (QF) is a function of filtration efficiency and breathability^26^ and calculated as follows:

2 $${QF}=-\frac{{{\log }}(1-{FE}/100)}{\Delta P}$$

where FE is the filter efficiency (%) and Δ*P* is the pressure drop (kPa).

### Graphs and statistical analyses

All graphs were drawn using GraphPad Prism 8 software. The same software was used for statistical analyses. Statistical differences were evaluated either by two-tailed Student’s *t*-test or one-way ANOVA (two-sided) with *Tukey HSD* post-hoc test at a univariate level. Each *p* value was adjusted to account for Tukey’s multiple comparisons. A *p*-value < 0.05 was considered to be statistically significant. Data are expressed as mean ± standard deviation (SD). All experiments were repeated three times independently, unless otherwise noted.

### Reporting summary

Further information on research design is available in the Nature Portfolio Reporting Summary linked to this article.

### Supplementary information

Supplementary Information

Reporting Summary

Peer Review

## Data Availability

The data generated or analysed during this study are presented in the published article and corresponding supplementary information files. All other data are available from the corresponding author upon request.
