# Supplementary material for: Attaching protein-adsorbing silica particles to the surface of cotton substrates for bioaerosol capture including SARS-CoV-2
Source: Nat Commun. 2023 Aug 18;14:5033. doi: 10.1038/s41467-023-40696-x (PMC10439164; doi:10.1038/s41467-023-40696-x)
Supplement: Supplementary file 1 — Supplementary Information [file 41467_2023_40696_MOESM1_ESM.pdf]

# Supplementary Information

## Attaching Protein-Adsorbing Silica Particles to the Surface of Cotton Substrates for Bioaerosol Capture including SARS-CoV-2

Kieran Collings[1], Cedric Boisdon[1], Tung-Ting Sham[1], Kevin Skinley[2], Hyun-Kyung Oh[1], Tessa Prince[3], Adham Ahmed[2], Shaun H. Pennington[4], Philip J. Brownridge[5], Thomas Edwards[4], Giancarlo A. Biagini[4], Claire E. Eyers[5], Amanda Lamb[6], Peter Myers[2,+] and Simon Maher[1,*]

[1]Department of Electrical Engineering and Electronics, University of Liverpool, Liverpool, UK.

[2]Department of Chemistry, University of Liverpool, Liverpool, UK.

[3]Institute of Infection, Veterinary and Ecological Sciences, University of Liverpool, Liverpool, UK.

[4]Centre for Drugs and Diagnostics, Department of Tropical Disease Biology, Liverpool School of Tropical Medicine, Liverpool, UK.

[5]Centre for Proteome Research, Department of Biochemistry & Systems Biology, Institute of Systems, Molecular & Integrative Biology, University of Liverpool, Liverpool, UK.

[6]Faculty of Health and Life Sciences, University of Liverpool, Liverpool, UK.

Correspondence and requests for additional material should be addressed to:
[+] peterm@liverpool.ac.uk, [*]s.maher@liverpool.ac.uk

# Table of Contents

Supplementary Methods ........................................ 5

**Water contact angle** ........................................ 5

**Test rig design and specification** ........................................ 5

**Surface area and porosity of silica particle by nitrogen adsorption** ........................................ 5

**Zeta potential measurement** ........................................ 5

Supplementary Tables ........................................ 6

**Supplementary Table S1.** Particle size distribution of silica particles by Malvern Mastersizer 3000 (Malvern, UK). Mass median diameter (median of volume distribution) = 51.4 ± 0.02 µm. All data are derived from five independent experiments (mean ± SD, *n* = 5). ........................................ 6

**Supplementary Table S2.** Molecular weight, diameter, isoelectric point, and water contact angle of the proteins. All data of water contact angle are derived from three independent experiments (mean ± SD, *n* = 3). ........................................ 6

**Supplementary Table S3.** Effect of heat treatment on 50 µm silica and the presence of QA ligands by zeta potential measurements. All data are derived from three independent experiments (mean ± SD, n = 3). ........................................ 7

**Supplementary Table S4.** Refractive index of aqueous solutions of: pure water, proteins (1 mg/mL in water), buffer solution (inactivated SARS-CoV-2), small molecules (1 mg/mL in water) and ethanol (used as a positive control). All data shown are representative of at least three independent experiments. ........................................ 8

Supplementary Figures ........................................ 9

**Supplementary Figure S1.** Particle size distribution of silica particles by Malvern Mastersizer 3000 (Malvern, UK). Mass median diameter (median of volume distribution) = 51.4 ± 0.02 µm. All data are derived from five independent experiments (mean ± SD, *n* = 5). ........................................ 9

**Supplementary Figure S2. a**. Measurement of pore volume and pore size of non-QA bonded bare silica. The mean pore volume was 0.96 $cm^3$ /g and mean pore diameter (radius × 2) was 11.48 nm. **b**. The Brunauer–Emmett–Teller (BET) isotherm displays a Type IV H2 hysteresis. This type is associated with capillary condensation in mesopores. ........................................ 10

**Supplementary Figure S3.** Storage stability of silica-coated swabs in relation to their filter efficiency for aerosolised proteins. **a**. myoglobin, and, **b**. ubiquitin. The silica-coated cotton materials were stored under ambient conditions for one month before undertaking a filter efficiency test and compared against freshly prepared silica swabs. All data are derived from three independent experiments (mean ± SD, *n* = 3). Comparisons were performed with two-tailed Student's *t*-test. ........................................ 11

**Supplementary Figure S4.** Particle size distribution of aqueous aerosol containing 0.1 mg/mL of **a**, pure water; **b**, cytochrome c; **c**, BSA; **d**, ubiquitin; **e**, myoglobin; **f**, inactivated SARS-CoV-2 in buffer solution; **g**, creatinine; **h**, caffeine. They were generated with a nebuliser (ORTOREX™ Portable Ultrasonic Nebuliser) and measured with a handheld 6-channel dust particle counter which uses a laser diffraction system (TC-8200, Dongguan Huazhong Instrument Co., Guangdong, China). The most frequent band of particle diameter lies at ~0.3 µm in each case. The concentration of the

inactivated SARS-CoV-2 in buffer solution used here was 1.3 x $10^5$ PFU/mL, which was diluted 10 times from the original solution due to instrumental signal saturation. All data are derived from three independent experiments (mean ± SD, *n* = 3). ............................12

**Supplementary Figure S5.** Filter efficiency of small molecules (creatinine and caffeine) and cytochrome c for the silica coated material and blank material. All data are derived from three independent experiments (mean ± SD, *n* = 3). Comparisons were performed with two-tailed Student's *t*-test. A solution containing 1 mg/mL of all three analytes, creatinine, caffeine, and cytochrome c, dissolved in water was prepared. Caffeine was chosen as a slightly hydrophobic, low molecular-weight, polar analyte with moderate water solubility under agitation. Creatinine was selected as a low molecular-weight, polar, hydrophilic analyte that is water soluble. The silica swabs saw an increase in filter efficiency for creatinine and cytochrome c, indicating that the silica can enhance the filter efficiency of hydrophilic aerosols, especially proteins. Caffeine showed high filter efficiency even for the uncoated blank control, but the filter efficiency of the silica material decreased slightly, which was not statistically significant. The increase in filter efficiency for cytochrome c was significantly greater than that of creatinine, suggesting that the functionalised silica has a stronger affinity for aerosolised proteins, as anticipated. Furthermore, only cytochrome c showed a statistically significant ($p<0.05$) difference in filter efficiencies between the silica-coated and uncoated swabs...........................................................................................13

**Supplementary Figure S6.** Calibration curve of lateral flow assay, using GelDoc UV to quantify the intensity of the positive test line. Experimental parameters were controlled to produce post-mask syringe filters concentration (AMC) between 1.3x$10^3$ and 1x$10^5$ PFU/ml. Before-mask syringe filters (BMC) were diluted to a concentration of 1/$10^{th}$ to match those parameters and prevent the concentration exceeding 7x$10^4$ PFU/ml and thus being incalculable due to saturation. All data are derived from three independent experiments (mean ± SD, n = 3). .........................................................................14

**Supplementary Figure S7.** Schematic representation of QA-functionalised amorphous silica synthesis. **a**, Functionalisation of silica gel with N-trimethoxysilylpropyl-N,N,N-trimethylammonium chloride (TMAPS, a kind of quaternary amine (QA) silane) by stirring the reaction mixture in toluene at 111 $^oC$ for 6 hr. **b**, Condensation reaction of silanol groups on the surface of QA-functionalised silica to hydroxyl groups of cellulose under heating...........................................................................................15

**Supplementary Figure S8.** The capture efficiency of functionalised silica was tested in a fibreless setup (without attaching silica to a fabric substrate) to shed light on the underpinning mechanism responsible for aerosolised protein capture. **a**. Schematic illustration of the fibreless test apparatus. The triple mesh is made from woven stainless steel and holds the unbound silica particles in place. Mesh 300 is a woven stainless-steel mesh with an aperture of 0.055 mm, and mesh 28 is a woven stainless-steel mesh with an aperture of 0.55 mm; refer to Materials and Methods for further details. Silica is held in the mesh support and is exposed to nebulised bovine serum albumin (BSA). **b**. Peak area ratios of BSA to cytochrome c, in which BSA was eluted from the two silica variants after exposure to aerosolised BSA (1 mg/mL in water), and then mixed with cytochrome c prior to MS analysis. **c**. Peak area ratios of BSA to cytochrome c, in which BSA was eluted from the two silica variants after being exposed to BSA (0.01 mg/mL) in solution phase, and then mixed with cytochrome c prior to MS analysis. Details of the experimental method are given in the methods section. All data are derived from three independent experiments (mean ± SD, n = 3). Comparisons were performed with two-tailed Student's *t*-test...........................................................................................16

Supplementary References........................................................................................17

# Supplementary Methods

## Water contact angle

To improve understanding of the different characteristics of the proteins used in testing the filter efficiency, the water contact angle was measured using a drop shape analyser (Krüss-DSA100). The proteins were coated onto a glass slide by pipetting the dissolved solutions (in water) onto the surface and evaporating in an oven (65ºC). A hydrophilic surface will have a water contact angle less than 90º, a hydrophobic surface will have a water contact angle greater than 90º. An extremely hydrophilic surface will completely wet across the surface, making the angle unmeasurable. The plain glass slide had a water contact angle of approximately 10º.

## Test rig design and specification

The testing rig used in all aerosol filter efficiency tests was made primarily from PVC pipes and plexiglass tubes. The horizontal section (as shown in Figure 6a, main manuscript) is approximately 60 cm long with an internal diameter of approximately 10 cm, the PVC 90º bend features a vertical section that is approximately 30 cm, to give an approximate total length of 90 cm. Depending on the joint, either a rubber seal, PVC tape or silicone sealant were used to guarantee an airtight seal. The fan used to generate the air flow was a SanyoDenki San Ace B97; a 24 V fan with a diameter of 97 mm. The differential pressure sensor used was a Sensirion SP800-analog, with a maximum pressure differential of 500 Pa. The internal area of the test rig is approximately 8000 $cm^3$.

## Surface area and porosity of silica particle by nitrogen adsorption

To determine the surface area, pore volume, and pore size of the bare silica particles prior to QA functionalisation, we performed nitrogen adsorption−desorption measurements using a Micromeritics 3-Flex instrument (Norcross, GA, USA). The Brunauer−Emmett−Teller (BET) surface area was calculated by applying the BET equation to the adsorption data [1], while the pore size distribution was determined using the Barrett− Joyner−Halenda (BJH) method [2], applied to the adsorption branch of the sorption isotherms.

All samples were degassed at 150 °C for 3 h before measurement. The specific surface area (6 points, $0.025 < P/P_0 < 0.30$) and pore size distribution were calculated using the multi-point BET equation and BJH model, respectively. The total pore volume was determined at the last point of the adsorption branch, corresponding to a relative pressure of 0.99.

## Zeta potential measurement

Zeta potential of four types of silica samples were tested (Supplementary Table S2): 1), silica gel control without any treatment; 2), silica gel heated at 800°C in a furnace for 2 days; 3), silica gel functionalised with QA ligand; 4), silica gel heated at 800°C in a furnace for 2 days and then functionalised with QA ligand.

0.1 g of each silica sample was suspended in 10 mL of water and dispersed using an ultrasonic bath for 10 min. Zeta potential measurement was carried out immediately using Zetasizer nano ZS (Malvern Panalytical Instruments, UK) after removing the suspension from the bath. Before the measurement, the stability of the suspension was automatically tested by the Zetasizer nano ZS. Each sample was measured in triplicate with a minimum of 10 sub runs to determine analysis reproducibility.

## Supplementary Tables

**Supplementary Table S1.** Particle size distribution of silica particles by Malvern Mastersizer 3000 (Malvern, UK). Mass median diameter (median of volume distribution) = 51.4 ± 0.02 µm. All data are derived from five independent experiments (mean ± SD, *n* = 5).

| | **Percentile** | | |
|---|---|---|---|
| **Volume distribution** | **10 %** | **50 %** | **90%** |
| **Particle diameter below (µm)** | 35.3 ± 0.02 | 51.4 ± 0.02 | 74.1 ± 0.04 |

**Supplementary Table S2.** Molecular weight, diameter, isoelectric point, and water contact angle of the proteins. All data of water contact angle are derived from three independent experiments (mean ± SD, *n* = 3).

| **Protein** | **Molecular Weight (kDa)** | **Diameter (nm)** | **pI** | **Water Contact Angle (º)** |
|---|---|---|---|---|
| **Myoglobin** | 17.6 | 3.5 [3] | 7.4 [4] | 25.1 ± 1.2 |
| **Cytochrome c** | 12.4 | 3.1 [3] | 9.6 [4] | 16.0 ± 1.3 |
| **Ubiquitin** | 8.6 | 2.5 [5] | 6.7 [6] | Spreading |
| **BSA** | 66.7 | 3.5 [7] | 4.8 [8] | 22.7 ± 3.7 |

**Supplementary Table S3.** Effect of heat treatment on 50 µm silica and the presence of QA ligands by zeta potential measurements. All data are derived from three independent experiments (mean ± SD, n = 3).

| **Tests** | **Heat treatment (800°C)** | **QA ligand on silica surface** | **Mean zeta potential (mV)** |
|---|---|---|---|
| **A** | No | No | -29.9 ± 3.50 |
| **B** | Yes | No | -14.2 ± 4.24 |
| **C** | No | Yes | +15.6 ± 4.81 |
| **D** | Yes | Yes | +31.8 ± 6.39 |

The largest negative zeta potential (-29.9mV) was generated from the non-functionalised silica particles (without heat treatment) suspended in water. The zeta potential here is generated from the dissociation of the silanols (Si-OH to Si-$O^{-}$) when exposed to water.

When these silica particles undergo heat treatment, some degree of dehydroxylation of the surface occurs with the loss of silanols[9]. In this case, they produce a less negative zeta potential (-14.2 mV) than those untreated (-29.9 mV). There is also a rearrangement of silanols with a more pronounced isolated silanol character when silica is heated to high enough temperatures (such as 800°C and greater)[10].

For QA-functionalised silicas, whether heat-treated (+31.8mV) or not (+15.6mV) they generated positive zeta potentials, showing that the QA ligand attachment was successful. The permanent positive charge can be attributed to the QA ligand ($N^{+}$). The larger positive zeta potential of the heat-treated sample can be explained by the heat treatment causing the rearrangement of silanols at the surface, leaving more readily available isolated silanols for QA functionalisation.

**Supplementary Table S4.** Refractive index of aqueous solutions of: pure water, proteins (1 mg/mL in water), buffer solution (inactivated SARS-CoV-2), small molecules (1 mg/mL in water) and ethanol (used as a positive control). All data shown are representative of at least three independent experiments.

| | **Refractive index** | |
|---|---|---|
| **Wavelength** | **589.3 nm** | **633 nm** |
| **100% water (blank control)** | 1.3279 | 1.3229 |
| **Bovine serum albumin** | 1.3280 | 1.3230 |
| **Ubiquitin** | 1.3280 | 1.3229 |
| **Cytochrome c** | 1.3280 | 1.3231 |
| **Myoglobin** | 1.3280 | 1.3230 |
| **Buffer solution (inactivated SARS-CoV-2)** | 1.3280 | 1.3231 |
| **Creatinine** | 1.3280 | 1.3249 |
| **Caffeine** | 1.3280 | 1.3230 |
| **Ethanol (positive control)** | 1.3540 | 1.3488 |

Room temperature: 25.1$^{o}$C.

## Supplementary Figures

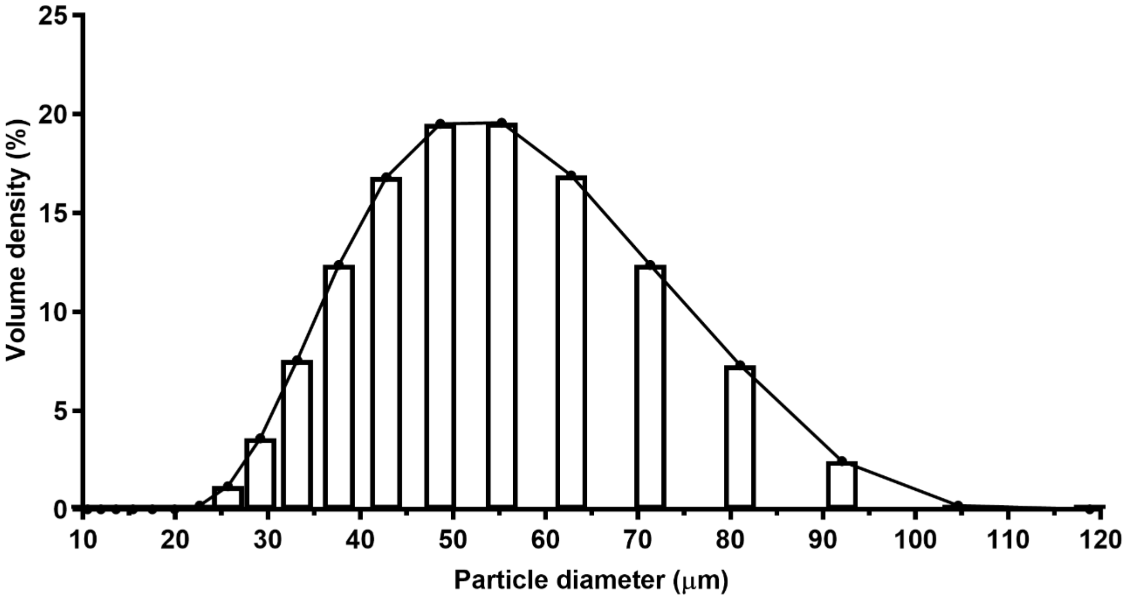


**Supplementary Figure S1.** Particle size distribution of silica particles by Malvern Mastersizer 3000 (Malvern, UK). Mass median diameter (median of volume distribution) = 51.4 ± 0.02 µm. All data are derived from five independent experiments (mean ± SD, *n* = 5).

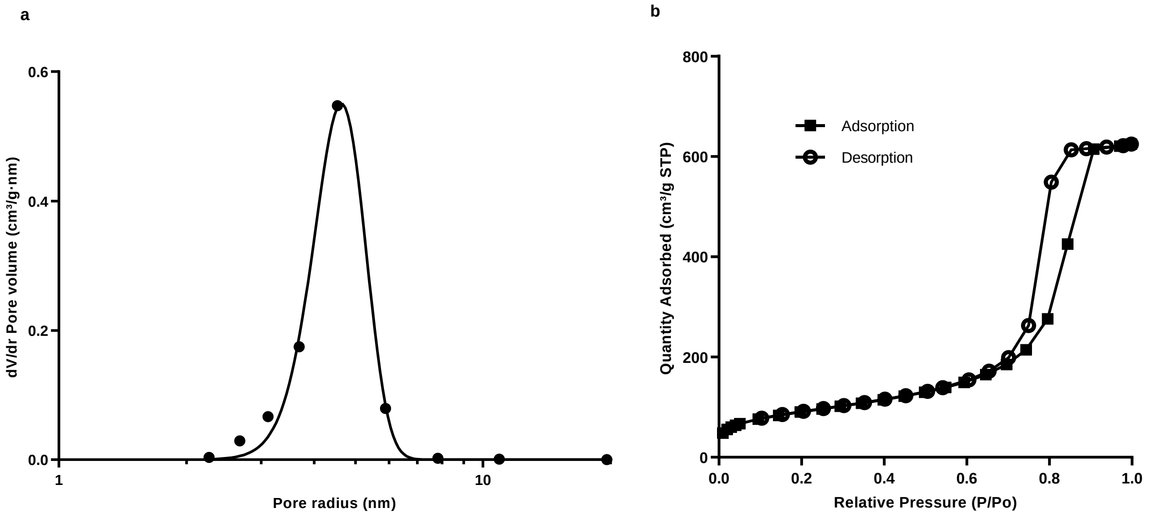


**Supplementary Figure S2. a**. Measurement of pore volume and pore size of non-QA bonded bare silica. The mean pore volume was 0.96 $cm^3$/g and mean pore diameter (radius × 2) was 11.48 nm. **b**. The Brunauer–Emmett–Teller (BET) isotherm displays a Type IV H2 hysteresis. This type is associated with capillary condensation in mesopores.

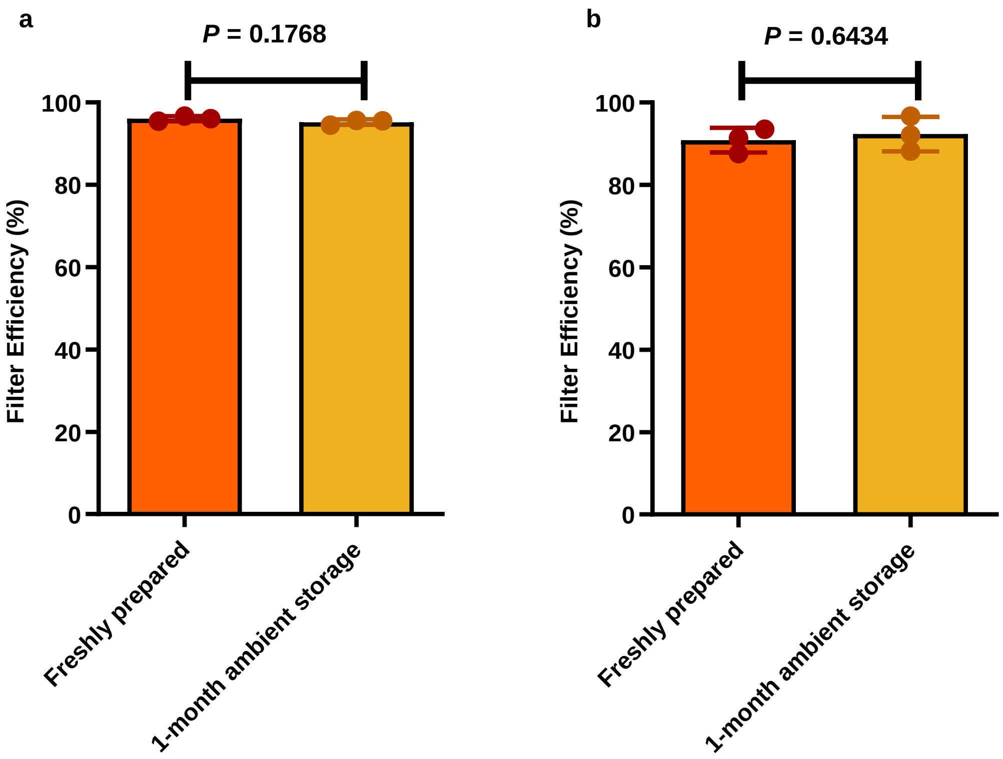


**Supplementary Figure S3.** Storage stability of silica-coated swabs in relation to their filter efficiency for aerosolised proteins. **a**. myoglobin, and, **b**. ubiquitin. The silica-coated cotton materials were stored under ambient conditions for one month before undertaking a filter efficiency test and compared against freshly prepared silica swabs. All data are derived from three independent experiments (mean ± SD, $n = 3$). Comparisons were performed with two-tailed Student's *t*-test.

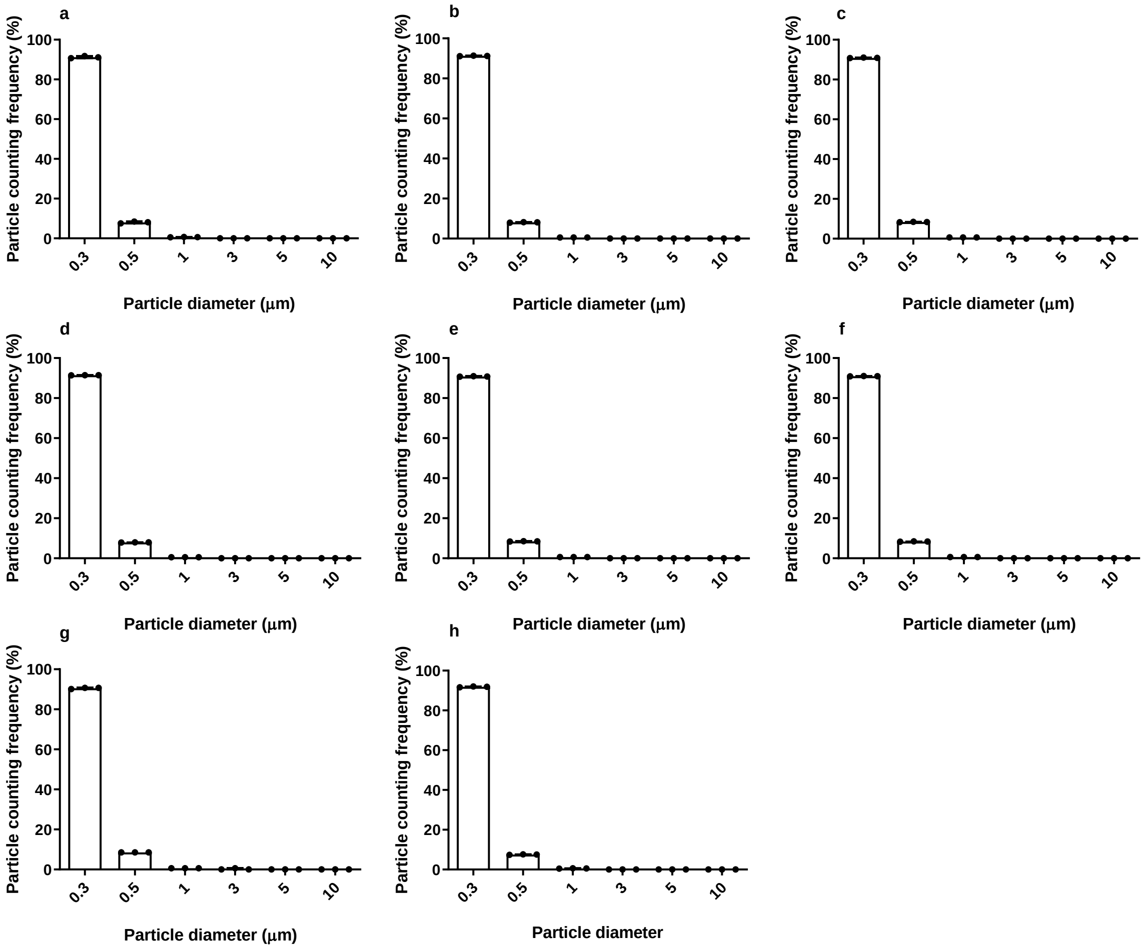


**Supplementary Figure S4.** Particle size distribution of aqueous aerosol containing 0.1 mg/mL of **a**, pure water; **b**, cytochrome c; **c**, BSA; **d**, ubiquitin; **e**, myoglobin; **f**, inactivated SARS-CoV-2 in buffer solution; **g**, creatinine; **h**, caffeine. They were generated with a nebuliser (ORTOREX™ Portable Ultrasonic Nebuliser) and measured with a handheld 6-channel dust particle counter which uses a laser diffraction system (TC-8200, Dongguan Huazhong Instrument Co., Guangdong, China). The most frequent band of particle diameter lies at ~0.3 µm in each case. The concentration of the inactivated SARS-CoV-2 in buffer solution used here was 1.3 x $10^5$ PFU/mL, which was diluted 10 times from the original solution due to instrumental signal saturation. All data are derived from three independent experiments (mean ± SD, *n* = 3).

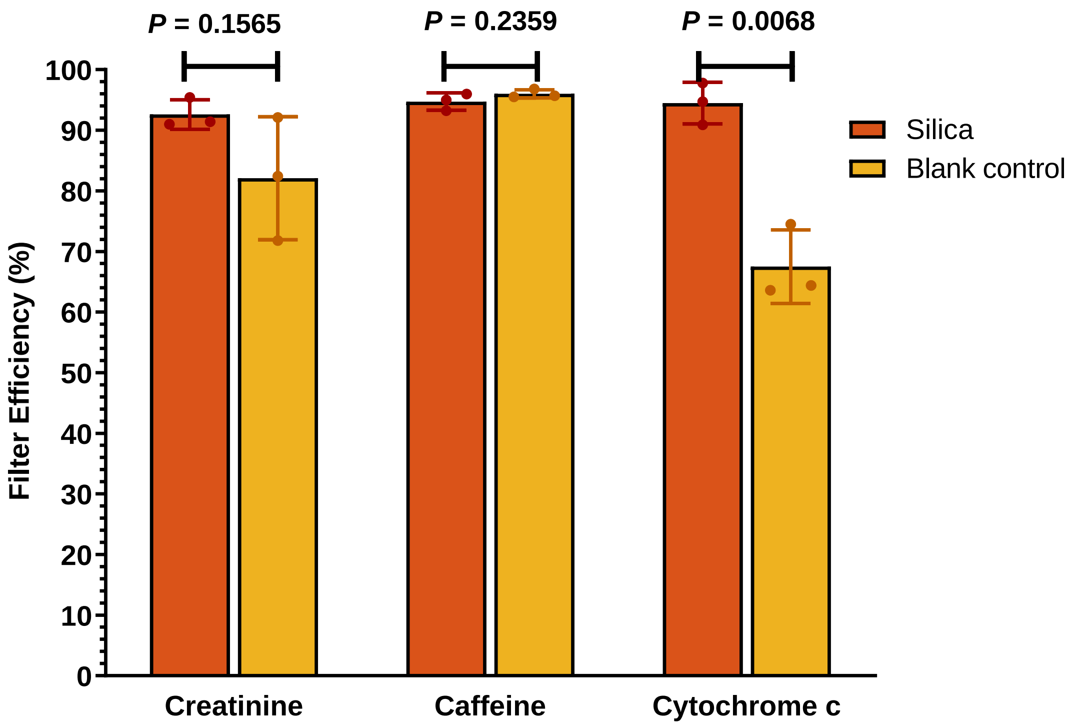


**Supplementary Figure S5.** Filter efficiency of small molecules (creatinine and caffeine) and cytochrome c for the silica coated material and blank material. All data are derived from three independent experiments (mean ± SD, $n$ = 3). Comparisons were performed with two-tailed Student's $t$-test. A solution containing 1 mg/mL of all three analytes, creatinine, caffeine, and cytochrome c, dissolved in water was prepared. Caffeine was chosen as a slightly hydrophobic, low molecular-weight, polar analyte with moderate water solubility under agitation. Creatinine was selected as a low molecular-weight, polar, hydrophilic analyte that is water soluble. The silica swabs saw an increase in filter efficiency for creatinine and cytochrome c, indicating that the silica can enhance the filter efficiency of hydrophilic aerosols, especially proteins. Caffeine showed high filter efficiency even for the uncoated blank control, but the filter efficiency of the silica material decreased slightly, which was not statistically significant. The increase in filter efficiency for cytochrome c was significantly greater than that of creatinine, suggesting that the functionalised silica has a stronger affinity for aerosolised proteins, as anticipated. Furthermore, only cytochrome c showed a statistically significant ($p<0.05$) difference in filter efficiencies between the silica-coated and uncoated swabs.

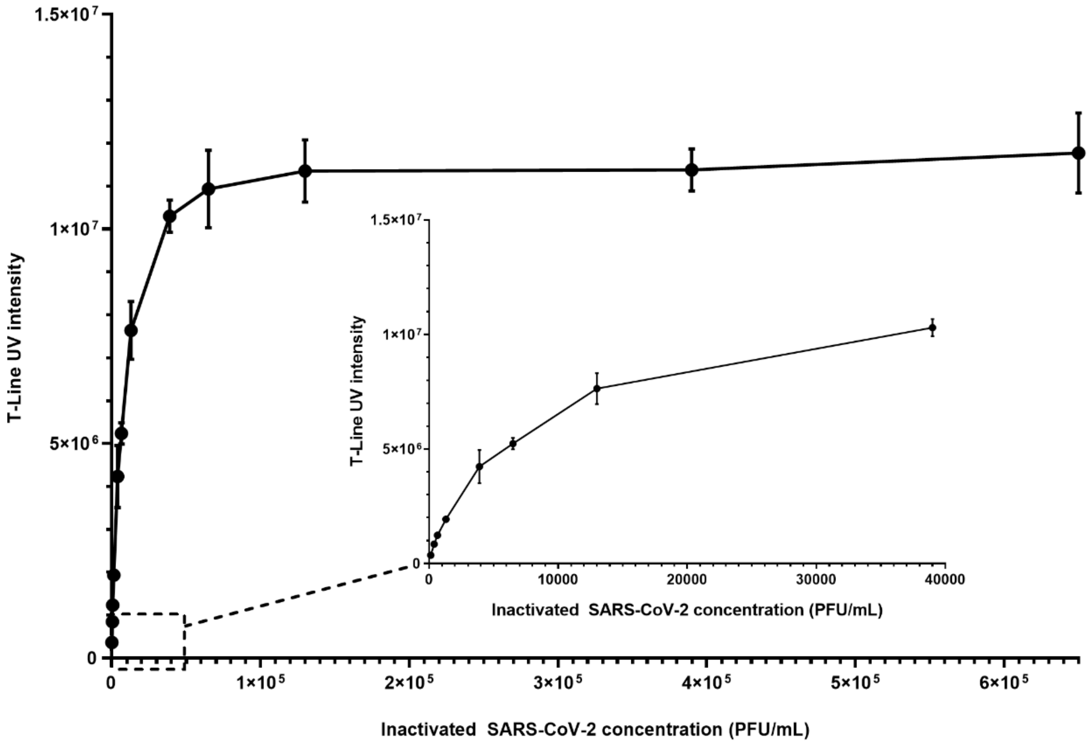


**Supplementary Figure S6.** Calibration curve of lateral flow assay, using GelDoc UV to quantify the intensity of the positive test line. Experimental parameters were controlled to produce post-mask syringe filters concentration (AMC) between $1.3 \times 10^3$ and $1 \times 10^5$ PFU/ml. Before-mask syringe filters (BMC) were diluted to a concentration of $1/10^{th}$ to match those parameters and prevent the concentration exceeding $7 \times 10^4$ PFU/ml and thus being incalculable due to saturation. All data are derived from three independent experiments (mean ± SD, n = 3).

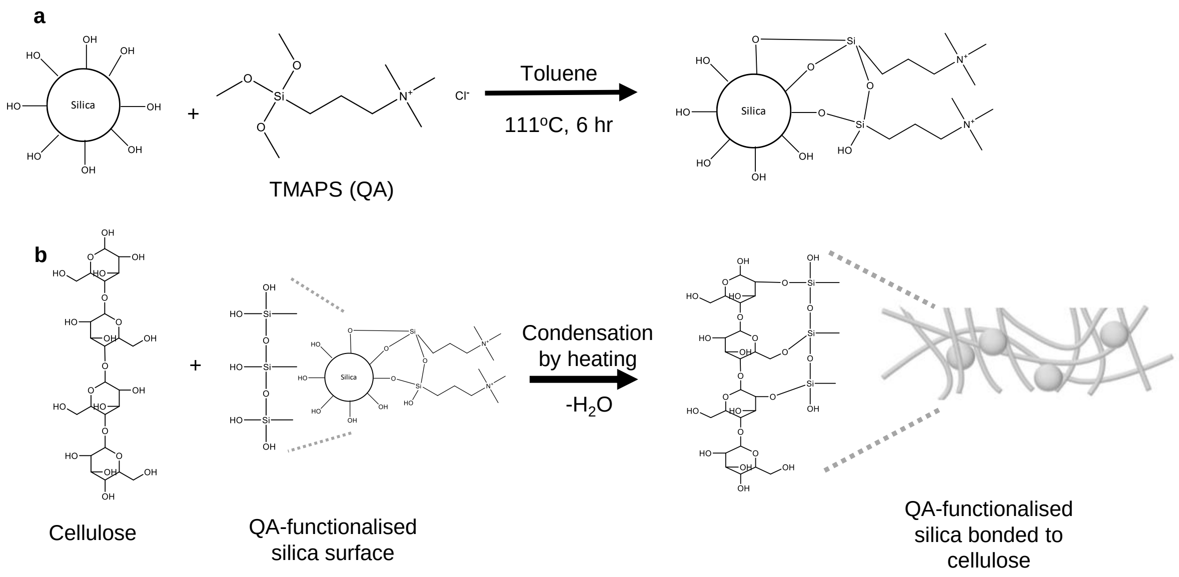


**Supplementary Figure S7.** Schematic representation of QA-functionalised amorphous silica synthesis. **a**, Functionalisation of silica gel with N-trimethoxysilylpropyl-N,N,N-trimethylammonium chloride (TMAPS, a kind of quaternary amine (QA) silane) by stirring the reaction mixture in toluene at 111 $^{o}$C for 6 hr. **b**, Condensation reaction of silanol groups on the surface of QA-functionalised silica to hydroxyl groups of cellulose under heating.

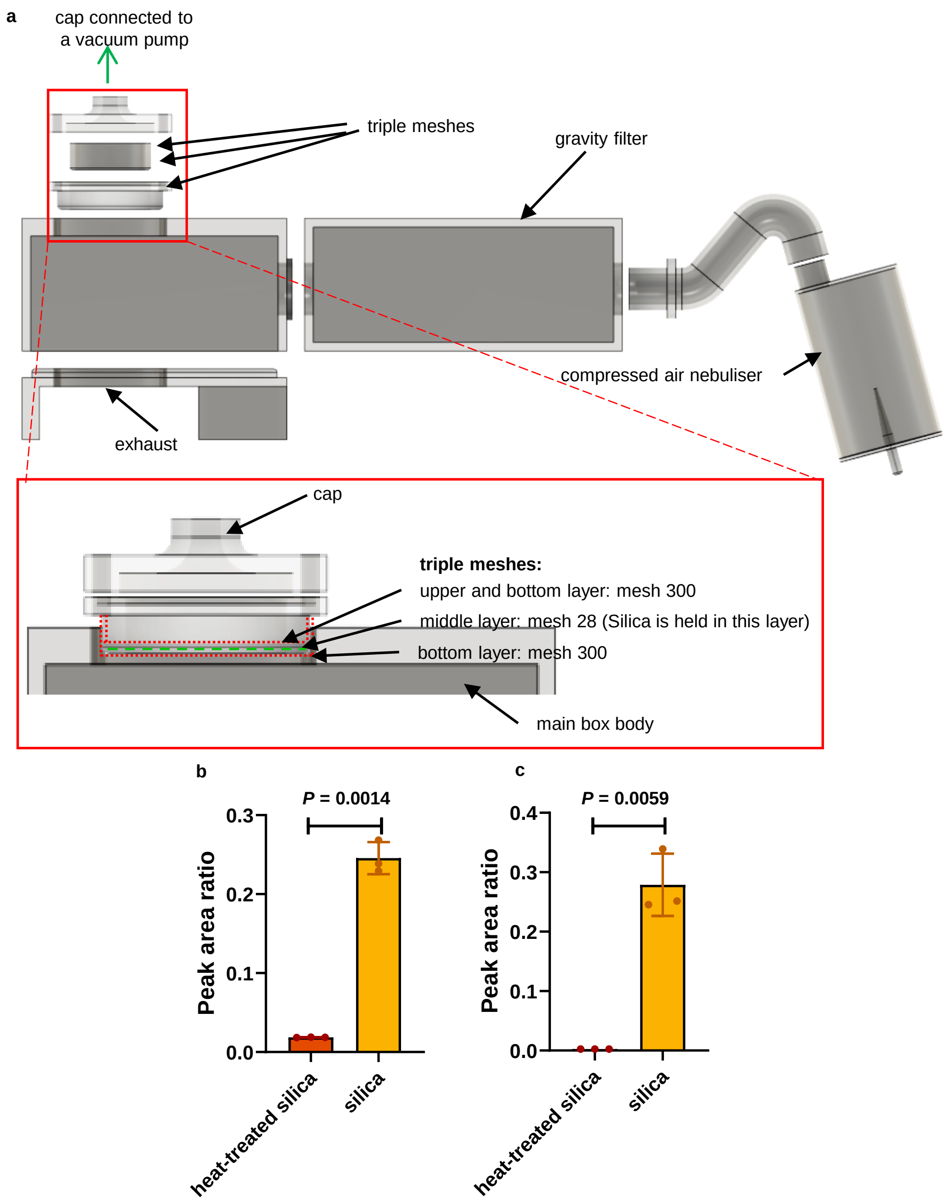


**Supplementary Figure S8.** The capture efficiency of functionalised silica was tested in a fibreless setup (without attaching silica to a fabric substrate) to shed light on the underpinning mechanism responsible for aerosolised protein capture. **a**. Schematic illustration of the fibreless test apparatus. The triple mesh is made from woven stainless steel and holds the unbound silica particles in place. Mesh 300 is a woven stainless-steel mesh with an aperture of 0.055 mm, and mesh 28 is a woven stainless-steel mesh with an aperture of 0.55 mm; refer to Materials and Methods for further details. Silica is held in the mesh support and is exposed to nebulised bovine serum albumin (BSA). **b**. Peak area ratios of BSA to cytochrome c, in which BSA was eluted from the two silica variants after exposure to aerosolised BSA (1 mg/mL in water), and then mixed with cytochrome c prior to MS analysis. **c**. Peak area ratios of BSA to cytochrome c, in which BSA was eluted from the two silica variants after being exposed to BSA (0.01 mg/mL) in solution phase, and then mixed with cytochrome c prior to MS analysis. Details of the experimental method are given in the methods section. All data are derived from three independent experiments (mean ± SD, n = 3). Comparisons were performed with two-tailed Student's *t*-test.

## Supplementary References


1 Brunauer, S., Emmett, P. H. & Teller, E. Adsorption of Gases in Multimolecular Layers. *Journal of the American Chemical Society* **60**, 309-319, doi:10.1021/ja01269a023 (1938).

2 Barrett, E. P., Joyner, L. G. & Halenda, P. P. The Determination of Pore Volume and Area Distributions in Porous Substances. I. Computations from Nitrogen Isotherms. *Journal of the American Chemical Society* **73**, 373-380, doi:10.1021/ja01145a126 (1951).

3 Papadopoulos, S., Jürgens, K. D. & Gros, G. Protein diffusion in living skeletal muscle fibers: dependence on protein size, fiber type, and contraction. *Biophys J* **79**, 2084-2094, doi:10.1016/S0006-3495(00)76456-3 (2000).

4 Graf, M., Galera García, R. & Wätzig, H. Protein adsorption in fused-silica and polyacrylamide-coated capillaries. *Electrophoresis* **26**, 2409-2417, doi:10.1002/elps.200410360 (2005).

5 Renatus, M. *et al.* Structural Basis of Ubiquitin Recognition by the Deubiquitinating Protease USP2. *Structure* **14**, 1293-1302, doi:https://doi.org/10.1016/j.str.2006.06.012 (2006).

6 Nir, I., Huttner, D. & Meller, A. Direct Sensing and Discrimination among Ubiquitin and Ubiquitin Chains Using Solid-State Nanopores. *Biophys J* **108**, 2340-2349, doi:10.1016/j.bpj.2015.03.025 (2015).

7 Peng, H., Zhiming, L., Hengyao, H. & Cui, D. Synthesis and Characterization of Bovine Serum Albumin-Conjugated Copper Sulfide Nanocomposites. *Journal of Nanomaterials* **2010**, doi:10.1155/2010/641545 (2010).

8 Salis, A. *et al.* Measurements and Theoretical Interpretation of Points of Zero Charge/Potential of BSA Protein. *Langmuir* **27**, 11597-11604, doi:10.1021/la2024605 (2011).

9 Zhuravlev, L. T. The surface chemistry of amorphous silica. Zhuravlev model. *Colloids and Surfaces A: Physicochemical and Engineering Aspects* **173**, 1-38, doi:https://doi.org/10.1016/S0927-7757(00)00556-2 (2000).

10 Sunseri, J. *Synthetic Strategies to Improve Silica-Based Stationary Phases for Reversed-Phase Liquid Chromatography* Doctor of Philosophy thesis, Florida State University, (2003).
