# Supplementary material for: Attaching protein-adsorbing silica particles to the surface of cotton substrates for bioaerosol capture including SARS-CoV-2
Source: Nat Commun. 2023 Aug 18;14:5033. doi: 10.1038/s41467-023-40696-x (PMC10439164; doi:10.1038/s41467-023-40696-x)
Supplement: Supplementary file 2 — Reporting Summary [file 41467_2023_40696_MOESM2_ESM.pdf]

nature portfolio

Corresponding author(s): Simon Maher

Last updated by author(s): 14/07/2023

# Reporting Summary

Nature Portfolio wishes to improve the reproducibility of the work that we publish. This form provides structure for consistency and transparency in reporting. For further information on Nature Portfolio policies, see our Editorial Policies and the Editorial Policy Checklist.

## Statistics

For all statistical analyses, confirm that the following items are present in the figure legend, table legend, main text, or Methods section.

| n/a | Confirmed | |
|---|---|---|
| ☐ | ☒ | The exact sample size (*n*) for each experimental group/condition, given as a discrete number and unit of measurement |
| ☐ | ☒ | A statement on whether measurements were taken from distinct samples or whether the same sample was measured repeatedly |
| ☐ | ☒ | The statistical test(s) used AND whether they are one- or two-sided<br>*Only common tests should be described solely by name; describe more complex techniques in the Methods section.* |
| ☒ | ☐ | A description of all covariates tested |
| ☐ | ☒ | A description of any assumptions or corrections, such as tests of normality and adjustment for multiple comparisons |
| ☐ | ☒ | A full description of the statistical parameters including central tendency (e.g. means) or other basic estimates (e.g. regression coefficient) AND variation (e.g. standard deviation) or associated estimates of uncertainty (e.g. confidence intervals) |
| ☐ | ☒ | For null hypothesis testing, the test statistic (e.g. *F*, *t*, *r*) with confidence intervals, effect sizes, degrees of freedom and *P* value noted<br>*Give P values as exact values whenever suitable.* |
| ☒ | ☐ | For Bayesian analysis, information on the choice of priors and Markov chain Monte Carlo settings |
| ☒ | ☐ | For hierarchical and complex designs, identification of the appropriate level for tests and full reporting of outcomes |
| ☒ | ☐ | Estimates of effect sizes (e.g. Cohen's *d*, Pearson's *r*), indicating how they were calculated |

*Our web collection on statistics for biologists contains articles on many of the points above.*

## Software and code

Policy information about availability of computer code

| | |
|---|---|
| Data collection | Protein computational images were produced using PyMol v2.5 software and the Adaptive Poisson-Boltzmann Solver (APBS) electrostatic plugin. Mass spectrometry data were collected with Waters MassLynx software (version 4.1), lateral flow assay data were collected by GelDoc Go Gel Imaging System (BioRad). Water contact angle was collected by Drop shape analyser (Krüss-DSA100). Particle size distribution of silica particles were collected by Malvern Mastersizer 3000 (Malvern, UK). The emitted aerosol particle size distributions were characterised using a handheld 6-channel dust particle counter which uses a laser diffraction system (TC-8200, Dongguan Huazhong Instrument Co.) Surface area and porosity of silica particle were determined using a Micromeritics 3-Flex instrument (Norcross, GA, USA). Zeta potential measurement was carried out using Zetasizer nano ZS (Ver. 8.01.4906, Malvern Panalytical Instruments, UK). A CAD image of the test rig design was created with Autodesk 360 (version 2.0.15050). SEM images were taken with Hitachi S4800 SEM and JEOL JSM 7001F SEM systems. |
| Data analysis | The intensity of the lateral flow assay lines were analysed using Image Lab software (BioRad, version 3.0.0.07). Statistical analysis was carried out with GraphPad Prism 8 software (version 8.0.2). |

For manuscripts utilizing custom algorithms or software that are central to the research but not yet described in published literature, software must be made available to editors and reviewers. We strongly encourage code deposition in a community repository (e.g. GitHub). See the Nature Portfolio guidelines for submitting code & software for further information.

## Data

Policy information about availability of data

All manuscripts must include a data availability statement. This statement should provide the following information, where applicable:

- Accession codes, unique identifiers, or web links for publicly available datasets
- A description of any restrictions on data availability
- For clinical datasets or third party data, please ensure that the statement adheres to our policy

The data generated or analysed during this study are presented in the published article and corresponding supplementary information files.

## Human research participants

Policy information about studies involving human research participants and Sex and Gender in Research.

| | |
|---|---|
| Reporting on sex and gender | n/a |
| Population characteristics | n/a |
| Recruitment | n/a |
| Ethics oversight | n/a |

Note that full information on the approval of the study protocol must also be provided in the manuscript.

# Field-specific reporting

Please select the one below that is the best fit for your research. If you are not sure, read the appropriate sections before making your selection.

☒ Life sciences ☐ Behavioural & social sciences ☐ Ecological, evolutionary & environmental sciences

For a reference copy of the document with all sections, see nature.com/documents/nr-reporting-summary-flat.pdf

# Life sciences study design

All studies must disclose on these points even when the disclosure is negative.

| | |
|---|---|
| Sample size | No predetermined sample size calculation was performed as this study does not involve animal or human subjects. The sample size was chosen as sufficient based on common practice, a statistically significant improvement in the performance of the silica mask compared to the blank control and the observed variation. The number of replicates is shown in the table and figure legends where relevant. |
| Data exclusions | No data were excluded. |
| Replication | All experiments were performed with appropriate replicates, as described in the figure titles and Methods section. At least 3 independent replicates were performed. |
| Randomization | Randomisation is not applicable to our project, as this is not a case-control study. |
| Blinding | Blinding was not applied during data collection and outcome assessment because the study is concerned with the characterisation of a face mask and does not involve individual difference. Thus a blinding study is not relevant to this work. |

# Reporting for specific materials, systems and methods

We require information from authors about some types of materials, experimental systems and methods used in many studies. Here, indicate whether each material, system or method listed is relevant to your study. If you are not sure if a list item applies to your research, read the appropriate section before selecting a response.

## Materials & experimental systems

| n/a | Involved in the study |
| --- | --- |
| ☐ | ☒ Antibodies |
| ☐ | ☒ Eukaryotic cell lines |
| ☒ | ☐ Palaeontology and archaeology |
| ☒ | ☐ Animals and other organisms |
| ☒ | ☐ Clinical data |
| ☒ | ☐ Dual use research of concern |

## Methods

| n/a | Involved in the study |
| --- | --- |
| ☒ | ☐ ChIP-seq |
| ☒ | ☐ Flow cytometry |
| ☒ | ☐ MRI-based neuroimaging |

# Antibodies

| | |
| --- | --- |
| Antibodies used | FlowFlex SARS CoV-2 Antigen Rapid Tests (lateral flow assay kit, ACON Biotech (Hangzhou) Co., Ltd., Ref: L031-118Y5, Lot: A1100002) were used. |
| Validation | The kit is available at https://www.flowflexrapidtest.com/product/single-flowflex-sars-cov-2-antigen-rapid-test/<br>Validation report can be found on the user instruction menu and company website (https://www.flowflexrapidtest.com/wp-content/uploads/2021/03/Clinical-Study-Report.pdf) |

# Eukaryotic cell lines

Policy information about cell lines and Sex and Gender in Research

| | |
| --- | --- |
| Cell line source(s) | Vero E6 cells (isolated from African green monkey kidney cells) (VERO C1008, American Type Culture Collection, Manassas, VA, USA) were obtained from Public Health England (The UK Health Security Agency) |
| Authentication | We did not independently authenticate this cell line. |
| Mycoplasma contamination | All cell lines used were routinely tested and found negative for mycoplasma contamination before use in infection assays. |
| Commonly misidentified lines<br>(See ICLAC register) | There are no commonly misidentified lines. |
