# Supplementary material for: Attaching protein-adsorbing silica particles to the surface of cotton substrates for bioaerosol capture including SARS-CoV-2
Source: Nat Commun. 2023 Aug 18;14:5033. doi: 10.1038/s41467-023-40696-x (PMC10439164; doi:10.1038/s41467-023-40696-x)
Supplement: Supplementary file 3 — Peer Review [file 41467_2023_40696_MOESM3_ESM.pdf]

nature portfolio

Peer Review File

Attaching Protein-Adsorbing Silica Particles to the Surface of Cotton Substrates for Bioaerosol Capture including SARS-CoV-2

Open Access This file is licensed under a Creative Commons Attribution 4.0 International License, which permits use, sharing, adaptation, distribution and reproduction in any medium or format, as long as you give appropriate credit to the original author(s) and the source, provide a link to the Creative Commons license, and indicate if changes were made. In the cases where the authors are anonymous, such as is the case for the reports of anonymous peer reviewers, author attribution should be to 'Anonymous Referee' followed by a clear attribution to the source work. The images or other third party material in this file are included in the article's Creative Commons license, unless indicated otherwise in a credit line to the material. If material is not included in the article's Creative Commons license and your intended use is not permitted by statutory regulation or exceeds the permitted use, you will need to obtain permission directly from the copyright holder. To view a copy of this license, visit http://creativecommons.org/licenses/by/4.0/.

Editorial Note: Reviewer #4 was recruited to look over the responses of Reviewer #1

REVIEWER COMMENTS

Reviewer #1 (Remarks to the Author):

This research describes a facile method for the production of cotton-based face masks bearing quaternary-modified porous silica microspheres with the aim to improve the virus filtration efficiency of the face mask due to the strong interaction between SiO2 and proteins on the virus surface. I like the idea but I think the following tasks are required to be done before publication:
1) SEM images from amorphous microporous spherical silica (QA-SiO2) with higher magnification are required to clearly show the porosity of SiO2 particles.
2) It is not clear how they made the SiO2 particles and how they modified it with QA groups? What is the inserted quaternary ammonium (QA) group? There are many QAs. The authors have mentioned “Amorphous microporous spherical silica (Figure 1) was produced using a published sol-gel method70”, but Ref 70 is a review paper and does not describe the methods for the preparation of materials.
3) What is the interaction between cotton (cellulose) with QA-SiO2 which stabilizes the silica particles on the cotton surface?
4) I do not fully agree with the discussion related to Figure S1. Indeed, higher adsorption (and interaction) of proteins (a polymeric material) on silica in comparison to non-polymeric materials (such as creatinine and caffeine) is probably related to the number of interactions which the ultimate effect is the stronger silica/proteins interaction than silica/small-molecule interaction.
5) How is the stability of silica particles on the cotton surface? Are there any changes in the silica concentration after several hours inhalation-exhalation process? As the authors have mentioned, the risk of inhalation of immobilized silica particles is of great concern and needs to be checked carefully.
6) There is one similar report (https://doi.org/10.3390/ijerph19063639) which I suggest the authors consider and highlight the differences.

Reviewer #2 (Remarks to the Author):

Dear authors, congratulations on the manuscript you have produced. It will undoubtedly help in the fight against COVID-19 (and similar ones).

Here are some recommendations for the text:

Abstract
Line 24, please correct the word commonplace.

Line 24,25: “Severe acute respiratory syndrome coronavirus 2 (SARS-CoV25 2) has a spherical particle size of ~97 nm”. It might be better to write: “The severe acute respiratory syndrome caused by coronavirus 2 (SARS-CoV-2), which has a spherical particle size of ~97 nm”.
We are talking about the size of the virus and not the respiratory syndrome.

Line 30: Change “fibres” to fibers.

Lines 31,32: Change “aerosolized” to aerosolized.

Figure Graphical abstract: please indicate the size of the particles on the figure.

Introduction

Line 51, The particles produced by coughing have a typical size range of ~0.6-500 μm13. It differs from what you told in the abstract (typically>600nm). Please correct the abstract. ~0.6-500 μm is the correct information.

Line53, correct spelling for aerosolized

Line 54, correct spelling for defenses

Line 60,61: “it has been shown by Hazard et al that the inclusion of a valve reduces filter efficiency”. This type of mask only protects the virus from entering the individual and does not prevent its spreading. If you want, you can add this information.
Line 94,95,96. Please correct the words patchwise, hypothesized, and aerosolized.

Line 98. The surface properties of amorphous silica depend.

Line 111, correct spelling for idealize

Line 115, fiber

Line 120, minimize

Lines 122-123, aerosolized, characterized, idealized. The text is
full of spelling errors. Please do a general spell check. From now on, I will no longer point out this type of mistake.

Results and Discussion
Selection of particle size and porosity of amorphous silica

Line 133-137: “Although smaller silica particle sizes (< 60 µm in diameter) can increase the overall surface area available and can likely improve capture efficiency of aerosolized proteins and virus, they may increase the risk of inhalation of silica dust”. How to ensure that the user does not inhale silica particles (or fragments)? Was any testing done? Inhaling these particles would be a disaster.

Filter efficiency of silica-coated cotton material for common aerosolized proteins (suggestion for subtitle)

Filter efficiency of silica-coated cotton material for aerosolized inactivated SARS-CoV-2 proteins (suggestion for subtitle)
Both these tests and results were fascinating!

Filter efficiency of silica-coated cotton material vs commercially available cotton face covering
Line 218, Did you mean table 1 and not table 2

‘Breathability’ testing
Line 227, Why use an airflow rate of 85 L/min? Is this flow close to that of human breathing? How much is it? Explain

Conclusion
Please, speak in terms of the percentage how much your method can increase the filtering efficiency of the masks.

Filter efficiency test
When Reading, I wish the method were better explained in detail, explaining figures 6 and 7 in this part of the text.

I want to congratulate you on the work you have done. I understand that this method can be fascinating, and the result has outstanding figures.
But I missed a few things in the text:
1) Can they be washed when these masks are submitted to the process with silica?
2) Do these mascaras degrade over time? Hours, days?
3) Is there any risk of inhaling silica?
4) How to compare the results with other mascaras on the market and other studies? Use the quality factor described by Zangmeister et al. 2020 and World Health Organization (WHO)
5) Need to improve the language (English) of all text. It is full of orthographic errors.

Reviewer #3 (Remarks to the Author):

[Note from the editor: Please see also attached PDF]

This manuscript evaluates the effectiveness of a novel, silca-functionalized, cotton filter material at capturing biological aerosol particles.

Upon review of this manuscript, I find that the evaluation of the functionalized cotton filters is intriguing, but does not demonstrate, the mechanism of removal described. The primary issues that I have with this manuscript are the methods used to derive the data and the interpretation of the data, due primarily to the limitations in the methods. The most significant methodological issue is the lack of aerosol size distributions. The authors present a characterization of the nebulizer, but do not say material was nebulized, nor at what concentration. Further, the methods only outline the concentration of non-protein materials that were nebulized, and not the proteins. These should be presented. Further, size distributions of each material should be presented. Changes in aerosol size can have significant impacts on filtration efficiency. The data presented here, do not conclusively demonstrate that the filtration of protein containing particles is enhanced over non-protein containing particles. They only indicate that the filtration efficiency for the different experiments is different, without conclusively identifying a mechanism. Therefore, the claim that the filter enhances the biological filtration through protein capture is not demonstrated. The authors need to present size distribution data for each material, and the complete description of the aerosol stock solution, including both the concentration of target material (protein etc.), and the diluent used in order to allow for evaluation of the claims. Without this data, it is impossible to separate the physical filtration efficiency from the potential enhancements due to selective biological filtration efficiency. Finally, there is a logical flaw in the description of the potential enhancement to filtration efficiency, as it is described. In order to for any filter (functionalized or not) to capture a particle it must impact the surfaces of the filter, in this case the filter fibers. Therefore, any chemical enhancements to filtration would actually be in the retention of particles on the fibers through adsorption, and if the action of the functionalized filters were purely chemical, that would indicate that non-functionalized filters collected particles at the same rate as the functionalized filters, but if the filter were non-functionalized, some fraction of the particles wouldn't "stick" to the filter fibers. Based on the SEM image presented in Figure 1, it seems to me that the dramatic increase in surface area resulting from functionalization with silica results in increased capture efficiency, rather than increased retention efficiency.

All that said, I do believe that this filter material results in a significant advance in filtration technology. I simply think that it is a result of enhanced physical filtration efficiency, rather than an enhancement in biological retention efficiency. I admit that the data don't clearly indicate which mechanism is dominating. In order to clearly identify the true mechanism more work is required:

1. A size-resolved physical filtration measurement is necessary. There are several standard methods that could be leveraged.
2. Size-resolved filtration efficiency measurements for each material evaluated.
3. Comparison of 1 and 2 will clearly identify the impact of particle composition on filtration efficiency and more clearly identify the mechanism.

Regardless of the mechanism of action, I believe this material represents an important advance in filtration technology, but in order to be publishable, the above data need to be collected.

I have included additional comments in the attached PDF file.

Referee comments are written in blue-coloured font, responses used black coloured-font.

Tracked changes to the original submission file can be readily observed in one of the accompanying files uploaded. Furthermore, edits in the revised manuscript have been colour-coded according to:

Reviewer 1: edits marked in red in the revised manuscript
Reviewer 2: edits marked in green in the revised manuscript
Reviewer 3: edits marked in blue in the revised manuscript

Reviewer #1:

This research describes a facile method for the production of cotton-based face masks bearing quaternary-modified porous silica microspheres with the aim to improve the virus filtration efficiency of the face mask due to the strong interaction between SiO2 and proteins on the virus surface. I like the idea but I think the following tasks are required to be done before publication:

Q1: SEM images from amorphous microporous spherical silica (QA-SiO2) with higher magnification are required to clearly show the porosity of SiO2 particles.

Response:

We are happy to provide this. An additional SEM image showing the silica particle porosity has been included in the revised manuscript (Figure 1b).

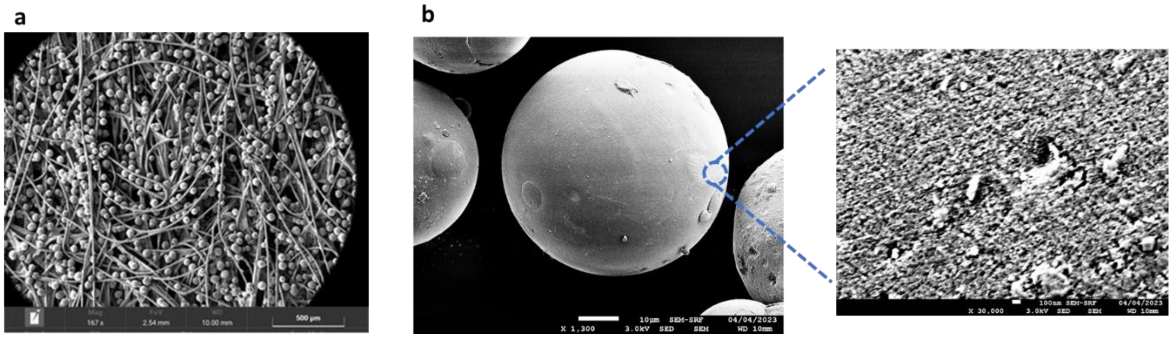

Figure 1. SEM images of a, QA functionalised silica attached to the substrate, and, b, silica particle (x1300) with a magnified image showing pores visible on the surface (x30,000).

Furthermore, we measured the surface area, pore volume and pore diameter of the silica, and have included this data in the subsection entitled, "***Porosity of amorphous silica***" (within **Result and Discussion** section):

Porosity of amorphous silica

… The non-functionalised silica used in this study has a surface area of 320.24 m^2 g^{-1}, a mean pore volume of 0.96 cm^3 g^{-1} and a mean pore diameter of 11.48 nm (Figure S2a). The BET isotherm, which is correlated to surface area, displays a Type 4 H2 hysteresis (Figure 2b), indicating capillary condensation in mesopores. Further details about the measurement methods and graphical results can be found in supplementary materials and Figure S2a.

The measurement methods and parameters used for these analyses are also listed in the Method section of the **Supplementary materials**:

Surface area and porosity of silica particle by nitrogen adsorption

To determine the surface area, pore volume, and pore size of the bare silica particles prior to QA functionalisation, we performed nitrogen adsorption−desorption measurements using a Micromeritics 3-Flex instrument (Norcross, GA, USA). The Brunauer−Emmett−Teller (BET) surface area was calculated by applying the BET equation to the adsorption data [1], while the pore size distribution was determined using the Barrett− Joyner−Halenda (BJH) method [2], applied to the adsorption branch of the sorption isotherms.

All samples were degassed at 150 °C for 3 h before measurement. The specific surface area (6 points, 0.025 < P/P_0 < 0.30) and pore size distribution were calculated using the multi-point BET equation and BJH model, respectively. The total pore volume was determined at the last point of the adsorption branch, corresponding to a relative pressure of 0.99.

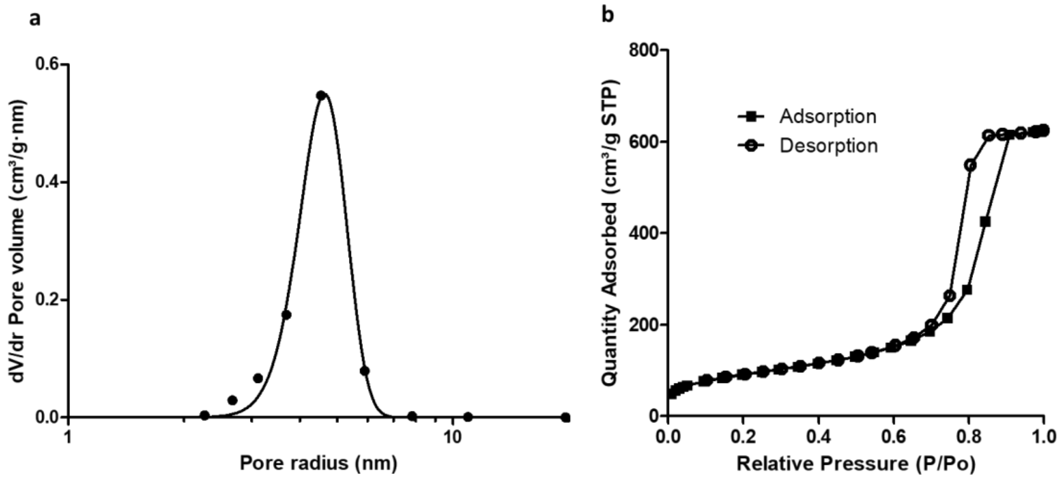

Supplemental Figure S2 a. Measurement of pore volume and pore size of non-QA bonded bare silica. The mean pore volume was 0.96 cm^3/g and mean pore diameter (radius x 2) was 11.48 nm. b. The BET isotherm displays a Type 4 H2 hysteresis. This type is associated with capillary condensation in mesopores.

Q2: It is not clear how they made the SiO2 particles and how they modified it with QA groups? What is the inserted quaternary ammonium (QA) group? There are many QAs. The authors have mentioned “Amorphous microporous spherical silica (Figure 1) was produced using a published sol-gel method70”, but Ref 70 is a review paper and does not describe the methods for the preparation of materials.

Response: We completely agree with the reviewer that more detail is required. We have added an additional section within Materials and Methods describing in detail the synthesis of SiO2 particles and their modification with QA groups. In this study, we used N-trimethoxysilylpropyl-N,N,N-trimethylammonium chloride as the QA. A new schematic diagram (Figure S7) has also been included to provide additional details. The information is included in the ***Silica-coated cotton face covering*** subsection.

Silica-coated cotton face covering

Synthesis of amorphous silica and QA functionalisation

QA-functionalised amorphous microporous spherical silica was synthesised using a process based on the method of Stober, et al. [3]. The spherical silica particles were prepared by hydrolysing and condensing tetraethyl orthosilicate (TEOS) with long-chain n-alkylamines in aqueous ammonium hydroxide solution at pH 10, with isopropanol as the co-solvent. Figure S7 provides a schematic diagram illustrating the key steps involved in the synthesis of QA-functionalised silica.

In a typical synthesis procedure, a solution of dodecylamine in a mixed solution of isopropanol (0.16 L) and water (0.1 L) was prepared. Tetraethyl orthosilicate (TEOS) was then slowly added dropwise to the solution at different temperatures with magnetic stirring. As TEOS was added, the clear solution gradually became opaque due to the formation of a white precipitate. The mixture was continuously stirred for 4 h, after which the white precipitate was filtered and repeatedly washed with water and ethanol four times. The resulting product was then dried in a vacuum oven at 80°C for 4 h.

The reaction is initiated with a homogeneous solution at room temperature or higher, and the average particle diameter of the precipitated particles can be controlled by adjusting the water-TEOS molar ratio in the starting solution, allowing for the production of nearly monodisperse particles with a narrow size distribution.

Surface derivatisation of the silica gel with a QA ligand was prepared with N-trimethoxysilylpropyl-N,N,N-trimethylammonium chloride (TMAPS, a kind of QA silane). Typically, a 10 g aliquot of previously synthesised silica gel was dried in a vacuum oven at 150°C for 16 hours. The dried silica gel was then suspended in 100 mL of dry toluene in a 3-neck round-bottom flask connected to a reflux condenser. This mixture was heated to 40°C with slow stirring to form a slurry, and then 15 mL of TMAPS was added. The reaction mixture was stirred for 6 hours at 111°C to activate the QA ligand on the silica surface. Afterwards, the activated silica particles were washed successively with methanol and dried at 80°C for 16 hours.

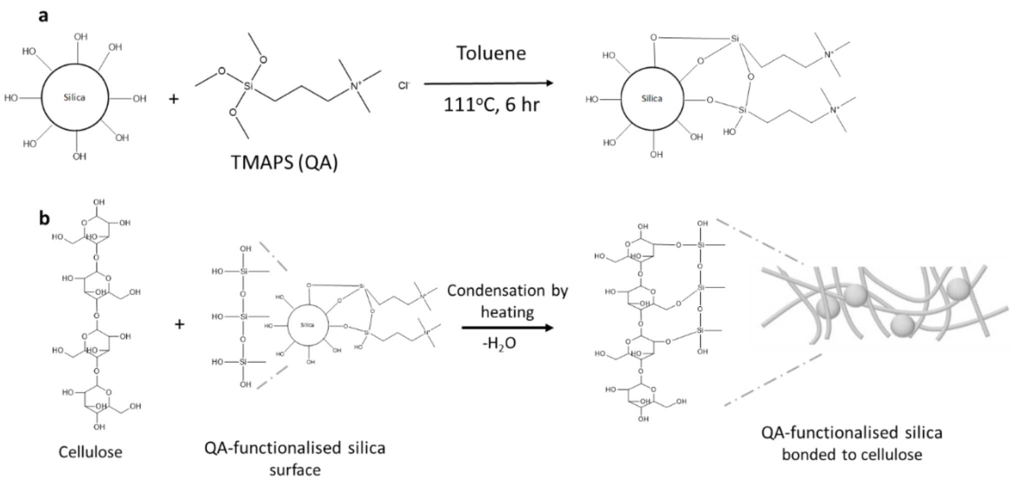

Supplemental Figure S7 Schematic representation of QA-functionalised amorphous silica synthesis. a, Functionalisation of silica gel with N-trimethoxysilylpropyl-N,N,N-trimethylammonium chloride (TMAPS, a kind of quaternary amine (QA) silane) by stirring the reaction mixture in toluene at 111 °C for 6 hr. b, Condensation reaction of silanol groups on the surface of QA-functionalised silica to hydroxyl groups of cellulose under heating.

Q3: What is the interaction between cotton (cellulose) with QA-SiO2 which stabilizes the silica particles on the cotton surface?

Response:

The stability of the silica particles on the cotton surface is due to the interaction between cotton (cellulose) with QA-SiO2 (Figure S7b). Under thermal treatment, a condensation reaction occurs between the silanol groups (-Si-OH) on the surface of QA-functionalised silica and the C-OH group of cellulose forming Si-O-C bonds [4,5]. We have added this explanation to the ***Morphology and stability of silica-bonded cotton*** subsection within the Results and Discussion, and a new schematic diagram (Figure S7) has been included to illustrate these details.

Morphology and stability of silica-bonded cotton

… the interaction between cotton (cellulose) with QA-SiO2 that attaches the silica particles is shown in Figure S7b, where a condensation reaction occurs between silanol groups (-Si-OH) on the surface of the QA-functionalised silica and C-OH groups of cellulose under thermal treatment to form Si-O-C bonds [4,5].

Q4: I do not fully agree with the discussion related to Figure S1. Indeed, higher adsorption (and interaction) of proteins (a polymeric material) on silica in comparison to non-polymeric materials (such as creatinine and caffeine) is probably related to the number of interactions which the ultimate effect is the stronger silica/proteins interaction than silica/small-molecule interaction.

Response:

We thank the reviewer for making this point which we agree with. It is true that proteins can have more interaction with silica due to their larger size, as well as their unique hydrophobic and hydrophilic conformation with surface charge distributions that are very different from small molecules.

We have revised our manuscript accordingly with the following additional text (Results and Discussion):

> ***Filter efficiency of silica-coated cotton material for common aerosolised proteins***
>
> *Silica particles may exhibit more selectivity towards aerosolised proteins due to their unique physical properties, which differ from small molecules (Table S1 and Figure 3). Proteins have both hydrophobic and hydrophilic side chains, with capacity for electrostatic and hygroscopic interactions[6]. The polymeric proteins tested (e.g., cytochrome c, MW 12.4k Da) also exhibit a larger conformation than smaller molecules, such as caffeine (MW 194 Da) and creatinine (MW 113 Da). These factors should increase the probability of silica-protein interactions[7]. The proteins aerosolised in this study also had different isoelectric points (pI, 4.8 - 9.6) and water contact angles. The electrostatic maps of surface charges for each of the four proteins in Figure 3 show varying degrees of positive and negative surface charge distributions. These surface charges are attributed to basic amino acids carrying positive charges and acidic amino acids carrying negative charges at normal physiological pH [8]. Upon contact with the aerosolised proteins, the amide group of the QA ligands on the silica become positively charged, while unreacted areas of the silica have a net negative charge through silanol dissociation (Si-O-), further facilitating electrostatic interaction with charged regions of the proteins. Overall, all of these factors contribute to an increased likelihood of silica-protein interactions, facilitating protein-adsorption onto the silica surface.*

Supplementary materials:

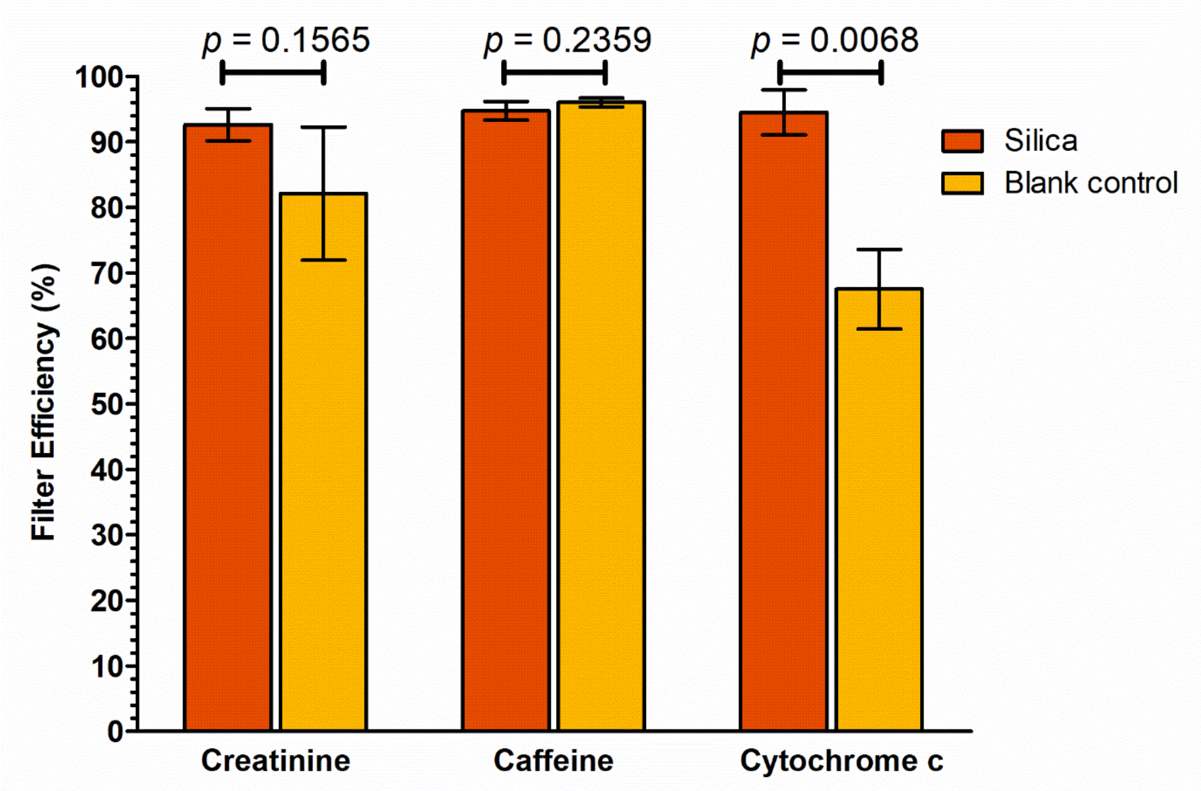

Supplemental Figure S5. Filter efficiency of small molecules (creatinine and caffeine) and cytochrome c for the silica coated material and blank material. Data were presented as mean ± SD (n = 3). Comparisons were performed with two-tailed Student's t-test.

A solution containing 1 mg/mL of all three analytes, creatinine, caffeine, and cytochrome c, dissolved in water was prepared. Caffeine was chosen as a slightly hydrophobic, low molecular-weight, polar analyte with moderate water solubility under agitation. Creatinine was selected as a low molecular-weight, polar, hydrophilic analyte that is water soluble.

The silica swabs saw an increase in filter efficiency for creatinine and cytochrome c, indicating that the silica can enhance the filter efficiency of hydrophilic aerosols, especially proteins. Caffeine showed high filter efficiency even for the uncoated blank control, but the filter efficiency of the silica material decreased slightly, which was not statistically significant. The increase in filter efficiency for cytochrome c was significantly greater than that of creatinine, suggesting that the functionalised silica has a stronger affinity for aerosolised proteins, as anticipated. Furthermore, only cytochrome c showed a statistically significant ($p<0.05$) difference in filter efficiencies between the silica-coated and uncoated swabs.

5) How is the stability of silica particles on the cotton surface? Are there any changes in the silica concentration after several hours inhalation-exhalation process? As the authors have mentioned, the risk of inhalation of immobilized silica particles is of great concern and needs to be checked carefully.

Response:

We appreciate the reviewer's inquiry regarding silica stability. To address this, we conducted an experiment where we left three silica swabs under ambient storage conditions for one month. Afterwards, we tested their filter efficiency with aerosolised proteins, following the same method as described for freshly prepared samples. The results showed that there was no significant increase or decrease in the filter efficiency of aerosolised proteins compared to the freshly prepared swabs. We have included Figure S3 to highlight the stability observation, and the results demonstrate that the silica-bonded swab has good stability. We have included this observation in the manuscript within the ***Morphology and stability of silica-bonded cotton*** section.

The reviewer also points out some safety concerns including the possibility of silica inhalation. Crystalline silica dust exposure can lead to silicosis[9]. However, in this project, we use amorphous silica. According to a report by the US Agency for Toxic Substances and Disease Registry[10], studies of amorphous silica in workers and laboratory animals have not found any evidence of cancer. There are no known health effects from exposure to amorphous silica at the levels found in the environment or in commercial products. Furthermore, we have used a fusible material to keep it trapped preventing the release of silica particles. Fundamentally, it is important to note that this is proof-of-concept research, and as such the activities have been carried out in a controlled environment without human subjects. In the future, if this material were to be used with human participants, further testing and optimisation might be required in order to satisfy local safety and ethical concerns, but this is beyond the scope of this current early stage research study.

Morphology and stability of silica-bonded cotton

… Results from a preliminary stability test indicate that the silica swabs stored under ambient conditions for one month exhibited no significant difference in filter efficiency of aerosolised proteins, compared to freshly prepared swabs (Figure S3, $p > 0.05$). This suggests that the silica swabs remain stable and useable for at least one month.

Supplementary materials:

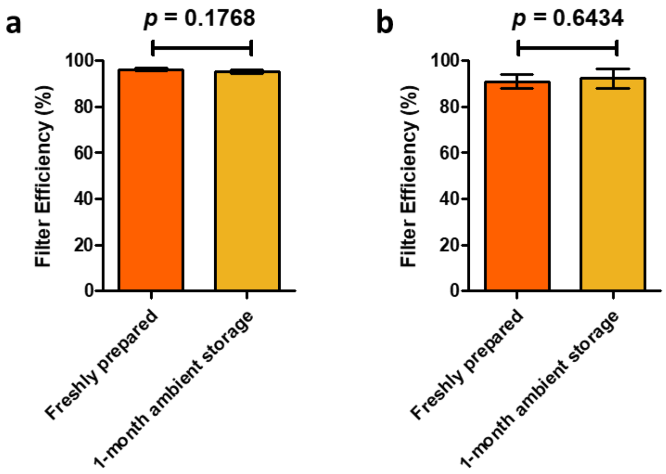

Supplemental Figure S3. *Storage stability of silica-coated swabs in relation to their filter efficiency for aerosolised proteins. a. myoglobin, and, b. ubiquitin. The silica-coated cotton materials were stored under ambient conditions for one month before undertaking a filter efficiency test and compared against freshly prepared silica swabs.*

6) There is one similar report (https://doi.org/10.3390/ijerph19063639) [11] which I suggest the authors consider and highlight the differences.

Response:

We sincerely thank the reviewer for pointing this out to us. We search the literature diligently and are committed to giving full credit where it is due. This is an interesting research article and it helps to further highlight the novelty of our approach compared to others in the field. Our work is wholly distinct from this report on many levels, in terms of the fundamental concept, the focus of the application and also in relation to the method of silica coating and testing/experimentation.

To give due credit to this interesting work and to clarify the difference, we have included an additional sentence in the ***Introduction***, referencing the study mentioned by the reviewer:

Introduction

… Our novel approach is quite distinct from a recent and interesting report that used a silica-resin coating technology for the sole purpose of imbuing surgical masks with antimicrobial agents [12].

Reviewer #2 (Remarks to the Author):

Dear authors, congratulations on the manuscript you have produced. It will undoubtedly help in the fight against COVID-19 (and similar ones).

Response:

We sincerely thank the reviewer for their kind and encouraging remarks.

Here are some recommendations for the text:

Abstract

Line 24, please correct the word commonplace.

Response:

We thank the reviewer for pointing this out. The revised text has now been corrected:

The global demand for personal protective equipment has at times led to shortages of face masks for the public, therefore makeshift masks have become commonplace.

Line 24,25: "Severe acute respiratory syndrome coronavirus 2 (SARS-CoV25 2) has a spherical particle size of ~97 nm". It might be better to write: "The severe acute respiratory syndrome caused by coronavirus 2 (SARS-CoV-2), which has a spherical particle size of ~97 nm". We are talking about the size of the virus and not the respiratory syndrome.

Response:

We thank the reviewer for pointing this out. The revised text has now been corrected:

The severe acute respiratory syndrome caused by coronavirus-2 (SARS-CoV-2) has a spherical particle size of ~97 nm.

Line 30: Change "fibres" to fibers.

Response:

We sincerely thank the reviewer for their diligence. However, our article is written using British English. This is in accordance with the journal guidelines (as there is no language requirement to be American or British English). For this reason and to be consistent to the language used throughout the article, the original spelling is kept unchanged.

Lines 31,32: Change "aerosolised" to aerosolized.

Response:

We thank the reviewer for this suggestion. However, as above, we have made no changes in this regard.

Figure Graphical abstract: please indicate the size of the particles on the figure.

Response:

We thank the reviewer for this suggestion. The graphical abstract has now been updated indicating the diameter of the silica particles:

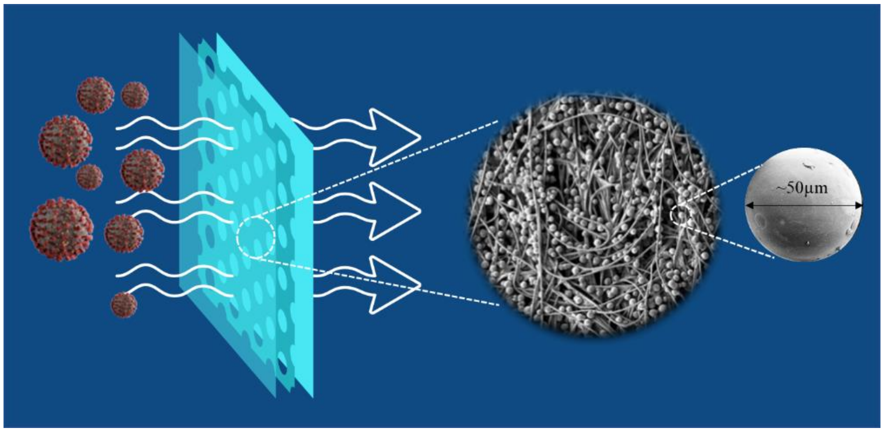

Introduction

Line 51, The particles produced by coughing have a typical size range of ~0.6-500 µm13. It differs from what you told in the abstract (typically>600nm). Please correct the abstract. ~0.6-500 µm is the correct information.

Response:
We thank the reviewer for pointing this out. The revised text has now been corrected accordingly (in the Abstract):

> *However, the airborne transmission of this virus requires the expulsion of droplets, typically ~0.6-500 µm in diameter (by coughing, sneezing, breathing, and talking).*

Line53, correct spelling for aerosolized

Response:
We thank the reviewer for this suggestion. However, we are using British English and therefore no change has been made in this regard.

Line 54, correct spelling for defenses

Response:
We thank the reviewer for this suggestion. However, British English spelling is retained.

Line 60,61: "it has been shown by Hazard et al that the inclusion of a valve reduces filter efficiency". This type of mask only protects the virus from entering the individual and does not prevent its spreading. If you want, you can add this information.

Response:
We agree with the reviewer. This point has been rephrased and added into the introduction section in the revised version:

> *Some N95 respirators include a one-way valve to increase the breathability, however, it was demonstrated by Hazard et al. that the inclusion of a valve reduces filter efficiency relative to a valve-less N95[13]. Of course, this type of mask only filters air breathed in, and does not reduce virus transmission from an infected wearer to others.*

Line 94,95,96. Please correct the words patchwise, hypothesized, and aerosolized.

Response:

We thank the reviewer for these suggestions. The revised text has been corrected to "patch-wise" but "hypothesised" and "*aerosolised*" remain unchanged due to the use of British English.

In general, proteins are strongly adsorbed to hydrophobic and hydrophilic interfaces by electrostatic interactions due to the patch-wise hydrophobic/hydrophilic characteristics of their three-dimensional surface

Line 98. The surface properties of amorphous silica depend.

Response:

We thank the reviewer for pointing this out. This has been corrected:

The surface properties of amorphous silica depend on the presence of surface hydroxyl groups…

Line 111, correct spelling for idealize

Response:

We thank the reviewer for this suggestion. However, British English spelling is retained.

Line 115, fiber

Response:

We thank the reviewer for this suggestion. However, for reasons noted earlier, British English spelling is retained.

Line 120, minimize

Response:

We thank the reviewer for this suggestion. However, British English spelling is retained.

Lines 122-123, aerosolized, characterized, idealized. The text is full of spelling errors. Please do a general spell check. From now on, I will no longer point out this type of mistake.

Response:

We sincerely thank the reviewer for their suggestions and diligence in checking our work. However, as noted earlier, in line with the journal guidelines British English spelling is used throughout the manuscript. Thus, no changes have been made in relation to this comment.

Results and Discussion
Selection of particle size and porosity of amorphous silica

Line 133-137: "Although smaller silica particle sizes (< 60 µm in diameter) can increase the overall surface area available and can likely improve capture efficiency of aerosolized proteins and virus, they may increase the risk of inhalation of silica dust".

How to ensure that the user does not inhale silica particles (or fragments)? Was any testing done? Inhaling these particles would be a disaster.

Response:
We understand the reviewer's concern.

However, in this project, we used amorphous silica. It is well-known that crystalline silica dust exposure can lead to silicosis[9]. Whereas, according to a report by the US Agency for Toxic Substances and Disease Registry[10], studies of amorphous silica in workers and laboratory animals have not found any evidence of cancer. There are no known health effects from exposure to amorphous silica at the levels found in the environment or in commercial products.

As noted in the original submission, all activities have been carried out in a controlled environment without exposure to any human subjects. Furthermore, as a precaution, we used a fusible material to keep amorphous silica trapped preventing release of any particles. Additional information relating to safety has been included in the revised manuscript as follows:

> ***Selection of particle size and silica form***
>
> *General design criteria are based on inhalation safety, filtration effectiveness and breathability. It is well-known that repeated exposure to crystalline silica dust can lead to silicosis, which can be fatal [9]. However, in this project, we use amorphous silica. Based on a report from the US Agency for Toxic Substances and Disease Registry [10], studies of amorphous silica in workers and laboratory animals have not found evidence of cancer. There are no known health effects from exposure to amorphous silica at the levels found in the environment or in commercial products.*

Filter efficiency of silica-coated cotton material for common aerosolized proteins (suggestion for subtitle)

Response:
We thank the reviewer for this suggestion. We agree and the revised subtitle has now been updated as suggested.

Filter efficiency of silica-coated cotton material for aerosolized inactivated SARS-CoV-2 proteins (suggestion for subtitle)

Response:

We thank the reviewer for this suggestion. We agree and the revised subtitle has now been updated as suggested.

Both these tests and results were fascinating!

Filter efficiency of silica-coated cotton material vs commercially available cotton face covering
Line 218, Did you mean table 1 and not table 2

Response:
We thank the reviewer for pointing this out. It has been corrected to Table 1.

As summarised in Table 1, the filter efficiency of the silica-coated material significantly outperforms the common cotton face covering for the aerosolised proteins and aerosolised inactivated SARS-CoV-2 tested.

'Breathability' testing
Line 227, Why use an airflow rate of 85 L/min? Is this flow close to that of human breathing? How much is it? Explain

Response:
An airflow rate of 85 L/min was used based on a common standard test procedure (TEB-APR-STP-0007) issued by the United States National Institute for Occupational Safety & Health (NIOSH)[14]. We noted and referenced this standard in the original submission in relation to the measured flow rate of 85 L/min (**Materials and Methods, Breathability test**).

This procedure is widely used to determine the inhalation resistance of air-purifying respirators at a standardised constant flow of 85 L/min, and has been commonly employed in research studies to assess respirator breathability [15,16]. The minute ventilation rate of a normal adult male at complete rest is approximately 6 L of air/min, which can increases up to 16, 40, 140 L of air /min during normal activity, moderate exercise and a stress test, respectively[17]. The 85 L/min air flow rate used in this procedure is equivalent to pulmonary ventilation during heavy exercise[18].

Conclusion
Please, speak in terms of the percentage how much your method can increase the filtering efficiency of the masks.

Response:
We agree and thank the reviewer for this suggestion. The conclusion has been updated accordingly:

In this feasibility study, the well-known affinity of silica for some common proteins has been demonstrated in an aerosolised form showing filter efficiencies of 97%, 87%, 91 % and 95% for aerosolised myoglobin, BSA, ubiquitin and cytochrome c, respectively – with an average increase in filter efficiency of ~17% compared to an untreated blank. The addition of amorphous silica to a swab was

also shown to decrease the amount of aerosolised SARS CoV-2 that passed through the face covering by ~65%.

Filter efficiency test

When Reading, I wish the method were better explained in detail, explaining figures 6 and 7 in this part of the text.

Response:

We thank the reviewer for this suggestion. Whilst fig. 6 and 7 are explained in detail later in this section, we tend to agree that a brief explanation at this juncture can help to improve the readability. Therefore, we have added some additional sentences, to this effect in the revised manuscript (in the filter efficiency test section within Materials and Methods):

The apparatus and workflow are shown in Figures 6a and 7, respectively. Briefly, the test setup draws in air along with the nebuliser output at a given flow rate which passes through the material under test (i.e., the filter material), which is clamped in position (Fig. 6a). A portion of the air is sampled both before and after the material under test, and subsequently measured to determine the filter efficiency. Two measurement methods were carried out: mass spectrometry for the four common proteins and lateral flow assay for inactivated SARS-CoV-2 (Fig. 7).

I want to congratulate you on the work you have done. I understand that this method can be fascinating, and the result has outstanding figures.

But I missed a few things in the text:

1) Can they be washed when these masks are submitted to the process with silica?

Response:

The reviewer asks an interesting question regarding the potential to reuse the silica coated material. While it is possible to regenerate silica (just like a chromatography column using a proper mobile phase), it should be noted that this study is early stage research focused on demonstrating that silica can effectively capture aerosolised proteins and virus. The efficacy of the filter in real-world scenarios and its potential for reuse would require extensive testing and validation, which are beyond the scope of this present research study.

2) Do these mascaras degrade over time? Hours, days?

Response:

The reviewer asks an interesting question regarding the stability of the silica. To address this concern, we conducted an experiment where we left three silica swabs under ambient storage conditions for one month. Afterwards, we tested their filter efficiency with aerosolised proteins, following the same method as described for freshly prepared samples. The results showed that there was no significant increase or decrease in the filter efficiency of aerosolised proteins compared to the freshly prepared samples (Figure S3), indicating that the silica-coated covering have good stability and useable lifetime. We have included this observation in the manuscript under "***Morphology and stability of silica-coated substrate***" section:

Morphology and stability of silica-bonded cotton

Results from a preliminary stability test indicate that the silica swabs stored under ambient conditions for one month exhibited no significant difference in filter efficiency of aerosolised proteins, compared to freshly prepared swabs (Figure S3, p > 0.05). This suggests that the silica swabs remain stable and useable for at least one month.

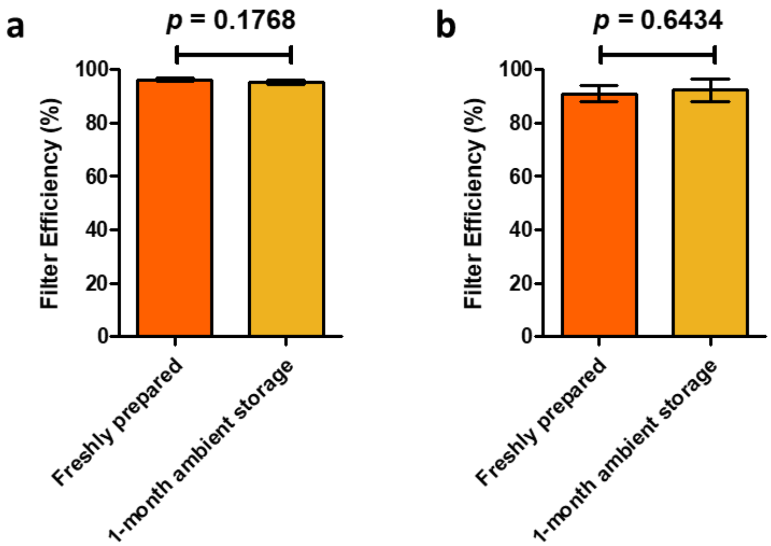

Supplemental Figure S3. *Storage stability of silica-coated swabs in relation to their filter efficiency for aerosolised proteins. a. myoglobin, and, b. ubiquitin. The silica-coated cotton materials were stored under ambient conditions for one month before undertaking a filter efficiency test and compared against freshly prepared silica swabs.*

3) Is there any risk of inhaling silica?

Response:

There was no risk of silica inhalation in this study. As noted in an earlier response, it is important to note that this study does not use crystalline silica. We use amorphous silica which has no known health effects at levels found in the environment or in commercial products.

In any case, in this study we took several measures to prevent the release of silica particles including the use of a fusible material to ensure any loose particles are retained, and all testing was carried out in a controlled environment.

4) How to compare the results with other mascaras on the market and other studies? Use the quality factor described by Zangmeister et al. 2020 and World Health Organization (WHO)

Response:

We thank the reviewer for this suggestion, and we have included the quality factor metric in the revised manuscript and cited appropriate references accordingly [19, 20]. The calculation method is described in the ***Filter quality factor calculation*** section within

Materials and Methods, and the result is added in **Filter quality factor** within the **Results and discussion** section and also contained within the table portion of Figure 5b.

Materials and Methods:

Filter quality factor calculation

The filter quality factor (QF) is a function of filtration efficiency and breathability [20] and calculated as follows:

$$QF = -\frac{log(1-FE/100)}{\Delta P} \qquad (2)$$

where FE is the filter efficiency (%) and ΔP is the pressure drop (kPa).

Result and discussion

Filter quality factor

The filter quality factor (QF) is a relative indicator to assess the overall performance of an air filter (see Equation 2, Materials and Methods). For testing at similar flow rates, a higher QF value indicates better overall efficiency, which is a result of both higher filtration efficiency and lower pressure drop. According to expert consensus reported in WHO guidance [19], a $QF \geq 3$ is the minimum value recommended for cloth masks. In comparison, the silica-coated substrate had an almost two-fold increase in QF compared to the blank control (8.6 vs 4.6 kPa^{-1}, Figure 5b). The QF for the commercial cotton face covering was below the WHO recommended level (1.7 kPa^{-1}), suggesting that it may not provide sufficient filtration efficiency.

a

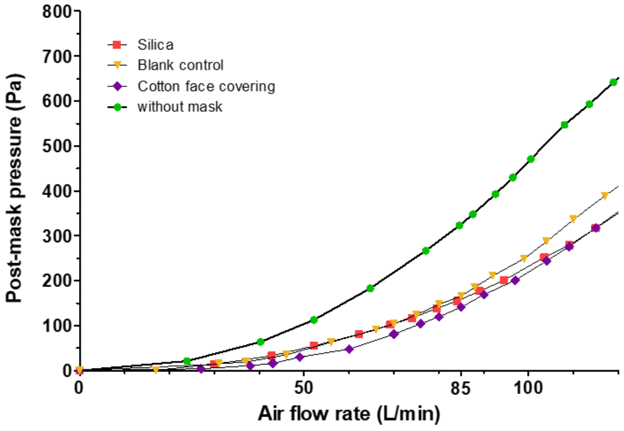

b

Face Covering Material	Pressure drop compared to the unobstructed response at an air flow rate of 85 L/min (Pa)	Filter quality factor (kPa^{-1})
**Silica**	152 (22 Pa/cm^2)	8.6
**Blank Control**	143 (20 Pa/cm^2)	4.6
**Cotton Face Covering**	166 (24 Pa/cm^2)	1.7

Figure 5. Breathability test. ***a****. Curves of post-mask measured air pressure against air flow rate.* ***b****. Pressure drops of face covering materials compared to the unobstructed response at an air flow rate of 85 L/min (Pa) (n = 3) and filter quality factor (kPa^{-1}).*

5) Need to improve the language (English) of all text. It is full of orthographic errors.

Response:

We do apologise for orthographic errors and we want to thank the reviewer for pointing several of them out. We have gone through the whole text to check the spelling, punctuation and grammar and have undertaken several minor corrections in this regard.

Reviewer #3 (Remarks to the Author):

This manuscript evaluates the effectiveness of a novel, silca-functionalized, cotton filter material at capturing biological aerosol particles.
Upon review of this manuscript, I find that the evaluation of the functionalized cotton filters is intriguing, but does not demonstrate, the mechanism of removal described. The primary issues that I have with this manuscript are the methods used to derive the data and the interpretation of the data, due primarily to the limitations in the methods.

Q1 The most significant methodological issue is the **lack of aerosol size distributions**.

Response:

We thank the reviewer for pointing this out. We tend to agree. We have sought to address this and have added a new section to the **Results and Discussion** section entitled "***Size distribution of bioaerosols***". The aerosol size distribution of the nebuliser can be found in Figure S5 in the supplementary materials:

Size distribution of bioaerosols

An Orthorex mini ultrasonic nebuliser was used to generate aerosols from the test working solutions and used for all filter efficiency tests unless stated otherwise. The particle size distribution of the aerosol was measured for several test solutions, including pure water, cytochrome c (1 mg/ml in water), BSA (1 mg/ml in water), inactivated SARS-CoV-2 (1.3 x 10^6 PFU/mL), creatinine (1 mg/ml in water) and caffeine (1 mg/ml in water). The results showed that all solutions had very similar distributions, as shown in Figure S4. The most frequent particle diameter band for all test solutions was between 0.3-0.4µm. Characterisation of the nebuliser showed that most particles were smaller than 1 µm (Figure S4), and thus can be considered to be aerosolised, which is consistent with the data reported by the manufacturer [21-23]. The refractive indexes of all testing materials were very similar to that of pure water (Table S3), indicating that the laser light scattering for particle size distribution measurements was not affected by the internal properties of the aerosols.

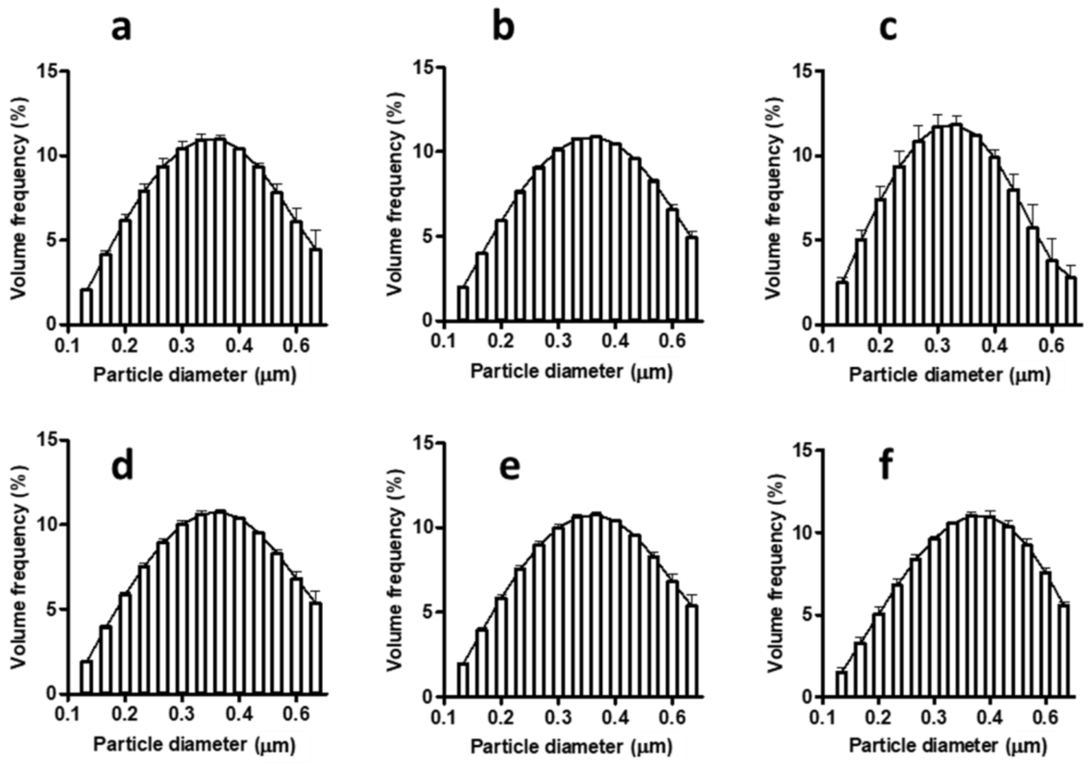

Supplemental Figure S4. Particle size distribution of aqueous aerosol containing 1mg/mL of a, pure water, b, cytochrome-c, c, BSA, d, inactivated SARS-CoV-2, e, creatinine, and, f, caffeine generated by the nebuliser (ORTOREX™ Portable Ultrasonic Nebuliser), measured by Malvern Panalytical Spraytec laser diffraction system (Malvern, UK) (n = 3).

Q2 The authors present a characterization of the nebulizer, but do not say material was nebulized, nor at what concentration.

Response:

We thank the reviewer for pointing this out and sincerely apologise about the missing information. Pure water was used to dissolve all of the materials. Furthermore, we have updated the aerosol characterisation data to include additional materials (Figure S4) and have also added further details to the ***Bioaerosol generation method*** section (within Materials and Methods), as copied below:

Bioaerosol generation method

… The test solutions were nebulised by an Orthorex mini ultrasonic nebuliser. The emitted aerosol particle size distributions were characterised using a Malvern Panalytical Spraytec laser diffraction system for several test solutions (Figure S4) including: pure water, cytochrome c (1 mg/ml in water), BSA (1 mg/ml in water), inactivated SARS-CoV-2 (1.3 x 10^6 PFU/mL), creatinine (1 mg/ml in water) and caffeine (1 mg/ml in water)…

Q3 Further, the methods only **outline the concentration** of non-protein materials that were nebulized, and not the proteins. These should be presented.

Response:

We thank the reviewer for pointing this out. We agree, this could have been made clearer. We have included a new section ***Preparation of aerosol stock solution*** within Materials and Methods to present how the working protein solutions were prepared including their concentrations from the stock solution.

> ***Preparation of aerosol stock and working solution***
> *1 mg of each standard material (cytochrome c, myoglobin, BSA, ubiquitin, caffeine and creatinine) was weighed and dissolved in 10 mL water separately to make a standard working solution at 1 mg/mL. It was directly used for nebulisation without further dilution.*
>
> *For inactivated SARS CoV-2, a stock buffer solution of DMEM containing 1.3 x 10^7 PFU/mL of inactivated SARS CoV-2 was diluted 10 times with water (1: 9, v/v) before use. The working solution was approximately at 1.3 x 10^6 PFU/mL concentration.*
>
> *Each working solution was freshly prepared each day before analysis.*

Q4 Further, **size distributions of each material** should be presented. Changes in aerosol size can have significant impacts on filtration efficiency. The data presented here, do not conclusively demonstrate that the filtration of protein containing particles is enhanced over non-protein containing particles. They only indicate that the filtration efficiency for the different experiments is different, without conclusively identifying a mechanism. Therefore, the claim that the filter enhances the biological filtration through protein capture is not demonstrated. The authors need to present size distribution data for each material,

Response:

We have updated the supplemental materials to include Supplemental Figure S4, which shows the particle size distribution for six test materials. The size distributions are very similar showing the most frequent band of particle diameter for all materials tested was between 0.3-0.4 µm. Additionally, we measured the refractive indexes of all materials (Table S3), which were very similar to that of pure water. This indicates that the laser light scatting used for particle size distribution measurement is not affected by the material internally. Since the aerosolised input for each test is consistent (and virtually the same in terms of size distribution) then the filtration efficiency claims are valid relative to the non-silica coated blank.

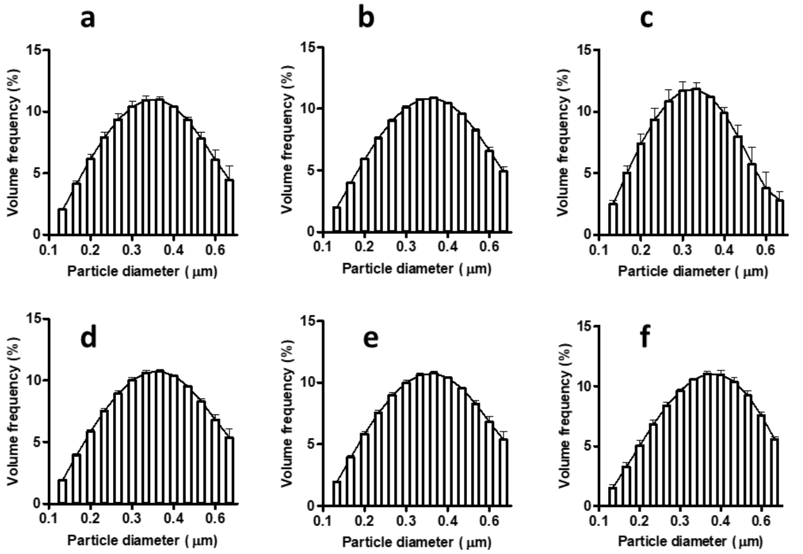

Supplemental Figure S4. Particle size distribution of aqueous aerosol containing 1mg/mL of a, pure water, b, cytochrome-c, c, BSA, d, inactivated SARS-CoV-2, e, creatinine, and, f, caffeine generated by the nebuliser (ORTOREX™ Portable Ultrasonic Nebuliser), measured by Malvern Panalytical Spraytec laser diffraction system (Malvern, UK) (n = 3).

Supplemental Table S3. Refractive index of aqueous solutions of: pure water, proteins (1 mg/mL in water), buffer solution (inactivated SARS-CoV-2), small molecules (1 mg/mL in water) and ethanol (used as a positive control).

	**Refractive index**	
**Wavelength**	**589.3 nm**	**633 nm**
**100% water (blank control)**	1.3279	1.3229
**Bovine serum albumin**	1.3280	1.3230
**Ubiquitin**	1.3280	1.3229
**Cytochrome c**	1.3280	1.3231
**Myoglobin**	1.3280	1.3230
**Buffer solution (inactivated SARS-CoV-2)**	1.3280	1.3231
**Creatinine**	1.3280	1.3249
**Caffeine**	1.3280	1.3230
**Ethanol (positive control)**	1.3540	1.3488

Room temperature: 25.1^{o}C.

Q5 and the **complete description of the aerosol stock solution**, including both the concentration of target material (protein etc.), and the diluent used in order to allow for evaluation of the claims. Without this data, it is impossible to separate the physical filtration efficiency from the potential enhancements due to selective biological filtration efficiency.

Response:

We have included a new section "***Preparation of aerosol stock solution***" within Materials and Methods in the revised manuscript to clearly describe the aerosol stock preparation procedure, including the concentration and dilution:

> ***Preparation of aerosol stock and working solution***
> *1 mg of each standard material (cytochrome c, myoglobin, BSA, ubiquitin, caffeine and creatinine) was weighed and dissolved in 10 mL water separately to make a standard working solution at 1 mg/mL. It was directly used for nebulization without further dilution.*
> *For inactivated SARS CoV-2, a stock buffer solution of DMEM containing 1.3 x 10^7 PFU/mL of inactivated SARS CoV-2 was diluted 10 times with water (1: 9, v/v) before use. The working solution was approximately at 1.3 x 10^6 PFU/mL concentration.*

Q6 Finally, there is a logical flaw in the description of the potential enhancement to filtration efficiency, as it is described. In order to for any filter (functionalized or not) to capture a particle it must impact the surfaces of the filter, in this case the filter fibers. Therefore, any chemical enhancements to filtration would actually be in the retention of particles on the fibers through adsorption, and if the action of the functionalized filters were purely chemical, that would indicate that non-functionalized filters collected particles at the same rate as the functionalized filters, but if the filter were non-functionalized, some fraction of the particles wouldn't "stick" to the filter fibers. Based on the SEM image presented in Figure 1, it seems to me that the **dramatic increase in surface area** resulting from **functionalization with silica results** in increased capture efficiency, rather than increased retention efficiency.

All that said, I do believe that this filter material results in a significant advance in filtration technology. I simply think that it is a result of **enhanced physical filtration efficiency**, rather than an enhancement in **biological retention efficiency**. I admit that the data don't clearly indicate which mechanism is dominating. In order to clearly identify the **true mechanism more work** is required:

Response:

The reviewer makes some very interesting points, which we really appreciate. In the literature it is well-known that capture is either by physical means (e.g., direct interception, inertial impaction, diffusional deposition, gravitational settling) or via electrical forces. One can view much of nature as interactions due to electrical forces. In some regards, the interaction of silica with protein can be considered electrical (which is perhaps a more fundamental description of chemical interactions). However, in practice, for any filter, an exact description of the filtration mechanism is difficult, because it is likely that several mechanisms will be acting together and to varying degrees. Nevertheless, in order to shed more light regarding the mechanism we have developed a new experiment.

Before discussing the new experiment and the results thereof, further discussion is required regarding some of the other the interesting points raised. The reviewer correctly points out that the addition of the silica particles, should increase the available surface area for physical interaction. We completely agree with the reviewer. If this were the preeminent mechanism (which we don't think it is), this would still represent quite a remarkable result, given the negligible impact upon breathability (pressure drop).

The reviewer also brings up another interesting point for discussion, regarding retention efficiency, noting that "[the] dramatic increase in surface area resulting from functionalization with silica results in increased capture efficiency, rather than increased retention efficiency". This seems a logical deduction, and we are somewhat in agreement – although they are not mutually exclusive. We also suspect the silica to have an increased retention efficiency and that both features are at play in concert. We have performed an experiment to shed some light on this too.

The new experiments have been devised to give some fundamental empirical insight to the underpinning mechanisms at play. We have designed and fabricated a substrate-less (or fibreless) test rig that uses woven stainless-steel mesh to physically retain the silica. Aerosolised protein is passed at a constant rate for a set time period through the mesh which contains the silica in question. Upon completion, the silica under test can be simply released from the mesh and collected for further analysis (i.e., any material that is retained by the silica can then be eluted and measured).

A critical feature of these experiments is how the silica was treated (for one of the cases tested). The experiment examines the capture of aerosolised BSA by silica (i.e., the silica used in this study) versus an identical silica but heat treated. The heat treatment is an important distinction as it leads to the loss of surface activity on the silica (as noted in the revised manuscript, with supporting references, and as evidenced by zeta potential measurements recorded in Table S2). Thus, there are two silica 'variants' that are tested. They can be considered identical in terms of their physical size and shape – except the surface chemistries are distinctly different.

The results can be seen in Fig. S8, where there is a stark contrast between the BSA signals measured; the protein signal eluted from the heat-treated silica is barely visible in comparison, approximately an order of magnitude less intense. This demonstrates that the surface activity of the silica is very important for aerosol capture (and not just its surface area), and demonstrates that the capture mechanism is not predominantly physical. Furthermore, elution was carried out with an organic solvent highlighting the ability of silica to retain protein that is captured.

In the revised manuscript we have included a new section titled ***Mechanistic considerations for silica-based capture/adsorption of aerosolised protein*** within Results and Discussion, along with a new corresponding section titled ***Silica capture mechanism experiments*** in Materials and Methods:

Mechanistic considerations for silica-based capture/adsorption of aerosolised protein

Air filtration capture mechanisms can broadly be thought of as physical or electrical. Physical capture mechanisms take place without the influence of attractive forces (e.g., direct interception, inertial impaction, diffusional deposition, gravitational settling, etc.). Whereas electrical capture mechanisms involve electrical forces between the airborne particles and the filtration material. In reality, for any filter, an exact description of the filtration mechanism is difficult, because it is likely that several mechanisms will be acting together and to varying degrees.

In the case of the silica filter developed herein, it is clear that relative to a blank substrate (i.e., identical but without silica) the surface area available for physical capture has increased, which can increase the likelihood of physical interaction. In order to shed some light on the mechanism of capture by the silica and indeed our original hypothesis, we devised an experiment that uses only silica, without having to attach it to a substrate (Supplemental Figure S8a). A key aim for this experiment, is to be able to decipher the predominant mechanism that is responsible for the capture of aerosolised proteins by silica. Therefore, we tested two variants of the silica: i) the QA-functionalised silica that has been used for all other tests in this study which exhibits a markedly increased FE for aerosolised proteins, and ii) a heat-treated (800ºC) version of the same QA-functionalised silica. Performing a heat treatment effectively removes silanols from the surface (Table S2), yielding silica particles that are essentially identical in terms of their physical size and shape, yet with distinctly different surface chemistries. As part of the experiment, aqueous protein solution is nebulised and passes through the silica particles held within a stainless-steel mesh support. Afterwards, protein is eluted from the silica prior to direct MS analysis. Further details of the test setup and analysis procedure are included in the materials and methods section and Figure S8.

Our experiment revealed that the aerosolised protein capture efficiency of silica was about ten times higher than that of the heat-treated version (Supplemental Figure S8b), clearly demonstrating that the predominant mechanism of interaction is not physical. Or at least one can say that the surface activity of the silica is vital, as in reality we expect that the increased surface area and surface chemistry work together in concert. This result agrees with our original hypothesis, that the surface chemistry of the microporous functionalised silica is crucial in capturing protein. Zeta potential measurements also corroborate this result showing a reduced surface charge of the heat-treated silica as expected (Table S2).

Furthermore, this experiment sheds some light on the retention efficiency. We anticipate that the QA-functionalised silica can interact with polar groups present in proteins, such as amine and carboxylic acid groups, through hydrogen bonding and various types of electrostatic interactions. For this experiment, an organic solvent was used to elute (i.e., release) the protein from the silica for subsequent measurement. This indicates that bioaerosols captured by the silica are also likely to be retained by it. Finally, for comparison, we performed a similar experiment,

except we exposed both silica variants by mixing them with protein in the liquid phase, which yielded a similar result (Figure S8c).

Materials and Methods

Silica capture mechanism experiments

In order to assess the mechanism by which silica captures aerosolised protein and also to explore its retention efficiency, we developed a fibreless nebulising system (Supplemental Figure S8a). The main part of the chamber was fabricated using photoresin with a 3D printer (Formlabs, UK). This section incorporates a stainless-steel mesh support and an exhaust directly below it. A polycarbonate enclosure (RS components, UK) was modified to connect to the 3D printed part at one end and the nebuliser at the other, to remove any larger droplets emitted from the nebuliser. Aerosolised protein that passes in to the 3D printed chamber is able to escape via an exhaust mounted in the base, and is actively sampled by a vacuum pump (flow restricted to 10 L/min) connected at the top of the chamber. A removable piece contains two fixed woven stainless steel meshes and another woven stainless-steel mesh that is removable (MeshDirect, UK). The central mesh (mesh 28) has an aperture of 0.55 mm. This is used to provide an even spacing for the silica when it held in place for testing. Below this is a fixed 0.055 mm aperture (mesh 300), and above is another identical mesh which is detachable. The silica is applied evenly with the detachable mesh removed. After application it can be placed on top and is held in place by four screws. This entire piece can be removed allowing it to be readily washed, weighed and also dried in an oven post-nebulisation (and prior to removing exposed silica, which is best removed when dry). The nebuliser used for this particular experiment was a compressor type Omron C28P (Omron Healthcare, UK).

Silica was tested in two variants: i) in its regular form as utilised in all other tests in this study, and, ii) heat-treated (800ºC) to remove silanols from the surface. Both were tested using identical conditions.

The capture mechanism experiment exposed 0.2 g of each silica variant, held within a stainless-steel mesh, to nebulised BSA, 1 mg/mL dissolved in water. During the experiment, the protein solution was nebulised at a flow rate of 0.4 mL/min for 5 minutes, afterwards the silica was sufficiently dried in an oven and then collected from the mesh support. Both silica samples were then washed three times with water using vigorous vortexing, prior to elution of the protein from the silica using 100% methanol. The resulting extract was directly injected into the mass spectrometer for analysis.

In relation to retention efficiency, acting as a control of sorts, each silica was incubated with protein in solution phase. The protein used for this test was also BSA at a concentration of 0.01 mg/mL dissolved in water, which was incubated with 0.05g of each silica for one hour before MS analysis.

Supplementary

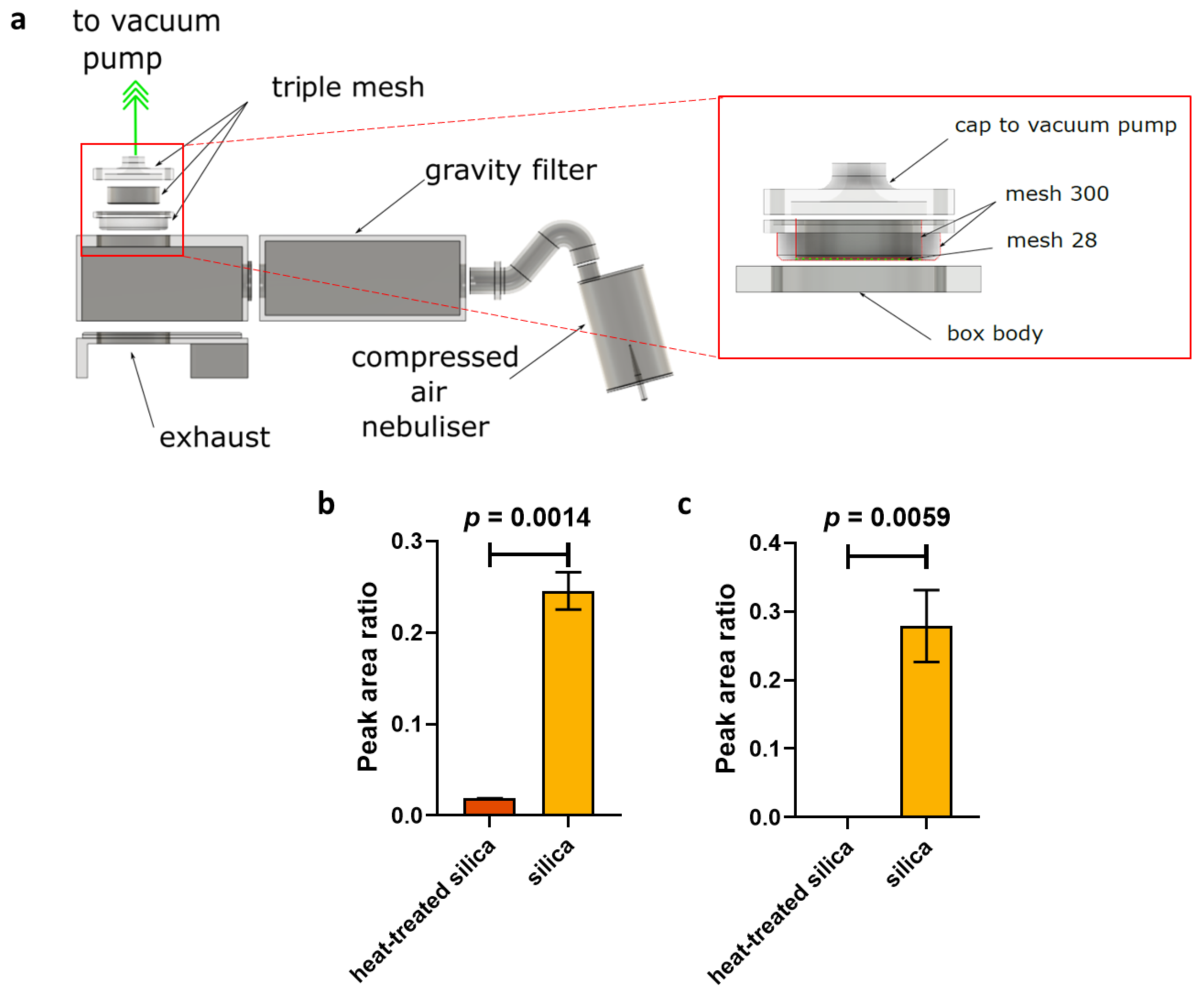

Supplemental Figure S8. The capture efficiency of functionalised silica was tested in a fibreless setup (without attaching silica to a fabric substrate) to shed light on the underpinning mechanism responsible for aerosolised protein capture. a. Schematic illustration of the fibreless test apparatus. The triple mesh is made from woven stainless steel and holds the unbound silica particles in place. Mesh 300 is a woven stainless-steel mesh with an aperture of 0.055 mm, and mesh 28 is a woven stainless-steel mesh with an aperture of 0.55 mm; refer to Materials and Methods for further details. Silica is held in the mesh support and is exposed to nebulised bovine serum albumin (BSA). b. The signal intensity of BSA eluted from the two silica variants after exposure to aerosolised BSA (conc. 1 mg/ml in water). c. The signal intensity of BSA eluted from the two silica variants after being exposed to BSA (conc. 0.01 mg/mL) in solution phase.

Q6.1. A size-resolved physical filtration measurement is necessary. There are several standard methods that could be leveraged.

Q6.2. Size-resolved filtration efficiency measurements for each material evaluated.
Q6.3. Comparison of 1 and 2 will clearly identify the impact of particle composition on filtration efficiency and more clearly identify the mechanism.

Response:

We thank the reviewer for these suggestions. We have sought to "more clearly identify the mechanism" by means of the fibreless experiment noted in the previous response. This clearly demonstrates that the preeminent mechanism is not physical (i.e., due to increased surface area alone) and the surface activity of the silica is crucial in this regard. Therefore, size-resolved filtration measurements are not necessary. In any case, we have performed some size-resolved measurements characterising the nebuliser output (for six solutions tested) showing that the size distribution of the nebulised material is highly consistent and approximately the same, irrespective of the solution make up. Thus, the testing is fair and the conclusions valid. Unfortunately, however, the aerosol sizing equipment is designed as an optical bench arrangement and is not suitable for sampling/measuring the post-filter size distribution from the FE test rig. Whilst it would be desirable to have this data, as noted, it is not essential.

Line 53 *Droplets smaller than 5 μm can become aerosolised and travel much larger distances.*

This can apply to significantly larger droplets, as well. I'd like to see this rewritten in a way that doesn't lean on the 5 micron cutoff for airborne transmission, which has been demonstrated to be based on false assumptions.

Response:

We agree and thank the reviewer for this comment. This has been rewritten as,

> *Droplets of all sizes can become aerosolised and travel long distances, but smaller particles, including those smaller than 5 μm, are more likely to remain suspended in the air and travel farther.*

Line 54 *One of the best defences against spreading SARS-CoV-2 infected droplets is mask wearing.*

This should be particles. This paragraph is written as particles being the general description of all sizes between 0.6 and 500 microns, with droplets being larger particles, and aerosols being smaller ones. In general, I prefer the nomenclature of droplets being the initially emitted wet particles, and either particles or aerosol particles referring to the dried material.

Response:

We agree and thank the reviewer for pointing this out. This has been corrected in the revised manuscript.

Line 76 ….. *A number of reports have used novel topologies including pathogen-inactivated….*

pathogen-inactivating or antimicrobial

Response:

We thank the reviewer for this comment and have performed this correction.

Lin 96 …*Thus, we hypothesised that amorphous microporous silica could be used to efficiently capture aerosolised proteins in general and, specifically, SARS CoV-2 by virtue of its protruding spike proteins.*

this certainly applies to a wide variety of viruses, bacteria and other microorganisms with antigenic proteins. I would say, however, that in the majority of human-generated bioaerosols, human respiratory material likely represents a large fraction of the surface material in the bioaerosol, which certainly contains human proteins. This should be reworded to better reflect the realities of naturally occurring and human emitted bioaerosol.

Response:

We tend to agree and thank the reviewer for this comment. We have adjusted the emphasis of this sentence to make this statement more generic.

> *… we hypothesised that amorphous microporous silica could be used to efficiently capture aerosolised proteins, and more generally bioaerosols, including SARS-CoV-2 by virtue of its protruding spike proteins.*

Furthermore, we have adjusted the title of the manuscript to reflect this also, by the addition of two words, "Bioaerosols including",

> *Corona Capture: Attaching Protein Adsorbing Silica Particles to the Surface of Cotton Substrates for the Capture of Bioaerosols including SARS-CoV-2*

Line 107 *In this article, we propose a novel approach for the capture of bioaerosols, particularly airborne viruses such as SARS-CoV-2, based on the principle of adsorption.*

Again, see above, I don't think this "particularly" applies to viruses. I think it pretty generally applies to all bioaerosols.

Response:

We agree and thank the reviewer for this comment. We have updated the sentence to include all bioaerosols.

> *In this article, we propose a novel approach for the capture of bioaerosols, including airborne viruses such as SARS-CoV-2, based on the principle of adsorption.*

Line 148 *This is an important consideration since viral particles are carried in exhaled droplets.*

droplets and the resulting dry particles

Response:

We appreciate the reviewer's suggested correction which we have implemented in the revised manuscript.

Line *167 If this could be demonstrated for aerosols, then it would open the possibility of capturing the SARS CoV-2 virus by virtue of spike proteins covering its outer lipid membrane.*

again, I don't think this is what is happening. This should be generalized to say "by virtue of the surface proteins on the particle" or something similar.

Response:

We thank the reviewer for this suggestion, which we agree with.

If this could be demonstrated for aerosols, then it would open the possibility of capturing the SARS-CoV-2 virus by virtue of the surface proteins on the particle.

Line 174 *The filter efficiencies of the silica coated cotton for aerosolised myoglobin, BSA, ubiquitin and cytochrome c were 97%, 87%, 91 % and 95%, respectively.*

What are the particle sizes? Did they change between materials?

Response:

As noted in a previous response we did not measure the particle size distribution post-filtration.

Lin 178 *these materials were also tested for filter efficiency with other small polar molecules dissolved in water (hydrophilic creatinine and slightly hydrophobic caffeine), whereby a relatively* lower increase in filter efficiency was observed (Supplementary Figure S1).

Again, particle size is not described anywhere in this comparison. If these materials had different particle sizes than the proteins, that alone could explain the difference in filtration efficiency.

Response:

For the reasons noted in an earlier response, we did not measure the particle size-distribution post-filtration. We did, however, measure the aerosol size distributions of the nebuliser, which were very similar, regardless of the nebuliser solution (Supplemental Figure S4). The comparatively higher FE for proteins (compared to creatinine and caffeine) is an indication of their well-known propensity to be adsorbed by silica, as well as their polymeric nature offering several points for interaction and electrostatic attraction.

Line 185 *The reason may be due to their different physical properties (Supplementary Table S1 and Figure 3) including electrostatic and hygroscopic interactions, amongst others.*

I agree that differences in physical properties of the mask material could account for the differences.

Response:

We have demonstrated this with the new experimental results (Figure S8), as explained in response to a previous comment (Q6).

Line 192 *The important observation is that the silica-coated cotton showed similar filter efficiencies (10% difference) for the four proteins (Figure 2), indicating that, in general, a high filter efficiency can be expected for aerosolised proteins.*

Or, particles of similar sizes. If this experiment was not controlled for particle size, these comparisons cannot be made.

Response:
We measured the particle size distribution of the nebuliser. This was done for several test solutions. The predominant particle diameter in each case was between 0.3-0.4 µm, with no significant variation between the different solutions (Supplemental Figure S4). Therefore, no changes have been made in relation to the sentence noted above as line 192.

Line 209 *the filter efficiency of the silica-coated face covering for aerosolised SARS CoV-2 was the highest (average = 94%), compared to 84% for the blank material, and 80% for the commercially available cotton face covering (Figure 4b).*

Again, this could also be due to the differences in the physical properties of the mask material, and not protein capture of by the silica.

Response: We have shown that the nebuliser size distribution is consistent among the different test solutions characterised (Figure S4) and the test is fair. Furthermore, we have demonstrated that the surface activity of the silica is crucial in capturing the bioaerosol by comparing to an identical silica in terms of size and shape but with markedly different surface chemistry (due to dehydroxylation resulting from heat treatment), showing an order of magnitude difference in capture efficiency for aerosolised BSA.

Line 231 *Interestingly, this suggests that a silica coating has the potential to be used in combination with other materials (including established face masks) to improve filter efficiency yet with nearly no impact on breathability/pressure drop.*

Absolutely!

Response:
We appreciate the reviewer's positive and encouraging response.

Line 254 *While this paper has focused on common face coverings, the utilisation of silica as a virion adsorbent shows excellent potential for use in face coverings, air filtration systems and even as a bioaerosol sampler.*

Fundamentally, the data demonstrates that the functionalized material has improved filtration properties, compared to unfunctionalized and standard cotton material. However, these data do not effectively demonstrate the claim that the increased filtration is due to retention of particles through protein adsorption. In order for that to be true, these particles would have to have been captured and then re-entrained in the control case, but captured and subsequently retained on the fibers in the silica case. I think it is far more likely that the data is demonstrating improved physical filtration efficiency of these materials. The difference could be clearly demonstrated if the particle size information was known for all the cases.

Response:

We thank the reviewer for their line of questioning, especially relating to the mechanism which has encouraged us to think more deeply about this aspect. We agree with the reviewer's assessment that, "the data demonstrates that functionalized material has improved filtration properties, compared to unfunctionalized…"

With regards to the reasoning "through protein adsorption", we have shown this in the revised manuscript, as discussed in response to an earlier comment (Q6) via the fiberless test setup (Figure S8 in the revised manuscript). By heat treating the silica, we were able to have a control material that is identical in terms of its physical size and shape, but wholly distinct in terms of its surface activity. Under identical test conditions, a relatively small amount of aerosolised protein was captured by the heat treated variant (~tenfold less).

<u>Other:</u>

We thank all of the referees for their efforts in reviewing our work and the insight provided, which we believe has improved the quality of the revised manuscript. We have also improved the presentation of the revised manuscript with additional minor edits.

All changes have been tracked in the revised manuscript.

References

1 Brunauer, S., Emmett, P. H. & Teller, E. Adsorption of Gases in Multimolecular Layers. *Journal of the American Chemical Society* **60**, 309-319, doi:10.1021/ja01269a023 (1938).

2 Barrett, E. P., Joyner, L. G. & Halenda, P. P. The Determination of Pore Volume and Area Distributions in Porous Substances. I. Computations from Nitrogen Isotherms. *Journal of the American Chemical Society* **73**, 373-380, doi:10.1021/ja01145a126 (1951).

3 Stöber, W., Fink, A. & Bohn, E. Controlled growth of monodisperse silica spheres in the micron size range. *Journal of Colloid and Interface Science* **26**, 62-69, doi:https://doi.org/10.1016/0021-9797(68)90272-5 (1968).

4 Abdelmouleh, M. *et al.* Modification of cellulosic fibres with functionalised silanes: development of surface properties. *International Journal of Adhesion and Adhesives* **24**, 43-54, doi:https://doi.org/10.1016/S0143-7496(03)00099-X (2004).

5 Koga, H., Kitaoka, T. & Isogai, A. Chemically-Modified Cellulose Paper as a Microstructured Catalytic Reactor. *Molecules* **20**, 1495-1508 (2015).

6 J. Lavoie, Y. Cloutier, J. Lara & Marchand, G. Guide on respiratory protection against bioaerosols. Recommendations on its selection and use. *IRSST*, 40 (2007).

7 Lundqvist, M., Sethson, I. & Jonsson, B.-H. Protein Adsorption onto Silica Nanoparticles: Conformational Changes Depend on the Particles' Curvature and the Protein Stability. *Langmuir* **20**, 10639-10647, doi:10.1021/la0484725 (2004).

8 Engelking, L. R. in *Textbook of Veterinary Physiological Chemistry (Third Edition)* (ed Larry R. Engelking) 7-11 (Academic Press, 2015).

9 Bang, K. M. *et al.* Silicosis mortality trends and new exposures to respirable crystalline silica - United States, 2001-2010. *MMWR Morb Mortal Wkly Rep* **64**, 117-120 (2015).

10 Disease, A. f. T. S. a. & Registry (ATSDR). (ed Public Health Services. Department of Health and Human Services) (Atlanta, GA, USA, 2019).

11 Tsutsumi-Arai, C. *et al.* Surface Functionalization of Non-Woven Fabrics Using a Novel Silica-Resin Coating Technology: Antiviral Treatment of Non-Woven Fabric Filters in Surgical Masks. *International Journal of Environmental Research and Public Health* **19** (2022).

12 Tsutsumi-Arai, C. *et al.* Surface Functionalization of Non-Woven Fabrics Using a Novel Silica-Resin Coating Technology: Antiviral Treatment of Non-Woven Fabric Filters in Surgical Masks. *International Journal of Environmental Research and Public Health* **19**, 3639 (2022).

13 Hazard, J. M. & Cappa, C. D. Performance of Valved Respirators to Reduce Emission of Respiratory Particles Generated by Speaking. *Environmental Science & Technology Letters* **9**, 557-560, doi:10.1021/acs.estlett.2c00210 (2022).

14 The United States National Institute for Occupational Safety & Health. Standard Respirator Testing Procedures: Determination of Inhalation Resistance TEB-APR-STP-0007, Revision 2.3. (2019).

15 Kim, J.-H. *et al.* Pressure drop of filtering facepiece respirators: How low should we go? *International Journal of Occupational Medicine and Environmental Health* **28**, 71-80, doi:10.13075/ijomeh.1896.00153 (2015).

16 Prudhvi Krishna, M. *et al.* Effectiveness of different facemask materials to combat transmission of airborne diseases. *Sādhanā* **46**, 119, doi:10.1007/s12046-021-01634-z (2021).

17 Pleil, J. D., Ariel Geer Wallace, M., Davis, M. D. & Matty, C. M. The physics of human breathing: flow, timing, volume, and pressure parameters for normal, on-demand, and ventilator respiration. *Journal of Breath Research* **15**, 042002, doi:10.1088/1752-7163/ac2589 (2021).
18 Ju, J. T. J., Boisvert, L. N. & Zuo, Y. Y. Face masks against COVID-19: Standards, efficacy, testing and decontamination methods. *Advances in colloid and interface science* **292**, 102435, doi:10.1016/j.cis.2021.102435 (2021).
19 World Health, O. Advice on the use of masks in the context of COVID-19: interim guidance, 5 June 2020. (World Health Organization, Geneva, 2020).
20 Zhao, M. *et al.* Household Materials Selection for Homemade Cloth Face Coverings and Their Filtration Efficiency Enhancement with Triboelectric Charging. *Nano Letters* **20**, 5544-5552, doi:10.1021/acs.nanolett.0c02211 (2020).
21 Newman, S. P., Pellow, P. G. D. & Clarke, S. W. Droplet size distributions of nebulised aerosols for inhalation therapy. *Clinical Physics and Physiological Measurement* **7**, 139-146, doi:10.1088/0143-0815/7/2/004 (1986).
22 Ortorex. *Portable Ultrasonic Nebulizer for Inhalation*, 2022).
23 Kooij, S., Astefanei, A., Corthals, G. L. & Bonn, D. Size distributions of droplets produced by ultrasonic nebulizers. *Scientific Reports* **9**, 6128, doi:10.1038/s41598-019-42599-8 (2019).

REVIEWERS' COMMENTS

Reviewer #2 (Remarks to the Author):

I would like to express my appreciation and congratulations to the authors. They have addressed all the previous feedback and revisions, resulting in a significantly enhanced and informative article. I believe that this revised version is now robust and instructive, making it a valuable resource for the development of new techniques in mask manufacturing and usage.
Adding a graph comparing your results with those from other papers in the supplementary material would indeed be an interesting addition. It would provide a valuable visual representation of the effectiveness of the technique developed in your study and demonstrate its usefulness in comparison to existing research. This comparison would further support the significance and applicability of your findings.

Reviewer #3 (Remarks to the Author):

Overall, I find the revisions to the manuscript have substantially improved it, and with some small additions, I believe that it will be suitable for publication.

The discussion of the potential role of electrostatics in the filtration mechanisms is appreciated. Electrostatics are generally more important in the filtration of sub-micron particles, without the benefit of the size distributions, I overlooked that mechanism.

My primary remaining concern is still around the size distributions. The figures show a similar mode for all materials tested between 0.1 and 0.6 micrometers (S4). However, these figures cut off the size distribution at a little more than 0.6 micrometers. I am concerned that there may be additional modes of larger particles that could be impacting the interpretation of the data. The similarities between the size distribution shown for pure water (a) and the other materials also indicate that these size distributions may not be completely representing the aerosol particles of interest. The product literature for the nebulizer used in this study indicates that it produces particles between 1 and 5 micrometers, however, it does not indicate if this is the wet drop size distribution or the dry particle size distribution, nor at what solution concentration. Since the Malvern Spraytec specifications indicate that it can measure particles sizes up to 2000 micrometers, it should be possible to extend these size distributions out to at least 10 or 20 micrometers, to ensure that the aerosolized volume is represented.

With the addition of the extended size distributions and any modifications to the analysis that might result from including those data, I believe that this paper will be suitable for publication.

Reviewer #4 (Remarks to the Author):

A new concept in face coverings has been demonstrated that utilizes functionalized microporous silica for the adsorption of bioaerosols. Furthermore, the revise to reviewer' comments was also reasonable, but I still have a few small questions. I considered this work worth considering for publication following some revisions as noted below.

1. Is the mask universal? Is it still useful for other viruses?
2. Have you considered the durability of masks? When wearing a mask, a large amount of water vapor is generated due to breathing. In this case, can silicon dioxide still be retained on cotton fibers?
3. The adsorption type belongs to type IV, and multi molecular layer adsorption will not occur, so how long will it fail to wear?

Reviewer comments are written in blue-coloured font.
Our responses are in black-coloured font.
Newly added text in the manuscript are shown in red-coloured font.

Response to REVIEWER COMMENTS

Reviewer #2:

I would like to express my appreciation and congratulations to the authors. They have addressed all the previous feedback and revisions, resulting in a significantly enhanced and informative article. I believe that this revised version is now robust and instructive, making it a valuable resource for the development of new techniques in mask manufacturing and usage.

Q1: Adding a graph comparing your results with those from other papers in the supplementary material would indeed be an interesting addition. It would provide a valuable visual representation of the effectiveness of the technique developed in your study and demonstrate its usefulness in comparison to existing research. This comparison would further support the significance and applicability of your findings.

Response: We appreciate the reviewer's suggestion and agree with the sentiment. However, it is very difficult to find studies which are directly comparable. In our research, we have focused on utilising a cloth-based substrate and shown the relative filtration efficiency enhancement that can be afforded by the addition of silica, whereas other studies in this general area may have a different emphasis (e.g., antiviral, antibacterial, thermal comfort, etc), use alternative test materials (and methods), or consider a total mask design (including optimisation of the substrate itself). As such, whilst a visual comparative summary would be useful, overall, we believe that in the absence of like-for-like data it could be misleading. Therefore, no changes have been made in this regard.

Reviewer #3:

Overall, I find the revisions to the manuscript have substantially improved it, and with some small additions, I believe that it will be suitable for publication.

The discussion of the potential role of electrostatics in the filtration mechanisms is appreciated. Electrostatics are generally more important in the filtration of sub-micron particles, without the benefit of the size distributions, I overlooked that mechanism.

My primary remaining concern is still around the size distributions. The figures show a similar mode for all materials tested between 0.1 and 0.6 micrometers (S4). However, these figures cut off the size distribution at a little more than 0.6 micrometers. I am concerned that there may be additional modes of larger particles that could be impacting the interpretation of the data. The similarities between the size distribution shown for pure water (a) and the other materials also indicate that these size distributions may not be completely representing the aerosol particles of interest. The product literature for the nebulizer used in this study indicates that it produces particles between 1 and 5 micrometers, however, it does not indicate if this is the wet drop size distribution or the dry particle size distribution, nor at what solution concentration. Since the Malvern Spraytec specifications indicate that it can measure particles sizes up to 2000 micrometers, it should be possible to extend these size distributions out to at least 10 or 20 micrometers, to ensure that the aerosolized volume is represented.

Q1: With the addition of the extended size distributions and any modifications to the analysis that might result from including those data, I believe that this paper will be suitable for publication.

> **Response:** We agree, and thank the reviewer for their detailed consideration of our work. Our original experiments focused on capturing the particle sizes which are known to be most penetrating (~ 0.3 µm). We no longer have access to the original particle sizing equipment (previously access was kindly provided courtesy of Malvern Panalytical), thus we have undertaken some additional experiments, obtained with a 6-channel handheld particle counter, to characterise the nebuliser output over a wider range up to 10 µm as suggested. Accordingly, we have updated Figure S4 showing the wider distribution data. As shown in the figure, smaller aerosol particle sizes up to ~0.3 µm are the most dominant.
>
> ***"Size distribution of bioaerosols***
> *An Orthorex mini ultrasonic nebuliser was used to generate aerosols from the test working solutions and used for all filter efficiency tests unless stated otherwise. The particle size distribution of the aerosol was measured for eight solutions at 0.1 mg/mL concentration. They were pure water, cytochrome c, BSA, ubiquitin, myoglobin, inactivated SARS-CoV-2 (1.3 x 10^5 PFU/mL), creatinine and caffeine. The results showed that all solutions had very similar distributions, as shown in Figure S4. The most frequent particle diameter band for all test solutions is ~0.3 µm. Characterisation of the nebuliser showed that most particles were smaller than 1 µm (Figure S4), and thus can be considered to be aerosolised, which is*

broadly consistent with the data reported by the manufacturer [1-3].The refractive indexes of all testing materials were very similar to that of pure water (Table S4), indicating that the laser light scattering for particle size distribution measurements was not affected by the internal properties of the aerosols."

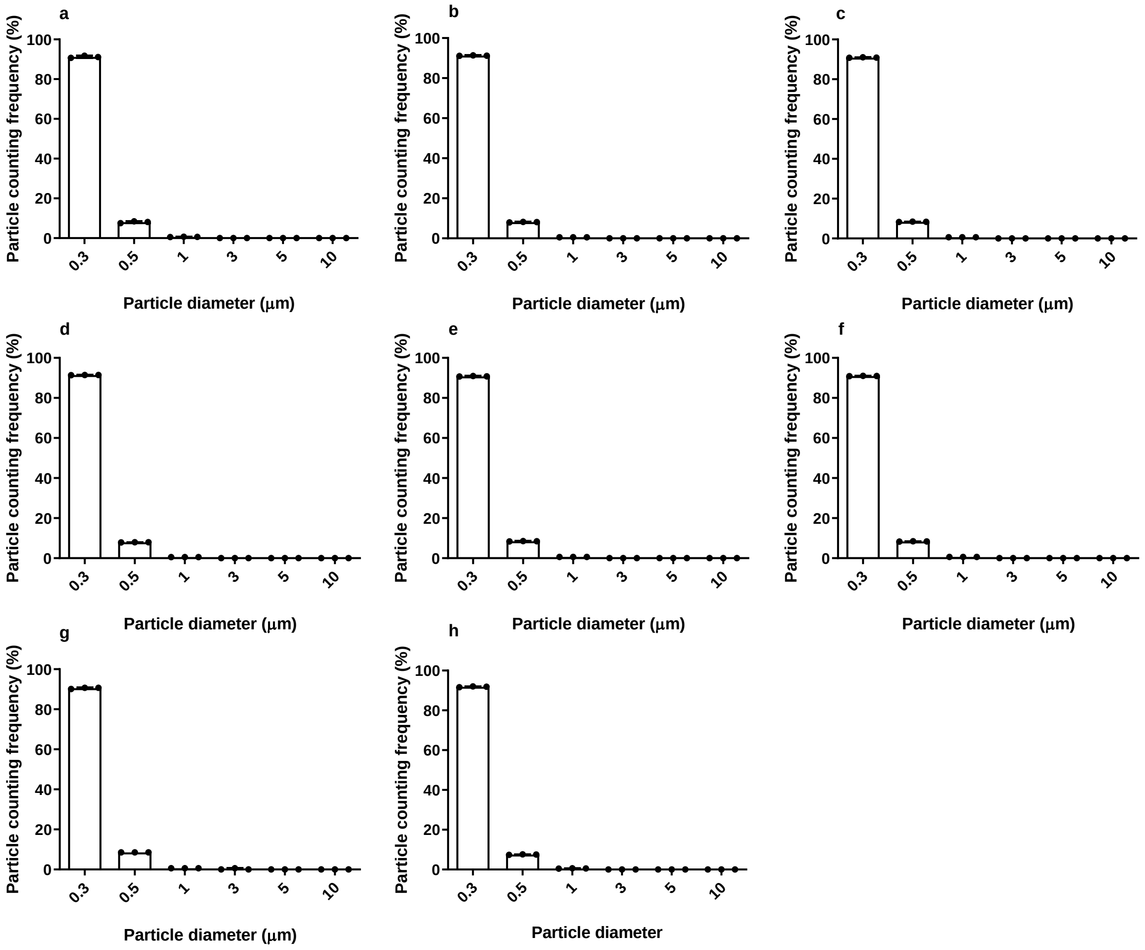

Supplementary Figure S4. Particle size distribution of aqueous aerosol containing 0.1 mg/mL of **a**, pure water; **b**, cytochrome c; **c**, BSA; **d**, ubiquitin; **e**, myoglobin; **f**, inactivated SARS-CoV-2 in buffer solution; **g**, creatinine; **h**, caffeine. They were generated with a nebuliser (ORTOREX™ Portable Ultrasonic Nebuliser) and measured with a handheld 6-channel dust particle counter which uses a laser diffraction system (TC-8200, Dongguan Huazhong Instrument Co., Guangdong, China). The most frequent band of particle diameter lies at ~0.3 μm in each case. The concentration of the inactivated SARS-CoV-2 in buffer solution used here was 1.3 x 10^5 PFU/mL, which was diluted 10 times from the original solution due to instrumental signal saturation. All data are derived from three independent experiments (mean ± SD, n = 3).

Reviewer #4:

A new concept in face coverings has been demonstrated that utilizes functionalized microporous silica for the adsorption of bioaerosols. Furthermore, the revise to reviewer' comments was also reasonable, but I still have a few small questions. I considered this work worth considering for publication following some revisions as noted below.

Q1: Is the mask universal? Is it still useful for other viruses?

Response: We thank the reviewer for this interesting line of questioning about the mask's capacity to effectively capture other viruses. The original motivation of our design was in response to the COVID-19 pandemic. As such our focus has been SARS-CoV-2. Based on the enhanced filtration efficiency for aerosolised proteins, in general, and SARS-CoV-2, specifically, we strongly suspect this approach should be useful for many other viruses, but we prefer not to speculate. Furthermore, there is the exciting possibility to tailor a mask for other viruses due to the tunability of silica by changing the particle size, pore size and functional groups. This is mentioned in the last paragraph of the ***Results and Discussion*** section:

"In conclusion, ……

There are several exciting possibilities for future work to explore and extend the proof-of-concept research demonstrated herein…………Moreover, ***the tunability of silica offers a plethora of further opportunities to bind to other analytes with improved selectivity, by changing the particle size, pore size and functional groups.*** *While this paper has focused on common face coverings, the utilisation of silica as a virion adsorbent shows excellent potential for use in face coverings, air filtration systems and even as a bioaerosol sampler."*

Q2: Have you considered the durability of masks? When wearing a mask, a large amount of water vapor is generated due to breathing. In this case, can silicon dioxide still be retained on cotton fibers?

Response: Durability is an important consideration but, as this is a proof of concept study, product development type activities are out of scope. Albeit Figure S3 does give a preliminary indication of stability. In any case, investigations of this type remain the subject of future work.

"In conclusion, an innovative concept in face coverings has been demonstrated that utilises functionalised mesoporous silica for the adsorption of bioaerosols. In this feasibility study, the well-known affinity of silica for some common proteins has been demonstrated in an aerosolised form showing filter efficiencies of 97%, 87%, 91 % and 95% for aerosolised myoglobin, BSA, ubiquitin and cytochrome c, respectively – with an average increase in filter efficiency of ~17% compared to an untreated blank. The addition of amorphous silica to a swab was also shown to decrease the amount of

aerosolised SARS CoV-2 that passed through the face covering by ~65%. The silica-coated substrate had nearly no impact on the pressure drop across the face covering. This approach enables increased filter efficiency performance whilst retaining the advantages of a 'breathable' substrate. There are several exciting possibilities for future work to explore and extend the proof-of-concept research demonstrated herein. Preliminary tests indicated that the silica-coated swabs remain useable for at least one month. Subsequent studies are warranted to consider longer-term durability more widely, including simulation of routine usage scenarios. Further experimentation and theoretical analysis should explore silica-bioaerosol interactions, within the context of air filtration, in greater detail……………."

Q3: The adsorption type belongs to type IV, and multi molecular layer adsorption will not occur, so how long will it fail to wear?

Response: We thank the reviewer for this comment. We do apologise for our incorrect use of the term "microporous" in the original manuscript that might have caused confusion. According to the IUPAC definition [4], the term "microporous" (pore diameter < 2nm) should be replaced by "mesoporous" (pore diameter =2-50 nm) (Figure S2a shows the mean pore diameter of our silica is 11.48 nm). The correct term, "mesoporous", has been used in the revised manuscript.

The Brunauer–Emmett–Teller (BET) isotherm in our manuscript displays a Type IV H2 hysteresis. A type IV adsorption isotherm is the result of capillary condensation taking place in mesopores after multilayer adsorption [4,5]. In the IUPAC classification, multilayer adsorption is a typical feature of a Type IV isotherm [4].

Since this project is a proof-of-concept study, we did not study how long the face covering will last in a simulated environment of normal human usage. This is the subject of future work. The corresponding change to the manuscript is as noted in our previous answer to Q2.

References

1 Newman, S. P., Pellow, P. G. D. & Clarke, S. W. Droplet size distributions of nebulised aerosols for inhalation therapy. *Clinical Physics and Physiological Measurement* **7**, 139-146, doi:10.1088/0143-0815/7/2/004 (1986).

2 Ortorex. *Portable Ultrasonic Nebulizer for Inhalation*, 2022).

3 Kooij, S., Astefanei, A., Corthals, G. L. & Bonn, D. Size distributions of droplets produced by ultrasonic nebulizers. *Scientific Reports* **9**, 6128, doi:10.1038/s41598-019-42599-8 (2019).

4 Sing, K. S. W. Reporting physisorption data for gas/solid systems with special reference to the determination of surface area and porosity (Recommendations 1984). *Pure and Applied Chemistry* **57**, 603-619, doi:doi:10.1351/pac198557040603 (1985).

5 Hattori, Y., Kaneko, K. & Ohba, T. in *Comprehensive Inorganic Chemistry II (Second Edition)* Vol. 5 (eds Jan Reedijk & Kenneth Poeppelmeier) 25-44 (Elsevier, 2013).
